# A Stochastic Model of the Yeast Cell Cycle Reveals Roles for Feedback Regulation in Limiting Cellular Variability

**DOI:** 10.1371/journal.pcbi.1005230

**Published:** 2016-12-09

**Authors:** Debashis Barik, David A. Ball, Jean Peccoud, John J. Tyson

**Affiliations:** 1 School of Chemistry, University of Hyderabad, Hyderabad, Telangana, India; 2 Virginia Bioinformatics Institute, Virginia Polytechnic Institute & State University, Blacksburg, Virginia, United States of America; 3 Department of Biological Sciences, Virginia Polytechnic Institute & State University, Blacksburg, Virginia, United States of America; Duke University, UNITED STATES

## Abstract

The cell division cycle of eukaryotes is governed by a complex network of cyclin-dependent protein kinases (CDKs) and auxiliary proteins that govern CDK activities. The control system must function reliably in the context of molecular noise that is inevitable in tiny yeast cells, because mistakes in sequencing cell cycle events are detrimental or fatal to the cell or its progeny. To assess the effects of noise on cell cycle progression requires not only extensive, quantitative, experimental measurements of cellular heterogeneity but also comprehensive, accurate, mathematical models of stochastic fluctuations in the CDK control system. In this paper we provide a stochastic model of the budding yeast cell cycle that accurately accounts for the variable phenotypes of wild-type cells and more than 20 mutant yeast strains simulated in different growth conditions. We specifically tested the role of feedback regulations mediated by G1- and SG2M-phase cyclins to minimize the noise in cell cycle progression. Details of the model are informed and tested by quantitative measurements (by fluorescence in situ hybridization) of the joint distributions of mRNA populations in yeast cells. We use the model to predict the phenotypes of ~30 mutant yeast strains that have not yet been characterized experimentally.

## Author Summary

The cell division cycle—the process by which a living cell makes a new replica of itself—is fundamental to all aspects of biological growth, development and reproduction. If cells make mistakes in cell cycle progression, they may die or give birth to aberrant progeny. Such mistakes are the root cause of serious human diseases such as cancer. Hence, we would like to understand how cells control cell cycle events and correct mistakes before they do serious damage. Yeast cells are especially suited to studying cell cycle progression because so much is known about the underlying molecular control system, and because yeast cells—being so small—are especially vulnerable to random fluctuations in molecular regulators of the cell cycle. Experimental studies have identified feedback signals in the regulatory network that appear to keep these fluctuations within manageable limits. To place these proposals in a rigorous theoretical framework, we present a stochastic model of the major feedback controls in the yeast cell cycle. Our model accounts accurately for a range of observations about cell cycle variability in wild-type and mutant cells, and makes a host of verifiable predictions about mutant strains that are seriously compromised in cell cycle progression.

## Introduction

Budding yeast (*Saccharomyces cerevisiae*) is a favorite organism for experimental studies of cell cycle regulation and for mathematical modeling of the control system. We now know dozens of genes that are intimately involved in regulating progression through the cell cycle in budding yeast [1–4], and there exist intriguing mathematical models of how the interacting proteins encoded by these genes control each stage of the process. Many different modeling strategies have been explored: continuous or discrete models, deterministic or stochastic models, and various types of hybrid models [5–11]. The most accurate and comprehensive of these theoretical descriptions are continuous/deterministic models, expressed in terms of nonlinear ordinary differential equations [5]. In their current state, these nonlinear ODE models account in quantitative detail for the phenotypes of wild-type yeast cells and of >200 mutant yeast strains [12, 13]

As good as the continuous/deterministic models are in describing the average properties of cell cycle progression in budding yeast, they must be generalized to deal with the inevitability of intrinsic and extrinsic noise affecting individual yeast cells and the concomitant cell-to-cell variability in progression through the cell division cycle. In recent years, experimentalists have collected much relevant data on the extent of this variability [14–17]; results that challenge theoreticians to develop accurate stochastic models of the molecular origins of the observed variability. In particular, yeast cells are very small (~30 fL at birth) and contain limited numbers of crucial molecules (1 copy of each gene in haploid cells, 5–10 copies of unique mRNAs per cell, and 500–5000 copies of each protein encoded by these mRNAs). Inevitable molecular fluctuations at the protein level may jeopardize the integrity of cell-cycle regulatory processes, with potentially catastrophic effects.

It is likely that the control system has feedback signals that are specifically involved in minimizing cell cycle variability, and there is intriguing experimental evidence that positive feedback at the G1/S transition plays this role in budding yeast cells [14]. In this paper we present a stochastic model of the effects of molecular noise on the regulatory network controlling the progression of budding yeast cell through the DNA replication-division cycle. The model is sufficiently detailed to address in quantitative terms the observed variability in cell cycle progression in wild type cells grown in different media and in ~20 mutant yeast strains of relevance to robust growth and division. The excellent quantitative fit between model simulations and published experimental data attests to the accuracy of the model. The model confirms that feedback signals in the network improve the reliability of the control system because the variability of progression through the cell cycle increases when crucial feedback signals are compromised by mutation. The regulatory network is most sensitive to temporal fluctuations in mRNA levels within cells, and we show that the model accurately captures these fluctuations by comparing predicted joint distributions of mRNA molecules encoded by seven different cell cycle genes with new measurements of these joint distributions in single yeast cells by fluorescence in situ hybridization.

The model also addresses an interesting phenotype, called “partial viability”, of some mutant strains, for which a fraction of cells in a population are able to grow, divide and make progeny, whereas the remaining cells in the population become arrested in the cell cycle, never divide, and eventually die. This phenotype is intrinsically stochastic and cannot be explained by a deterministic model of cell cycle progression, for which all cells must progress through the cycle in identical fashion, either dividing successfully or becoming arrested. Our stochastic model is in quantitative agreement with experimental observations on mutant strains known to be partially viable and predicts partial viability of novel strains that have yet to be characterized experimentally.

## Results

### The mathematical model

In a previous paper [6] we presented a simplified model of cell cycle progression in wild-type budding yeast cells. The simple model had S-phase and M-phase cyclins (called “ClbS” and “ClbM”), a ubiquitin-ligase complex (called Cdh1) for degrading ClbM, a transcription factor (SBF) for regulating expression of the *CLBS* gene, a phosphatase (Cdc14) for driving cells out of mitosis into G1 phase, two stoichiometric inhibitors (Whi5 and Net1; binding partners of SBF and Cdc14, respectively), and a “starter kinase” (Cln3) to trigger the G1-S transition when cells grow sufficiently large. (The model had three other unregulated phosphatases for dephosphorylating SBF, Whi5 and Net1.) In the model, ClbS and ClbM combine with kinase subunits (“Cdc28”) to form heterodimers with CDK activity, which is targeted to multiple phosphorylation sites on each of their protein substrates. The phosphorylation of these substrates modifies their catalytic activities, which feedback on the cyclin:Cdc28 dimers in the network. Multisite phosphorylation of CDK target proteins accounts for the “ultrasensitive” response curves that are crucial to the signal-processing characteristics of the control system.

**Fig 1:**
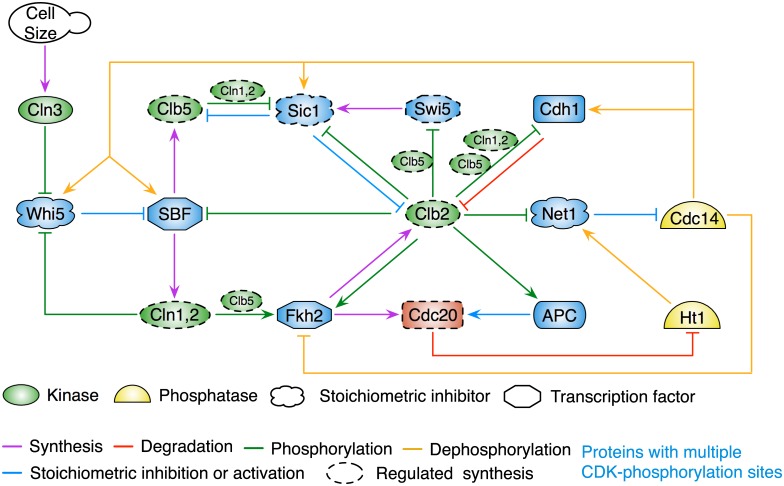
Schematic diagram of cell-cycle regulatory interactions in budding yeast. The icons represent the sixteen regulated proteins in our model of the budding yeast cell cycle. (The seventeenth component is a generic phosphatase, Hbf, that is synthesized and degraded constitutively but fluctuates nonetheless in the stochastic version of the model.) In this “influence diagram” the barbed arrows and blunt-headed lines represent positive and negative influences, respectively. Mechanistic details of the model are given in **Figures A-F** in S1 Text.

This reaction network was described in terms of elementary chemical reactions and simulated by Gillespie’s stochastic simulation algorithm (SSA) [18]. The simple model [6] was sufficient to describe in quantitative detail many aspects of stochastic progression through the wild-type cell cycle, but it lacked the genetic complexity necessary to simulate the behavior of a variety of interesting mutant strains of budding yeast. Our intention in this paper is to extend the simple model to a more complex network of interacting genes and proteins: a network rich enough to address important questions of stochastic cell cycle progression in yeast mutants. The underlying structure of our simple model was a bistable switch (formed by the double-negative feedback loop between ClbM and Cdh1) embedded within two negative feedback loops: one to drive the Start transition (ClbS ─┤Cdh1 ─┤ClbM ─┤SBF → ClbS) and a second to drive Exit from mitosis (ClbM ─┤Net1 ─┤Cdc14 → Cdh1 ─┤ClbM) [4]. In addition, we included a positive feedback loop (ClbS ─┤Whi5 ─┤SBF → ClbS) that plays a crucial role in the Start transition. Our new model (see Fig 1) retains these basic feedback loops and provides additional mechanistic details that are necessary to describe the properties of mutants. (In the ‘Budding yeast cell cycle’ subsection of **Materials and Method** we summarize the molecular biology of budding yeast cell cycle controls, in order to provide the necessary background for our model.) “ClbS” and “ClbM” are separated into three classes of cyclins: Cln1,2 (primarily responsible for bud emergence), Clb5,6 (primarily responsible for initiation of DNA synthesis) and Clb1,2 (required for mitotic events). (As in earlier models of the budding yeast cell cycle, we combine Clb5 and Clb6 proteins into a single variable, “Clb5”, and similarly for “Clb2” = Clb1 + Clb2.) The ubiquitin ligase, “APC:Cdh1”, is separated into two distinct forms: (1) APC:Cdc20 is active during the metaphase-anaphase transition, and (2) APC:Cdh1 is active during telophase and throughout G1 phase. The stabilizing effect of APC:Cdh1 during G1 phase is backed up by a cyclin-dependent kinase inhibitor called Sic1. (“Sic1” in the model is a composite variable of Sic1 protein and the kinase-inhibitor domain of Cdc6.) Finally, in addition to SBF, the extended model has two other transcription factors: Fkh2 (a transcription factor driving expression of *CLB2*, *CDC20* and *SWI5* genes) and Swi5 (driving expression of *SIC1*).

These added complexities are necessary, as we said, to describe the stochastic phenotypes of yeast strains deleted for specific genes (e.g., *cln2Δ*, *clb2Δ*, *clb5Δ*, *sic1Δ*) and/or carrying over-expression alleles (e.g., *GAL-SIC1* or *CLB2-dbΔ*). For example, the bistable switch in the simple model is broken in *cdh1Δ* cells, but not so in the extended model, where Cdh1 has a “back-up” inhibitor, Sic1.

Since Cdc28 is abundant and its level does not change during the cell cycle, we do not include it in the model. The number of each cyclin:Cdc28 heterodimer is assumed to be, simply, the number of molecules of the relevant cyclin.

The schematic diagram of the model in Fig 1 is portrayed in full molecular details in **Figures A-F** in S1 Text. Further details of the model are given in **Materials and Method** section. The variables of the model (17 proteins and 17 mRNAs) are defined in **Table A** in S1 Text. Of the 17 genes in the model, seven (*CLN1*, *CLN2*, *CLB5*, *CLB2*, *SIC1*, *SWI5* and *CDC20*) are regulated by transcription factors in the model. The differential equations used to simulate the deterministic version of the model are displayed in **Table B** in S1 Text, and numerical values are assigned to the 157 parameters of the model in **Table C** in S1 Text. Initial values of the model variables are given in **Table A** in S1 Text.

The cell division cycle must be coupled to cell growth to achieve balanced growth and division (i.e., homeostatic control of the average size of proliferating yeast cells). It is well-known that this “size-control” of budding yeast cells is closely connected to expression of the *CLN3* gene and transport of Cln3 protein into the cell nucleus [19–22]. To account for these facts, we assume, as before (Barik et al, [6]), that the translational rate of *CLN3* mRNA is proportional to the square of cell volume, *V*^2^. For all other proteins in our model, translational rate is strictly proportional to cell volume (i.e., to the number of ribosomes in the cell). A recent paper by Schmoller et al [23] provides evidence that “size control” in budding yeast is due more to dilution of Whi1, a Start inhibitor, than to accumulation of Cln3, a Start activator. We do not expect that these two alternative mechanisms of size control in budding yeast will differ markedly in the statistical properties of cell cycle progression that we study in this paper. However, we intend to carry out a detailed comparison of the two mechanisms in a later modeling study.

In the model, yeast cells are assumed to grow exponentially, *V* = *V*(0)*e*^*μt*^, with specific growth rate, *μ*. Hence, the mass doubling time of the culture is ln(2)/*μ*. In Table 1 we list growth parameters for three different media used in the model. The mass-doubling times in glucose and glycerol/ethanol media are consistent with the measurements in Di Talia et al [16]. We choose an intermediate mass-doubling time for growth in galactose medium.

**Table 1:** The growth rate and mass doubling time of yeast cells in different media.

Carbon Source	Mass-doubling Time (min)	Specific Growth Rate (min^-1^)
Glucose	99	0.00700
Galactose	148	0.00467
Glycerol-Ethanol	174	0.00398

All the reactions of the model are governed by mass-action rate laws, so the model can be simulated accurately either as a set of deterministic, nonlinear ordinary differential equations or as a set of stochastic reactions that fire sequentially according to given propensities. For deterministic simulations we use one of the ODE solvers in the Parameter Estimation Toolkit (PET) (http://mpf.biol.vt.edu/pet/), and for stochastic simulations we use a custom implementation of Gillespie’s SSA [18].

#### Deterministic simulations

**Fig 2:**
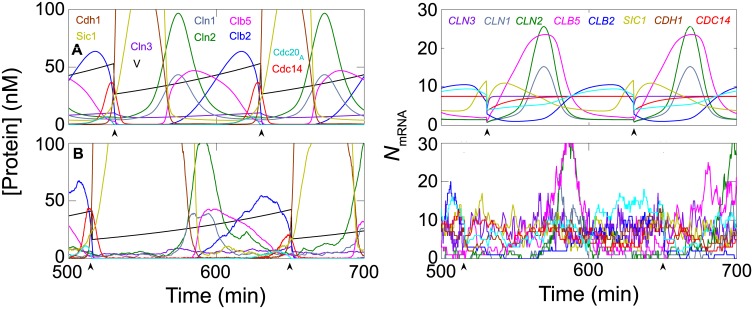
Deterministic and stochastic simulations of the model. **A (top panels).** Deterministic simulation of the changing concentrations of a representative sample of the proteins in the model (left) and the changing numbers of mRNA molecules for some of the genes (right). Cell volume, V(t) (in fL, black line), increases exponentially during each cycle and drops by a factor of 2 at cell division (indicated by arrowheads in each panel). The cell-cycle regulated genes show oscillatory dynamics of mRNAs, whereas for unregulated genes (e.g. *CLN3*, *CDH1*, *CDC14*) the mRNA level stays constant throughout the cell cycle. **B (bottom panels).** Stochastic simulations, in the same format as the top panels. The numbers of protein molecules N in a cell of volume V (in fL) has been converted into concentration C (in nM) by the equation C = 1.67 N/V. Hence, a concentration of 100 nM in a cell of volume 50 fL corresponds to ~3000 molecules.

A deterministic simulation of the model, for the parameter values given in **Table C** in S1 Text, is illustrated in Fig 2A. A newborn cell in G1 phase contains very low levels of cyclin proteins (except Cln3) and high levels of Cdh1 and Sic1, the stabilizers of G1 phase of the cell cycle. As the cell grows, the concentration of Cln3 increases, and Whi5 starts to be phosphorylated. Inactivation of Whi5 leads to activation of SBF and eventually to an autocatalytic rise of Cln1,2 proteins. Cln1,2-dependent kinases phosphorylate Cdh1 and Sic1, resulting in inactivation of Cdh1 and degradation of Sic1. As Sic1 level drops, active Clb5 is released, which drives the cell into S phase. Somewhat later in the cycle, Clb2 level starts to increase autocatalytically, as Clb2 phosphorylates and activates its own transcription factor, Fkh2. Clb2-kinase then drives the cell into mitosis and primes the cell for exiting mitosis by upregulating Cdc20 and phosphorylating the APC. Normally, Exit would be inhibited by the mitotic checkpoint complex and by a powerful protein phosphatase (PP2A:Cdc55), but we have chosen not to include these components in the present model. (They will be included later, in a model that focuses on “checkpoint controls,” which is not the subject of this paper.) The present model proceeds “automatically” through mitosis into anaphase (activation of APC:Cdc20) and telophase (activation of Cdc14). Cdc14 dephosphorylates Swi5, Sic1 and Cdh1, leading to a rapid accumulation of Sic1 and activation of the APC:Cdh1 pathway for Clb2 degradation. The rapid and complete loss of Clb2-kinase in telophase allows the cell to divide and the progeny cells to lock into the stable G1 steady state.

#### Stochastic simulations

A stochastic simulation of the model, for the parameter values given in **Table C** in S1 Text, is illustrated in Fig 2B. This simulation is one “realization” of a stochastic sequence of events that follows daughter cells (buds) from one generation to another (for details, see Materials and Methods). On the left we plot protein concentrations, which correlate closely with the deterministic time-courses in panel A. On the right we plot numbers of mRNA molecules of genes whose expression is modulated during the cell cycle. These numbers fluctuate considerably around the levels predicted by the deterministic model in panel A. Such large fluctuations are to be expected for such small numbers of molecules of each species. The full stochastic model gives an accurate reflection of the magnitude of fluctuations at the protein level that are induced by these fluctuating numbers of mRNA molecules.

### Variability in cell cycle progression and size control

**Fig 3:**
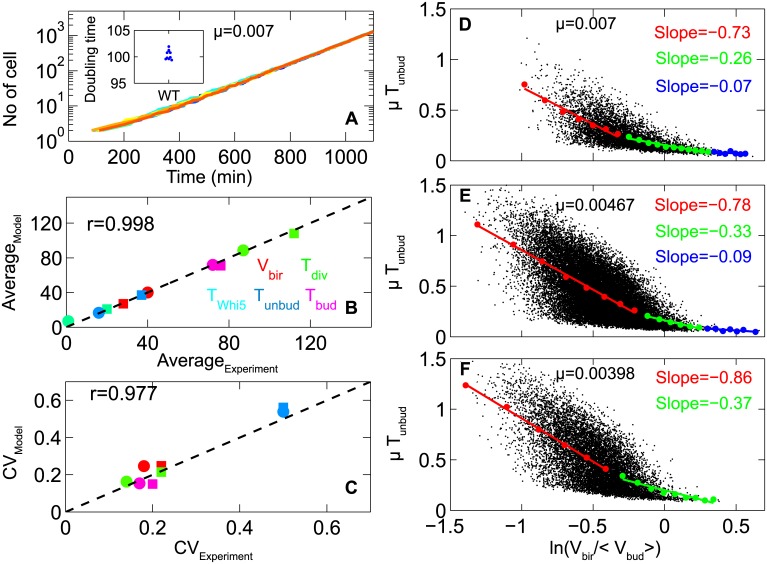
Cell cycle noise and size control in daughter cells. **A.** We plot the total number of cells in a “computational culture” of WT budding yeast as a function of time. The semilog plot clearly shows exponential increase of cell numbers. Lines of different colors represent repeat runs with the same initial conditions. The simulations were run with specific growth rate, μ = 0.007 min^−1^, representing glucose medium. **Inset**: scatter plot of the number doubling time of each computational culture. **B and C.** The average and CV (coefficient of variation) of some cell cycle properties for the computational cultures are correlated with experimental data [16]. **D, E and F.** Joint distributions of μT_unbud_ with ln(V_bir_) for three different growth media: μ = 0.007 min^−1^ (glucose), 0.00467 min^−1^ (galactose) and 0.00398 min^−1^ (glycerol-ethanol). V_bir_ is normalized by the average volume at budding (V_bud_). Solid black circles are individual cells and colored circles are the average values of μT_unbud_ over a small interval of ln(V_bir_) (referred to as “binned data” points). Binned data points are fitted with straight lines, and the slopes of the fitted lines are given inside each plot. The slopes are characterized as large (red), intermediate (green), and small (blue), and they correspond to “strong”, “weak” and “no” size control, respectively.

Using our custom-made stochastic simulation program (Materials & Methods), we generated a population of mother and daughter cells, expanding over time, starting from one progenitor cell at *t* = 0. We ran our simulation multiple times to create ‘computational replicates’ of the culture, in order to generate a statistical ensemble of cells from which to compute the distributions of various cell-cycle related properties. For example, in Fig 3A we show the increase in cell number for several simulations of wild-type cells growing in glucose medium. The inset shows that the cultures are growing with a number-doubling time of ~100 min, which is very close to the mass-doubling time of 99 min. (See **Figure G** in S1 Text for stochastic simulations of cell cultures growing on galactose and glycerol-ethanol media.)

During the course of stochastic simulations, we recorded various cell-cycle properties of individual mother and daughter cells: volume at birth (*V*_bir_), volume at budding (*V*_bud_), volume at division (*V*_div_), cell cycle duration (*T*_div_), duration of Whi5 retention in nucleolus (*T*_Whi5_), duration of unbudded phase of the cycle (*T*_unbud_), age at initiation of DNA synthesis (*T*_g1_), duration of budded phase (*T*_bud_ = *T*_div_ − *T*_unbud_) and duration of SG2M phase (*T*_sg2m_ = *T*_div_−*T*_g1_). In Fig 3B and 3C we show that the statistical descriptors of these cell-cycle properties (mean and CV = coefficient of variation = standard deviation / mean) agree closely with the corresponding experimental values [16]. (See **Table E** in S1 Text for all data.)

Budding yeast cells divide asymmetrically producing large mother and small daughter cells. Daughter cells take a long time to execute the Start transition compared to mother cells because they are so much smaller, on average, at birth. This delay is evidence of a strong size-control mechanism in daughter cells, as measured by Di Talia et al. [16]. Our previous stochastic model of the budding yeast cell cycle [6] captured their experimental observations quite well. We confirm these calculations with the present model (Fig 3D) and extend them to populations of cells growing in galactose (Fig 3E) and glycerol-ethanol (Fig 3F). In all three growth conditions, we find that, for the smaller cells in the population (*V*_bir_ < median(*V*_bir_)), the time spent by cells before the Start transition (*T*_unbud_) is strongly negatively correlated to size at birth (*V*_bir_). The model predicts that the size-control mechanism is more strongly enforced for daughter cells growing on ever poorer carbon sources (glycerol-ethanol poorer than galactose poorer than glucose). The model also confirms that for mother cells growing on glucose, the negative correlation between *T*_unbud_ and *V*_bir_ is not so strong (see **Figure H** in S1 Text**)** because most mother cells are large enough at birth to satisfy the critical size requirement for Start with little or no time delay.

In **Figure I** in S1 Text, we display the full histograms of cell cycle attributes (*V*_bir_, *V*_div_, *T*_div_, *T*_whi5_, *T*_unbud_, *T*_g1_ and *T*_bud_) for wild-type mother and daughter cells in each of the three growth media.

### Variability in mRNA abundances

**Fig 4:**
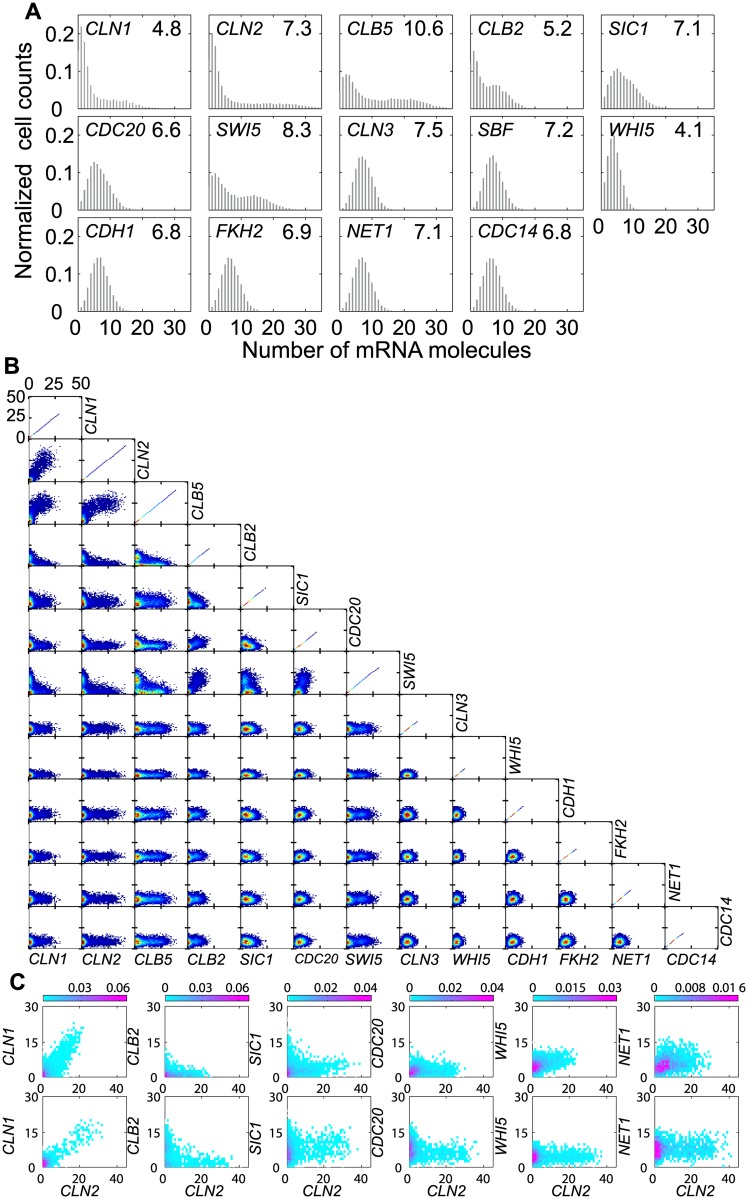
mRNA histograms for a population of budding yeast cells. **A.** Simulations for μ = 0.007 min^−1^ mimicking growth in glucose medium. The average number of mRNA molecules, <N>, for each gene is reported on the histogram. **B**. Joint distributions of mRNAs in individual cells. **C.** Comparison of model-predicted joint distributions (bottom row) with experimentally measured joint distributions (top row). For details of the experimental measurements, see Materials and Methods.

Simple statistical models of gene expression [24, 25] suggest that gene expression noise at the protein level depends critically on the average abundance of mRNA molecules and the average life times of mRNA and protein. For most genes in the model, we have taken half-life values of their mRNAs in the range of 5–10 min, consistent with the experimental observations of Miller et al [26]. The exceptions are *CLN1*, *CLN2* and *CLB5*. Although Miller et al reported that the half-lives for these mRNAs are 6.0, 7.4 and 6.1 min, we have assigned half-lives of 3 min for each of these mRNAs, in order to reduce the noise predicted by the model at the protein level. The transcription rates are chosen so that the average abundances of mRNAs for a population of cells match the experimental observations of Ball et al [27]. In Fig 4A we plot histograms of mRNAs species as predicted by the model for an asynchronous population of cells. Those genes that are constitutively active in the model (*CLN3*, *WHI5*, *CDH1*, *FKH2*, *NET1* and *CDC14*) show expected Poisson distributions of mRNA abundances, whereas regulated genes (*CLN1*, *CLN2*, *CLB5*, *CLB2*, *SIC1*, *CDC20* and *SWI5*) show non-Poisson mRNA distributions, consistent with single-cell experimental observations [27, 28]. The variabilities of mRNA for G1/S-regulated genes (*CLN1*, *CLN2*, *CLB5*) are large compared to other regulated genes; whereas *CLB2* appears to be more tightly regulated [27, 28]. Although the mRNA histograms of *SIC1* and *CDC20* may appear to be Poisson, they are regulated genes and we find that two-component Poisson distributions fit these histograms better than simple Poisson distributions, and these computed histograms closely match single-cell quantification of mRNA abundances by fluorescence in situ hybridization [27].

In Fig 4B we present computed pair-wise correlations of mRNA abundances for all the genes in our model. As expected, the joint distributions of all pairs of unregulated genes show circular regions of correlation. The joint distributions of all pairs of regulated genes show the expected causal relationships among the genes. For example, *CLB2*, a G2/M gene, is anti-correlated to the G1/S genes, *CLN1*, *CLN2* and *CLB5*; whereas the G1/S genes are positively correlated to each other. The joint distributions of regulated to unregulated genes are spread in the direction of the regulated gene, owing to the large variability in the expression of regulated genes.

We have measured the joint distributions of *CLN2* mRNA with six of these cell cycle genes: four regulated mRNAs (*CLN1*, *CLB2*, *SIC1* and *CDC20*) and two unregulated mRNAs (*WHI5* and *NET1*); see Fig 4C. The agreement between the predicted and measured joint distributions is quite good, considering that we have not adjusted the values of the model parameters to improve the fit of these joint distributions.

### Variability in protein abundances

**Fig 5:**
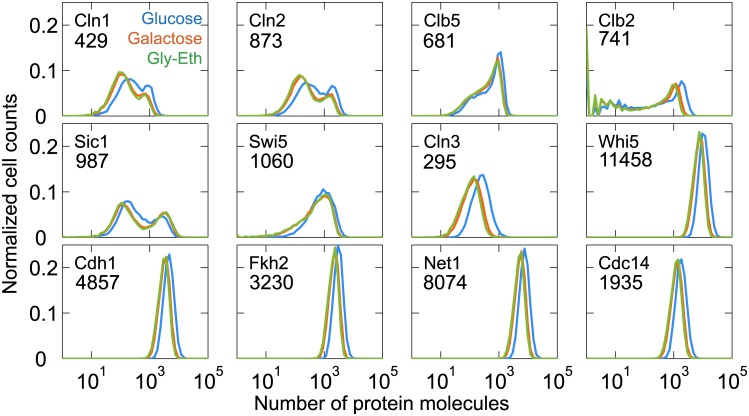
Model-predicted histograms of proteins for a population of budding yeast cells. Simulations under different growth conditions: blue, μ = 0.007 min^−1^ (glucose); red, μ = 0.00467 min^−1^ (galactose); green, μ = 0.00398 min^−1^ (glycerol-ethanol). The average number of protein molecules per cell, <N>, is indicated in each panel for the glucose-medium simulation.

Similar to mRNA, we quantified the proteins in our model for an asynchronous population of cells grown under three different growth conditions (Fig 5). As expected, regulated proteins (Cln1, Cln2, Clb5, Clb2, Sic1, and Swi5) show broad, often bimodal distributions, whereas unregulated proteins show narrow unimodal distributions. Regulated proteins are abundant only in a certain phase of the cell cycle, for example, Cln1/2 levels are high in G1 phase and low in SG2M phase, whereas Clb2 level is low in G1 and high in SG2M phase. Cells sampled from an asynchronous population will consist of cells at all phases of the division cycle; some with high protein levels and some with low levels. Thus, regulated proteins are expected to exhibit bimodal distributions, or significantly skewed distributions (e.g., Swi5 in Fig 5). (In Fig 5 we are reporting the total amount of each protein, adding together molecules that are free, that are bound in complexes, and that are phosphorylated to any degree, as might be measured by a fluorescent antibody that does not distinguish phospho-epitopes.) Because average cell volume becomes smaller as cells are grown on progressively poorer carbon sources, the protein histograms shift to the left as cells are shifted from glucose to galactose to glycerol-ethanol media. The relative heights of the two peaks of the bimodal distributions of regulated proteins also change with growth medium. This effect is most noticeable for Cln1 and Cln2, for which the high-abundance peak is more prominent in glucose medium compared to the poorer carbon sources, indicating that the fraction of total cycle time when SBF is active (i.e., Cln1,2 being produced) is greater when cells are nourished by glucose compared to galactose or glycerol-ethanol. The population-average numbers of all the proteins in the model vary from a few hundred to several thousand in glucose medium (Fig 5). These numbers are in good agreement with the experimental values obtained from genome-wide measurements [29], which is to be expected because we have chosen the protein synthesis rates in **Table C** in S1 Text to match these experimental values, given values for protein degradation rates taken from Belle et al [30].

### Positive feedback regulation and noise reduction

From the previous section we found that cell-to-cell variability of cyclin proteins is quite large. Because cyclin proteins drive yeast cells through key events of the cell cycle (budding, DNA synthesis, entry into and exit from mitosis), we might expect that the timing of these events would be equally noisy. However, single-cell experimental data [16] and our model calculations suggest that many events are not as variable as the proteins that regulate them. If so, then there must be some mechanism(s) by which yeast cells reduce the variability of key cell cycle events. How does protein noise get filtered at the functional level in a yeast cell?

For small gene regulatory networks, transcriptional noise in upstream gene expression gets amplified as it propagates to downstream gene expression [24, 31]. Both theoretical and experimental studies have shown that protein noise can be reduced by negative auto-regulation [32–34]. On the other hand, positive feedback loops in gene regulation—which are common in the regulation of cyclin genes—can either increase or decrease noise, depending on the mechanism of feedback regulation [35]. Hence, using our stochastic model of the yeast cell cycle, we have investigated the roles of positive feedback regulation of protein synthesis in modulating the variability of cell cycle events at the functional level.

#### Positive feedback in the synthesis of Cln1 and Cln2

The Cln-type cyclins exert strong influences on G1/S events, such as SBF activation, bud formation and initiation of DNA synthesis. As mentioned before, the activation of SBF is initiated by Cln3-dependent kinase, which phosphorylates Whi5, leading to the dissociation of Whi5 from SBF and nuclear export of Whi5. Active SBF promotes transcription of *CLN1* and *CLN2* genes, and Cln1,2 proteins support Cln3 in the phosphorylation of Whi5, thereby closing a positive feedback loop. Using time-lapse single-cell fluorescence microscopy, Skotheim et al [14] showed that this positive feedback loop (Cln1,2 ─┤Whi5 ─┤SBF → Cln1,2) is crucial to a rapid and coherent Start transition. Motivated by these experimental observations, we challenged our model to reproduce the role of the Cln1,2-dependent positive feedback loop.

To carry out this study, we simulated a clone of cells derived from a starting cell. After some time, *t*_stop_, we stopped the simulation and collected all the “cells” that are present in the population at this time point. For each cell *i* in this “extant” population of cells (1 ≤ *i* ≤ *n*), we collected information from the time it was born, at *t* = *t*_bir,*i*_ < *t*_stop_, until it divided at *t* = *t*_div,*i*_ > *t*_stop_. Over this single cycle of each simulated cell we recorded the cell’s age (*t* − *t*_bir,*i*_) at the time of the following events: *CLN2* activation, *RAD27* activation, budding, *CLB2* activation, and Whi5 exit from nucleus. We did this for wild-type (WT) cells, for *cln1Δcln2Δ* mutant cells, and for several other mutant strains.

**Fig 6:**
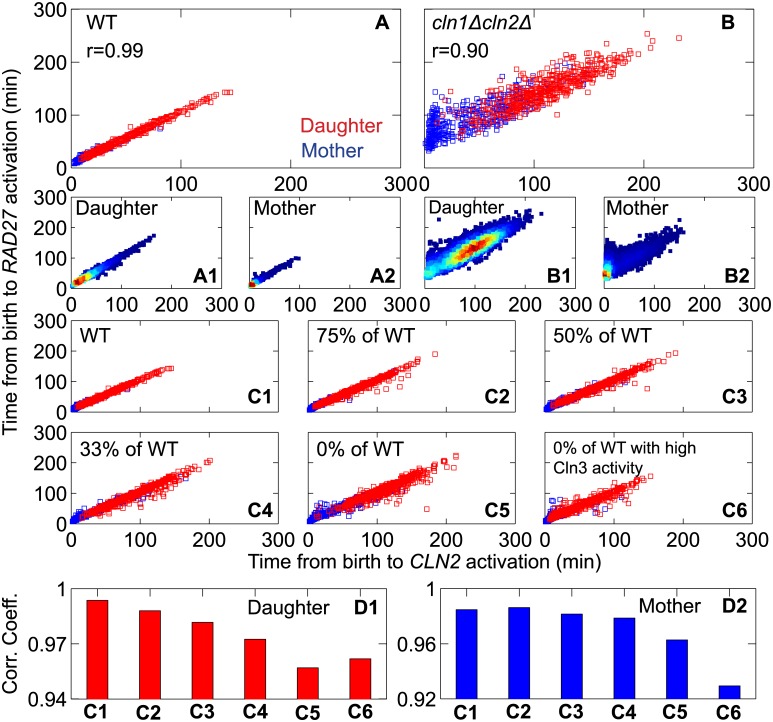
Positive feedback in *CLN2* activation and the coherence of entry into Start. **A and B.** Joint distributions of the times from birth to the activation of the genes for *RAD27* (T_bir,*RAD27*_) and *CLN2* (T_bir,*CLN2*_) are plotted for WT and *cln1Δcln2Δ* cells from single-cycle simulations for an extant population of 500 mother (blue) and 500 daughter (red) cells in glucose (μ = 0.007 min^−1^). In (**A1, B1**) and (**A2, B2**) the full data for daughter cells and mother cells are plotted separately. Compare panels A and B to Fig 2e and 2f, respectively, of Skotheim et al [14]. **C.** As the rate constant for phosphorylation of Whi5 by Cln1,2 (i.e., the strength of the Cln1,2-mediated positive feedback loop) is progressively decreased (in panels C1-C5), the coherence of gene activation upon entry into Start decreases. The coherence of entry into Start cannot be restored by increasing the phosphorylation rate of Whi5 by Cln3 (panel C6; compare to Fig 2i of Skotheim et al). **D.** The correlation coefficients of T_bir,*RAD27*_ and T_bir,*CLN2*_ for daughter cells and mother cells decrease as the strength of the positive feedback loop decreases. The numbers on the abscissa refer to the data in panels C1-C6.

*RAD27* belongs to a family of genes (the “G1/S regulon”) that are turned on by the G1/S cyclins (Cln1,2,3 and Clb5). In our model *RAD27* gene expression ([G_a,rd27_] in **Table B** in S1 Text) is activated by Cln1,2,3 and Clb5 with different efficiencies. We monitored the activities of the *CLN2*, *RAD27* and *CLB2* genes (**Figure J** in S1 Text), and when each gene reached a threshold activity (0.15 for *CLN2*, 0.2 for *CLB2* and 0.2 for *RAD27*) we recorded the time (since birth) of each gene-activation event. In WT cells, the activation times of *CLN2* and *RAD27* are very well coordinated (Fig 6A, 6A1 and 6A2), for both mother and daughter cells, with a combined correlation coefficient of *r* = 0.99. On the other hand, for *cln1Δ cln2Δ* double-mutant cells (Fig 6B, 6B1 and 6B2), these activation times are less well correlated (*r* = 0.90), indicating a loss of coherence between *CLN2* and *RAD27* gene expression. (In experiments, the expression of *CLN2* in a *cln2Δ* cell is measured, of course, by expression of a reporter protein driven by the *CLN2pr* promoter sequence.) For mother and daughter WT cells, the average activation times of *CLN2* are 12 (5) and 35 (41) min, respectively. (The experimental values, from [14] Suppl Table S3, are given within the parentheses.) In the double-mutant cells, which lack the positive feedback loop, these statistical measures increase significantly, to 29 (43) and 87 (83) min for mother and daughter cells, respectively. More importantly, the standard deviation (σ) of *CLN2* activation time is approximately two-fold larger in the double mutant cells compared to WT cells: for daughter cells, σ = 41 (47) min in the *cln1Δ cln2Δ* strain, compared to 24 (21) min in WT; for mother cells, σ = 32 (28) min in *cln1Δ cln2Δ* cells, compared to 10 (10) min in WT.

The activation of *CLN1*,*2* gene expression is abrupt (see **Figure J** in S1 Text) because of a positive feedback loop whereby Cln1,2-dependent kinases phosphorylate Whi5 on multiple sites, causing Whi5 to dissociate from SBF, freeing SBF to up-regulate the expression of *CLN1*,*2* and other genes of the G1/S regulon, of which *RAD27* is a representative example. The positive feedback loop sets a threshold concentration for Cln1,2 to initiate expression of the G1/S regulon. This threshold effect reduces the variability in the times of activation of *CLN1*,*2* and *RAD27* genes at Start (see **Figure J** in S1 Text, rows 1 and 3 compared to rows 2 and 4. To see how the variability of Start events depends on the strength of Cln1,2-mediated positive feedback, we decreased the rate constant for Cln1,2-dependent phosphorylation of Whi5 by a certain percentage of the WT rate constant. We find that the average times from birth to the activation of *CLN2* and *RAD27* genes are inversely proportional to the strength of the positive feedback loop (Fig 6C1–6C5). Furthermore, the coherence of the activation of these two genes consistently decreases with decreasing strength of positive feedback (more so for daughter cells than for mother cells) (Fig 6D1 and 6D2). This decoherence cannot be reversed by increasing the strength of Cln3-mediated phosphorylation of Whi5 (Fig 6C6, 6D1 and 6D2 (right-most bar)), as observed by Skotheim et al (their Fig 2i).

In agreement with Skotheim et al (their Suppl Table S3), the coherence of gene expression at Start is not lost in the single mutant strains (*cln1Δ* and *cln2Δ*), because the positive feedback loop is still intact, although a little weaker than in WT cells (**Figure K** in S1 Text). In glycerol-ethanol medium the positive feedback loop displays similar properties (**Figure L** in S1 Text), consistent with the experiments of Skotheim et al [14]. A complete comparison of model simulations and experimental observations is given in **Tables F and G** in S1 Text. We have also calculated the cumulative distribution functions (CDFs) of activation times of *CLN2* and *CLB2* genes for WT and *cln1Δ cln2Δ* cells simulated with *μ* = 0.007 min^−1^ and 0.00398 min^−1^, mimicking glucose and glycerol-ethanol medium (see **Figure M** in S1 Text), and these CDFs are similar to the experimental observations reported in [14].

#### Positive feedback in the degradation of Sic1

**Fig 7:**
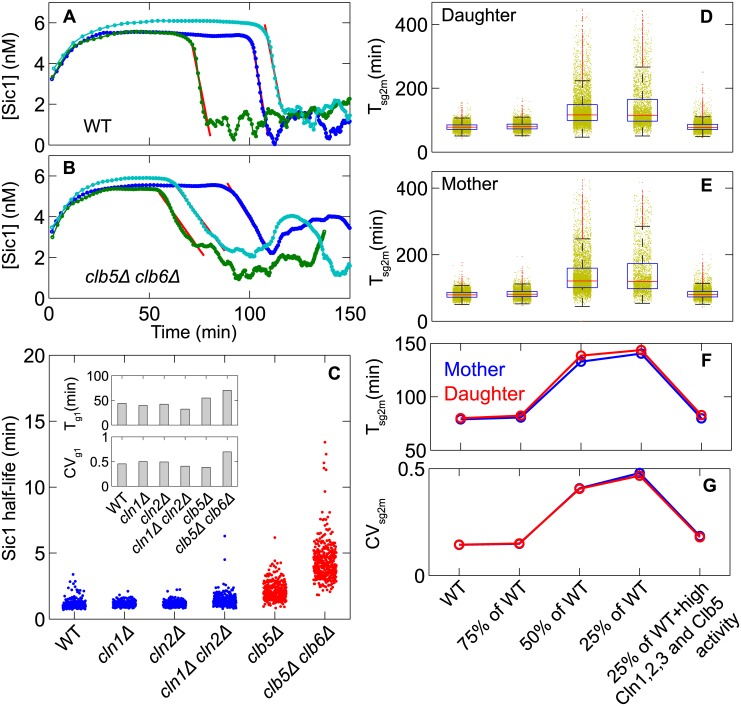
Positive feedback in SIC1 degradation and CLB2 activation. **A and B.** Temporal dynamics of total Sic1 are plotted for three WT and three *clb5Δclb6Δ* cells in glucose medium (μ = 0.007 min^−1^). The solid red lines fit the decay of Sic1 to a single exponential function to obtain the timescale of Sic1 degradation in individual cells. **C.** Half-lives of Sic1 in individual cells are clustered for WT and various mutant cells, showing that the deletion of S phase cyclin genes (*CLB5* and *CLB6* but not *CLN1* and *CLN2*) increases the average half-life and the variability of Sic1 degradation, causing an increase in the delay time and the variability of the onset of DNA synthesis (*T*_g1_) (**inset**). **D and E.** The durations of SG2M phase (*T*_sg2m_ = *T*_div_−*T*_g1_) from single-cycle simulations of an extant population of daughter and mother cells are plotted for WT cells, WT cells with decreased rate constant for the phosphorylation of Fkh2 by Clb2 (i.e., decreasing strength of the positive feedback for *CLB2* activation), and WT cells with the weakest positive feedback loop and increased rate constants for phosphorylation of Fkh2 by Cln1,2,3 and Clb5. In each plot, the horizontal red line represents the median, the blue box represents 25^th^ to 75^th^ percentile of data, the horizontal black lines represent edges of data points and outliers are plotted as red dots. **F and G.** The average and CV of *T*_sg2m_ are plotted for the data in D and E.

Yang et al [36] have recently investigated the noise reduction capability of a parallel positive feedback operating at Start in the budding yeast cell cycle. Sic1 is a stoichiometric inhibitor of Clb-type cyclins, and Clb-dependent kinases phosphorylate Sic1, rendering it susceptible to SCF-mediated degradation. Therefore, Sic1 and Clb-type cyclins are involved in a double-negative feedback loop (a variation on the “positive feedback” theme). Inspired by the experiments by Yang et al, we tracked the dynamics of total Sic1 (free + all phospho-forms) of individual yeast cells taken from an extant population of mother and daughter cells. As done in Yang et al, we fitted the decay of Sic1 with an exponential function (Fig 7A and 7B) to obtain the lifetime of Sic1 in individual cells, for WT cells and five mutant strains (*clb5Δ clb6Δ*; *clb5Δ*; *cln1Δ*; *cln2Δ*; and *cln1Δ cln2Δ*). We found that the average lifetime of Sic1 is independent of Cln1 and Cln2 but becomes slightly longer in *clb5Δ* cells and is longest in *clb5Δ clb6Δ* cells (Fig 7C). The variability in Sic1 lifetime is largest, intermediate and smallest in *clb5Δ clb6Δ*, *clb5Δ* and WT cells, respectively. The trends in these values are consistent with experiments, but the lifetimes of Sic1 in the model are shorter than in reality, because we chose the rate constants for Sic1 degradation used in our calculations before we were aware of these experiments by Yang et al.

As expected, the average time from birth to the initiation of DNA synthesis (*T*_g1_) is larger in the *clb5Δ clb6Δ* strain than in WT cells, both for mother cells (55.2 min versus 25.3 min) and for daughter cells (70.1 min versus 43.8 min) (see **Table H** in S1 Text). These values are consistent with the experimental observation of a 30 min delay in the onset of DNA synthesis in *clb5Δ clb6Δ* cells compared to WT cells [37]. In our model, the onset of DNA synthesis is delayed in *clb5Δ clb6Δ* cells because they must rely on the accumulation of Clb2, which requires clearing the cell of Sic1 (by Cln2-dependent phosphorylation) and activation of Fkh2, the transcription factor for Clb2 production. In addition to delayed onset of DNA synthesis, our model predicts that the variability of *T*_g1_ increases considerably in *clb5Δ clb6Δ* cells: the CV for *T*_g1_ increases from 0.45 to 0.70 for daughter cells and from 0.35 to 0.66 for mother cells, when WT cells are compared to *clb5Δ clb6Δ* cells (**Table H** in S1 Text). This increased variability is a reflection of the decoherence of Sic1 degradation in *clb5Δclb6Δ* cells compared to WT cells, which is a result of disrupting the double-negative feedback loop between Sic1 and Clb5,6 in the double-mutant cells.

#### Positive feedback in the synthesis of Clb2

Finally, our model predicts a similar effect of positive feedback on reducing the variability of SG2M phase. The duration of SG2M phase depends primarily on the dynamics of Clb2 accumulation. Clb2 synthesis is regulated transcriptionally by the Fkh2 transcription factor, whose activation is initiated by the G1/S cyclins and further promoted by Clb2 itself. Thus the Fkh2-Clb2 motif is a positive feedback loop with the potential to reduce variability in SG2M phase. To study the role of positive feedback on Fkh2 phosphorylation by Clb2, we quantified the duration of SG2M phase (*T*_sg2m_ = *T*_div_−*T*_g1_) in individual cells taken from an extant population of mother and daughter cells. We quantified *T*_sg2m_ for WT cells with basal and with decreasing strength of the positive feedback loop (i.e., rate constant for phosphorylation of Fkh2 by Clb2). We found that, for both daughter and mother cells, the average duration and the variability of *T*_sg2m_ increases with decreasing strength of positive the feedback loop (Fig 7D–7G). In contrast to the Cln1,2 positive feedback loop, here the effect of reduced strength of positive feedback can be reversed by increasing the efficiency of phosphorylation of Fkh2 by G1/S cyclins. We attribute this difference to the fact that Fkh2 has only two CDK-dependent phosphorylation sites, making the Fkh2 inactive-to-active transition a poor switch.

In all three cases considered in this subsection, we observe that the variability in a certain cell cycle event is negatively correlated with the strength of the positive feedback regulating the event. In conjunction with ultra-sensitivity, positive feedback creates a strict threshold in signal response creating a ‘gate’ for activation or inactivation. Due to the signaling threshold, noise in the activation or inactivation of the event is reduced. Without positive feedback the threshold effect is lost, and variability of the event increases.

### Simulation of partial viability of certain mutant strains

Apart from specific regulatory connections in the cell cycle control network, we wanted to get a global picture of variability of cell cycle properties in a variety of mutant strains, with a view to identifying proteins that might play important roles in controlling variability in addition to their known functions driving cell cycle progression. To this end we simulated populations of stochastically fluctuating cells of various combinations of mutant genes in glucose and galactose medium. A full list of all mutant strains that we simulated is given in **Table D** in S1 Text, along with the parameters that were modified to simulate each mutant strain. For comparison purposes, we provide deterministic simulations of all mutant strains in the S1 Data Set File appended to this paper.

#### Glucose medium

**Fig 8:**
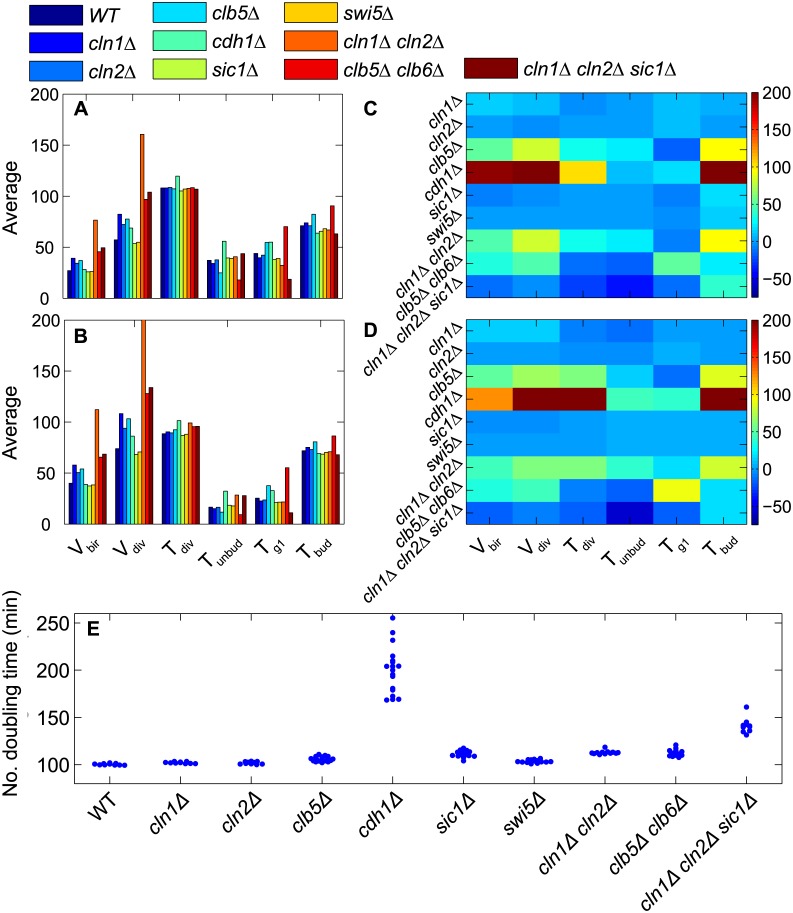
Mutant phenotypes in glucose medium. **A (daughter) and B (mother) cells.** The average of six cell cycle properties obtained from ‘computational cell culture’ simulations of WT cells and nine different mutant strains in glucose medium (μ = 0.007 min^−1^). Cell volumes in fL and phase durations in min. **C (daughter) and D (mother) cells.** The color bars indicate the percentage change in CV, relative to WT cells, of selected cell cycle properties (bottom) for a variety of mutant strains (left side). **E.** The number doubling times of ‘computational cell cultures’ are clustered for WT and mutant strains, showing largest variability among cultures of *cdh1Δ* cells.

In Fig 8 we report our results from stochastic calculations for all mutant strains that are known to be viable when growing in glucose medium [5]. The average properties of cell cycle progression predicted by our stochastic model are consistent with known experimental phenotypes (Fig 8A and 8B). For example, for the following mutant strains, the average size of daughter cells at birth is larger than WT daughter cells: *cln1Δ* (1.4×), *cln2Δ* (1.3×), *cln1Δ cln2Δ* (2.8×), *clb5Δ clb6Δ* (1.7×) and *cln1Δ cln2Δ sic1Δ* (1.8×); by multiplicative factors that are consistent with observations of [37] and [38]. The increase in average cell size is due to the fact that budding is delayed in the absence of G1 cyclin(s), allowing cells to grow larger compared to WT cells. For *sic1Δ* cells, budding is delayed compared to the onset of DNA synthesis: *T*_unbud_ is the same as WT cells but *T*_g1_ is shorter compared to WT cells [39]. A similar phenotype is observed in *swi5Δ* cells. The stoichiometric inhibitor, Sic1, normally sequesters Clb5, thereby ensuring timely release of Clb5 upon phosphorylation and subsequent degradation of Sic1. In absence of Sic1, active Clb5 accumulates prematurely, advancing the onset of DNA synthesis compared to WT cells. In all the *cln* mutants, budding is delayed, and also in *cdh1Δ* cells, due to high Clb2 activity, which shuts down synthesis of Cln1,2 by phosphorylating SBF. On the other hand, *T*_g1_ is increased in *clb5Δ* (54 min) and *clb5Δ clb6Δ* (70 min) daughter cells, as observed by [37]. See **Table H** in S1 Text for full data.

In Fig 8C and 8D we present the percent change in variability of cell cycle properties under genetic mutations. The model predicts that almost all the properties of *cdh1Δ* cells are highly noisy, particularly the duration of budded phase. *T*_bud_ is highly variable because Cdh1 is a strong inhibitor of Clb2 and, in its absence, exit from mitosis becomes sloppy because Sic1, a stoichiometric inhibitor of Clb2, is not so efficient as Cdh1 in ridding the cell of Clb2 activity in telophase. We also find that many *cdh1Δ* cells (45%) are arrested in various phases of the cell cycle (32% unbudded, 10% unreplicated DNA, 3% M phase). Because so many cells fail to complete the cell cycle, the *cdh1Δ* strain (in our simulations) grows quite slowly (Fig 8E) with average number doubling time of 197 min (and there is significant variation from one run to another; CV = 0.12). Our results suggest that Cdh1 plays a major role in reducing cell-to-cell variability in cell cycle progression.

Cdh1 plays such a significant role in our model because Cdh1, along with Sic1, stabilizes the G1 phase of the cell cycle by keeping Clb-dependent kinase activity low until after the Start transition. In the absence of Cdh1 (*cdh1Δ*), Sic1 is the only stabilizer of G1 phase, and, in the current parametrization of the model, Sic1 is a weaker inhibitor of Clb-dependent kinases than Cdh1. Therefore, in *cdh1Δ* cells, Clb1/2 cyclins arise prematurely, causing early inactivation of SBF, resulting premature repression of *CLN1*, *CLN2* and *CLB5* gene expression. Thus, many *cdh1Δ* cells fail to form a bud or initiate DNA replication, causing a large fraction of cells to arrest in G1. In addition, because Sic1 is the only inhibitor of Clb1/2 in *cdh1Δ* cells, exit from mitosis is also hampered, resulting in increased variability in the budded phase of the cell cycle, which ultimately leads to large variations in cycle time and cell sizes at birth and division.

The *cdh1Δ* strain is known to proliferate more slowly than wild-type cells [40], but only by 30% (not two-fold slower as predicted by our simulations). This discrepancy between model simulations and experimental observations suggests that the relative roles played by Cdh1 and Sic1 in stabilizing G1 phase are incorrectly parametrized by the model. We are looking into this problem both experimentally and theoretically and plan to address it in detail in a later publication.

Predictably, variability in *T*_g1_ increases in *clb5Δ clb6Δ* cells due to loss of positive feedback, as mentioned previously. Compared to WT cells, *cln1Δ cln2Δ* cells are noisy in almost all cell cycle properties. As expected, because *CLN1* and *CLN2* genes have overlapping functions, the single-mutant strains *cln1Δ* and *cln2Δ* do not show increased variability compared to WT cells. The single-mutant strains *sic1Δ* and *swi5Δ* also show similar variation to WT cells, suggesting that Sic1 does not contribute greatly to exit from mitosis in the presence of functional Cdh1.

All the double and triple mutant strains proliferate more slowly than WT cells (Fig 8E), and among them *cln1Δ cln2Δ sic1Δ* cells show the greatest variability in number doubling time from one culture to another. (See **Table H** in S1 Text for full data.)

#### Galactose medium

**Fig 9:**
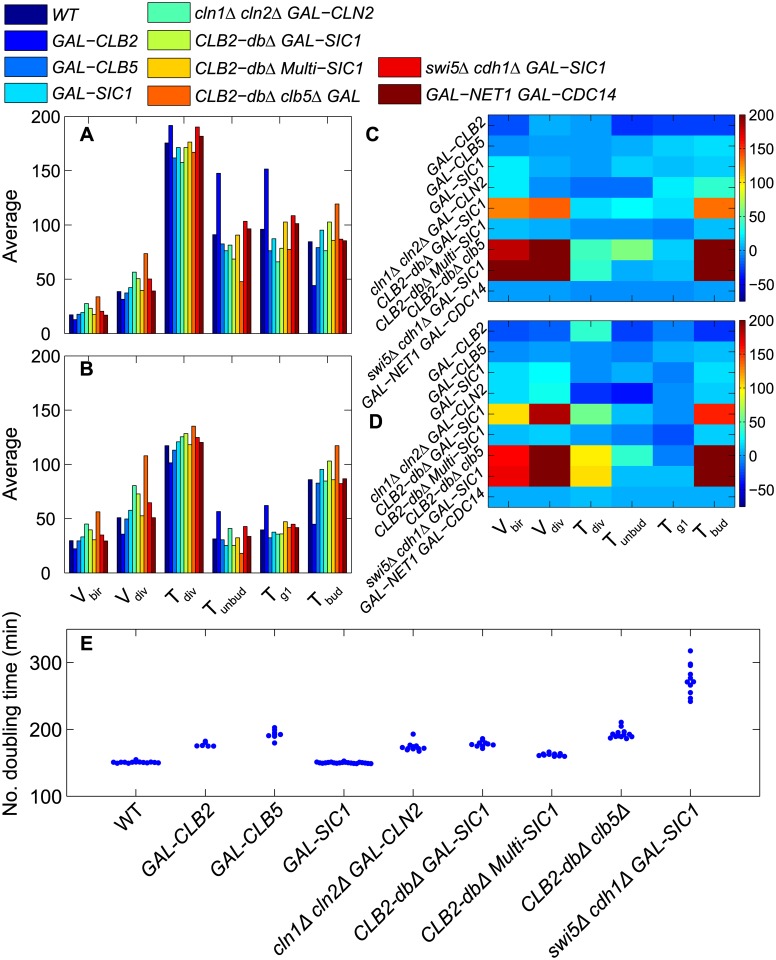
Mutant phenotypes in galactose medium. Same as Fig 8, but for computational cell cultures growing in galactose medium (μ = 0.00467 min^−1^).

Next we present stochastic simulations for mutant cells growing in galactose medium (Fig 9. Average cell size, cell cycle time and all other properties are quite different from one mutant to another. Over-expression of *CLB2* (*GAL-CLB2*) leads to an elongation of G1 phase because high Clb2 delays synthesis of G1/S cyclins by phosphorylating SBF, whereas the duration of budded phase shortens due to the rapid activation of mitotic phase by an abundance of Clb2. *SIC1* over-expression results in elongation of SG2M phase and cells that are larger than WT. *CLB2-dbΔ* [41] and *swi5Δ cdh1Δ* [42] strains, which are known to arrest in mitosis, are rescued by over-expression of *SIC1 (CLB2-dbΔ GAL-SIC1*, *CLB2-dbΔ Multi-SIC1* and *swi5Δ cdh1Δ GAL-SIC1*), as observed by [42, 43]. Over-expression of *NET1* [44] or *CDC14* [45] is lethal, but overexpression of *NET1* and *CDC14* together is not [44].

An interesting example of a growth-dependent mutant is *CLB2-dbΔ* (Clb2 protein lacks the “destruction box” sequence required for Cdc20-mediated degradation). This mutant strain, grown in either glucose or galactose, arrests in mitosis. But *CLB2-dbΔ clb5Δ* cells are partially viable in galactose medium (but not in glucose medium) [43]. Our stochastic model is consistent with these observations (Fig 9E) and with recent experimental observations [17] that these mutant cells show significant variability in number doubling time (Fig 9E) and cell cycle time (**Table I** in S1 Text) (see also **Figure N** in S1 Text).

*CLB2-dbΔ* cells arrest in mitosis due to very high activity of Clb2-dependent kinase, which shuts down the activation of Cdh1 and Sic1, which are required for mitotic exit. Deletion of *CLB5* from *CLB2-dbΔ* cells helps them to activate Sic1 and Cdh1, thereby promoting mitotic exit in poor growth medium such as galactose or raffinose. The rescue is possible in poor growth medium because, in our model, the synthesis of Clb2 depends on the specific growth rate (μ) of cells. Therefore, in galactose medium compared to glucose, there is less accumulation of Clb2 in M phase, allowing Sic1 and Cdh1 to drive some cells (stochastically) out of M phase and back to G1.

As expected, the model predicts that over-expression mutants *GAL-CLB2*, *GAL-CLB5*, *GAL-SIC1* and *GAL-NET1 GAL-CDC14* generally keep cell-to-cell variability at the same or slightly lower level than WT cells in galactose medium due to the increased protein expression. Mitotic arrest mutants rescued by over-expression of *SIC1* (*CLB2dbΔ GAL-SIC1* and *swi5Δ cdh1Δ GAL-SIC1*) and the growth-dependent mutant (*CLB2-dbΔ clb5Δ*) in galactose medium are highly noisy. Compared to WT cells in galactose medium, all other mutant cells proliferate slowly, except *GAL-SIC1* (Fig 9E). Among all the mutant cells that we investigated, the *swi5Δ cdh1Δ GAL-SIC1* strain proliferates the slowest because 63% of these cells are arrested in various phases of cell cycle: 40% unbudded due to increases Clb2 level, 22% budded with single copy of DNA, and 1% in M phase with high Clb2. For a similar reason, *GAL-CLB2* cells proliferate slowly in galactose medium, as do *CLB2-dbΔ* cells rescued either by over-expression of *SIC1* or by introducing *clb5Δ*. Surprisingly *GAL-CLB5* cells also proliferate slowly, and we find that ~19% of these cells arrest without buds because Clb2 level rises prematurely because of early activation of Fkh2 by Clb5.

Similar to glucose medium, we find that, for *cdh1Δ* cells simulated in galactose medium, the average number-doubling time (440 min) is much longer than for WT cells in the same medium. The increased number-doubling time is a consequence of the large fraction of cells (58%) that arrest at various phases of the cell cycle, primarily in G1 phase, due to abnormally high levels of Clb2 in *cdh1Δ* cells. The average duration of the cell division cycle also increases (**Table I** in S1 Text) due an elongated G1 phase. The variability of SG2M phase increases by several fold, causing increased noise in cycle time and cell sizes at birth and division.

### Cell cycle variability is driven by variability of the budded phase

**Fig 10:**
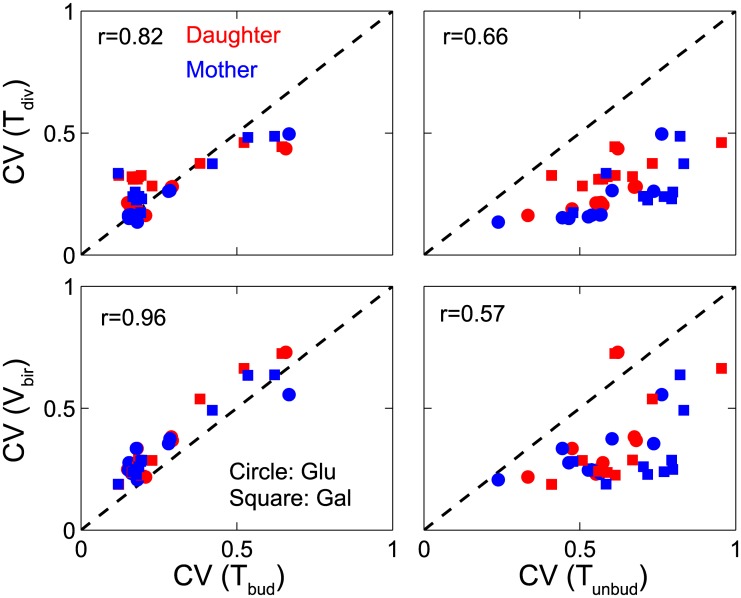
Variability in cell cycle time, and in cell size at birth, is closely correlated to variability in the duration of budded phase. The coefficients of variation of *T*_div_ (top row) and *V*_bir_ (bottom row) are correlated with the coefficients of variation in *T*_bud_ (left column) and *T*_unbud_ (right column) for simulated populations of viable mutant strains (including wild-type) in glucose and galactose media. The computed correlation coefficients are given on each plot.

Is the variability in cycle time and birth size a cumulative effect of variability of all phases of the cell cycle or is it dictated by the variability of a particular phase of cell cycle? Since G1 is the most variable phase of the budding yeast cell cycle, we might expect that variability in G1 duration (or duration of the unbudded phase of the cycle, which is easier to determine experimentally) dictates the variability in interdivision times and, hence, in cell sizes at birth. To address this question, we tabulated the coefficients of variation for birth size (*V*_bir_), cycle time (*T*_div_), and the durations of the unbudded (*T*_unbud_) and budded (*T*_bud_) phases of the cell cycle for both mother and daughter cells of the wild-type and all the viable mutant strains in Figs 8 and 9. Then we computed product-moment correlation coefficients (*r*) of the CVs of *V*_bir_ and *T*_div_ with respect to *T*_unbud_ and *T*_bud_ (Fig 10). Our results show, surprisingly, that the CVs of birth size and cycle time are more strongly correlated with the less variable phase of the cell cycle (the budded phase; *r* > 0.82) than they are with the more variable phase (the unbudded phase; *r* < 0.66).

To gain some insights into these results, consider the effects of size control operating at the Start transition of the budding yeast cell cycle. Start size control creates a negative correlation between cell size at birth and the duration of the unbudded phase of the cell cycle (see Fig 3). Hence, the size control mechanism tends to reduce the variability of cell size at Start among a population of cells. Consequently, most of the variability of cell size at division tends to accumulate in the budded phase of the cell cycle and, therefore, should correlate strongly with variability of time spent in the budded phase. This conclusion is confirmed in Fig 10 by the strong positive correlation between the coefficients of variation of *V*_bir_ and *T*_bud_ (remembering that CV(*V*_bir_) = CV(*V*_div_), if *V*_bir_ = *V*_div_/2; with strict equality upset somewhat by the asymmetric division process).

Less readily explained is the strong correlation of CV(*T*_div_) with CV(*T*_bud_) rather than with CV(*T*_unbud_). We might account for these trends with the following estimates. Since *T*_div_ = *T*_unbud_ + *T*_bud_, the expected values of these random variables are E(*T*_div_) = E(*T*_unbud_) + E(*T*_bud_), and, furthermore, Var(*T*_div_) = Var(*T*_unbud_) + Var(*T*_bud_), because *T*_bud_ and *T*_unbud_ are uncorrelated. If Var(*T*_bud_) ≈ Var(*T*_unbud_) and E(*T*_bud_) ≈ 2∙E(*T*_unbud_), as seem to be roughly true for budding yeast cells, then CV(*T*_div_) ≈ CV(*T*_bud_) and CV(*T*_div_) ≈ 0.5∙CV(*T*_unbud_), which align with our results in Fig 10.

### Other sources of noise

In addition to intrinsic noise, originating from fluctuations of molecular species, we also investigated the role of extrinsic noise on cell cycle properties. We introduced extrinsic noise into the model by partitioning molecular species at cell division according to a binomial random process, where the mean number of molecules of species X given to the daughter cell is $f∙N_{X}^{div}$, where $N_{X}^{div}$ is the total number of molecules of species X at the time of cell division and *f* is fraction of volume that goes to daughter cell, a deterministic factor. We find that binomial partitioning of molecular species causes no significant alteration in cell cycle variability (**Table J** in S1 Text).

We also explored the role of fluctuations in the division process by introducing Gaussian noise in the fraction, *f*. For example, in glucose medium, if *f* is chosen from a Gaussian distribution with mean 0.4 and CV = 5% or 10%, we observe only a small increase in variability of cell cycle properties (**Table J** in S1 Text). The greatest change is seen in the variability of cell size at birth, which is to be expected due to fluctuations in the division fraction.

## Discussion

Progression through the eukaryotic cell cycle is controlled by a complex network of interacting genes and proteins that ensures the proper sequencing of events and deals with problems (e.g., incomplete DNA replication or misaligned chromosomes) as they arise. This molecular control system must function with great fidelity, because mistakes are often fatal to the cell or its progeny. The greatest challenge to the fidelity of DNA replication and cell division in small eukaryotic cells is the limited number of molecules available to implement the control system. In a yeast cell (~50 fL volume), there are 1000–10,000 copies of each protein comprising the control system and 5–10 copies of each mRNA species encoding each of these proteins. The randomness of chemical reactions among such small numbers of molecules may lead to potentially large fluctuations in the numbers of protein molecules in the cell. It is a major scientific challenge to understand how the cell cycle control system can function correctly and robustly in the context of random molecular noise disrupting the interactions of the regulatory proteins.

One way to address this question is to compare the variability of cell cycle progression among individual cells for which all components of the control system are intact, i.e., wild-type (WT) cells, with the variability of mutant strains where certain cell-cycle genes are knocked-out or overexpressed; as has been done for budding yeast cells by [14, 16, 36]. Such comparisons can indicate which interactions in the network are most important for “noise abatement” because, when they are perturbed, cell cycle progression becomes noisier and less reliable.

As informative as these experimental studies have been, they need to be complemented by a comprehensive and accurate mathematical model of stochastic fluctuations in the cell cycle control system. Such a model is needed (i) to tie all the experiments together into a unified, self-consistent understanding of the effects of molecular noise in individual cells, (ii) to connect quantitative experimental measurements to a solid underlying theory of fluctuations in gene expression and protein function, (iii) to make interesting and (sometimes) unexpected predictions about cell cycle statistics in novel situations and/or mutant strains, and (iv) to provide a validated starting point for further theoretical studies of the surveillance mechanisms and checkpoints that ensure genomic stability from one generation to the next.

To build an accurate stochastic model of cell cycle control in budding yeast, we must know roughly how many molecules of mRNAs and proteins are encoded by each gene in the model. Protein abundances have been measured by several groups [29, 46–48]. We have used the numbers first reported by [29]. For mRNA abundances of cell cycle genes in single yeast cells, we have used recent measurements by our group [27, 49] using methods pioneered by [27, 49]

In a former paper [6], see also [50], we built a stochastic model of a simplified mechanism for cell cycle progression in budding yeast. The model was sufficient for analyzing the statistical characteristics of WT cells, and it was in good quantitative agreement with detailed observations of physiological and molecular events at the Start transition [16]. However, the model did not have sufficient molecular detail to address the statistical characteristics of mutant strains of budding yeast. We have corrected this deficiency here by expanding our earlier model to include G1 cyclins (Cln1 and Cln2), S-phase cyclins (Clb5 and Clb6), cyclin-dependent kinase inhibitors (Sic1 and Cdc6), and the anaphase promoting complex (APC:Cdc20), along with transcription factors (Swi5 and Fkh2) that were ignored in the earlier model. We added these components to the model specifically to address the experimental observations of Skotheim et al [14] and Yang et al [36], and to make predictions on the phenotypes of yeast strains carrying mutations in the *CLN*, *CLB*, *SIC1*, *SWI5* and *CDH1* genes. Although the present model is sufficient for these purposes, it still has gaps. In particular, its representation of “exit from mitosis” will need to be elaborated in a later version of the model, when we specifically study mutations in the spindle-assembly checkpoint and the mitotic exit network.

The first requirement for any theoretical model of a molecular mechanism in a living cell is that cell behaviors predicted by the model are consistent with the available experimental evidence. Achieving such consistency is not a trivial matter for a model that provides a comprehensive account of experiments gathered from many publications from a variety of laboratories. It is often done in two steps. First, model predictions are compared informally to experimental results to see if the model agrees qualitatively with observations and to get rough estimates of the parameter values in the model. Then the parameter values can be determined more precisely by statistical tests to achieve good quantitative agreement between simulations and experiments.

To this end we have taken some pains in this paper (Figs 3 and 4) to show that our model is in close agreement with the observed variability of WT cells growing in glucose, in galactose, and in glycerol-ethanol (richer-to-poorer carbon sources) (data from Di Talia et al [7]), and with the mRNA distributions in single cells reported by ourselves in an earlier publication (Ball et al, 2013, Gandhi et al [28]). Next, we have simulated many of the experiments in Skotheim et al [14], showing that our model successfully captures the crucial role played by the positive feedback loop (Cln1,2 ─┤Whi5 ─┤SBF → Cln1,2) in a rapid and coherent Start transition (Fig 6). Finally, we compared our model with the experiments in Yang et al [36] on Sic1 degradation (Fig 7). These experiments came to our attention after most of our calculations were completed, so, in a sense, they served as a test of the model. The model accounts qualitatively for the trends in their observations, but not in quantitative detail, because we had chosen a value for the rate constant of Sic1 degradation in WT cells that is too large by a factor of ~4. This discrepancy will be corrected in the next version of the model.

Once a model is shown to be consistent with most of the experimental data relevant to the scope of the model, then the model can be used to predict statistical properties of yeast cells under a variety of novel conditions. First, we used our model to predict the joint distributions of mRNA transcripts from all pairs of genes in the model (Fig 4B). Our subsequent measurements of some of these joint distributions appear qualitatively to be in good agreement with the predicted distributions (Fig 4C). A proper statistical analysis of these distributions will be required to compare them quantitatively. This will require multi-dimensional parameter optimization of the model in the context of all the relevant statistical data at our disposal. This analysis is presently underway.

We can also use the model to predict the properties of cells under conditions that are difficult to achieve experimentally. For example (case 1), in the model we can systematically decrease the strength of the Cln1,2 ─┤Whi5 ─┤SBF → Cln1,2 feedback loop (Fig 6C and 6D) and see how the activation times of the G1/S-regulon lose coherence. In a similar calculation (case 2), we showed how, as the strength of the Clb1,2 → Fkh2 → Clb1,2 feedback loop is reduced, the mean duration and (more so) the coefficient of variation of the SG2M phase of the yeast cell cycle increases (Fig 7D–7G). In case 1, the loss of coherence cannot be reversed by overexpression of *CLN3* (Fig 6C6). In case 2, the increased mean and CV can be reversed by increased expression of G1/S cyclins (Cln1,2,3 and Clb5,6).

The model makes many predictions about mutant phenotypes that are experimentally testable. We are particularly interested in mutant strains that are “partially viable,” i.e., cultures that proliferate more slowly than individual cells can grow under the prevailing nutritional conditions. Such discrepancies between a culture’s number doubling time (NDT) and its potential mass doubling time (MDT) arise when some cells become arrested in the cell cycle and fail to divide, although they continue to grow, at least for some time. In a previous paper [17], we have observed the growth and division of single cells of one such strain, *CLB2-dbΔ clb5Δ GAL-SIC1*, which expresses a stable form of Clb2 protein in the absence of Clb5 protein, and produces Sic1 (a Clb-kinase inhibitor) under control of a galactose-inducible promoter. These cells are inviable when grown in glucose medium (MDT = 100 min), becoming uniformly arrested in metaphase. In galactose medium (MDT = 150 min) they are viable, because they express enough Sic1 to neutralize the effects of excess Clb2. When grown in raffinose (MDT = 150 min; no excess Sic1), these cells are partially viable: they proliferate with NDT ≈ 200 min, and about one-third of the cells have failed to divide within 300 min after birth. We report our simulations of this strain in Fig 9 (we don’t need “*GAL-SIC1*” to keep the virtual strain “alive” in the computer): the cells that do divide have mean (*T*_div_) = 150 min = MDT, but the NDT of the simulated culture is 190 min, in line with our experimental observations [17].

Our model provides a molecular explanation for partial viability. Clb2-dependent kinase drives cells into mitosis and inhibits exit from mitosis. To progress from metaphase to anaphase to telophase, budding yeast cells must degrade the Clb-type cyclins that are present in metaphase cells. *CLB2-dbΔ* cells are inviable in glucose because they have accumulated too much Clb2 to exit from mitosis. When grown on poorer carbon sources (galactose or raffinose), cells are smaller at metaphase, so it is easier to clear the cell of Clb-type cyclins. Deleting *CLB5* from *CLB2-dbΔ* cells makes it easier for these cells to exit mitosis and increases the viability of the strain. Hence, *CLB2-dbΔ clb5Δ* cells, growing in galactose or raffinose, have ~67% chance of avoiding metaphase arrest and successfully dividing.

Our model predicts that many other mutant strains should show some degree of partial viability (see Fig 9E). Most notably, *swi5Δ cdh1Δ GAL-SIC1* cells (growing on galactose) double in number very slowly (NDT ≈ 280 min) because many cells are predicted to arrest in various phases of the cell cycle. In addition, *GAL-CLB5* cells (growing on galactose) are predicted to be partially viable (NDT ≈ 180 min) with 20% of the cells arresting without buds. Experimental tests of these predictions should provide important new clues about the roles of noise and feedback in robust cell cycle progression.

Fig 8E suggests that two other strains, growing on glucose, are especially prone to cell cycle arrest due to noise in the control system: *cdh1Δ* with NDT ≈ 200 min, and *cln1Δ cln2Δ sic1Δ* with NDT ≈ 140 min. The *cdh1Δ* strain is known to proliferate more slowly than WT cells [40], but only by 30%, not by two-fold, as predicted by our model. This discrepancy suggests that a closer examination of the *cdh1Δ* strain at the single-cell level will help to clarify the roles of Cdh1 in cell cycle progression.

A final prediction of the model is worth highlighting here. We find (Fig 10), unexpectedly, that overall variability in the cell cycle, i.e., the coefficients of variation for birth sizes and interdivision times, are strongly correlated to variability in the budded phase of the cell cycle rather than the unbudded phase of the cell cycle. This prediction also deserves close scrutiny experimentally.

### Conclusion

We have presented here a stochastic model of cell cycle progression in budding yeast that provides a quantitatively accurate account of the variabilities of cell cycle events that have been observed on single cells in a variety of laboratories. The model includes enough cell-cycle control genes to explain how the variabilities in these events change in mutant strains of yeast cells that are defective in several positive feedback loops of the control mechanism. Hence, the model can provide unprecedented insight into the molecular basis whereby feedback regulation minimizes cell-cycle variability in budding yeast cells. The model is based on elementary chemical reactions at the levels of gene expression (transcription and translation), protein modification (multi-site phosphorylation and dephosphorylation), and heteromeric protein complexes (association and dissociation). The expected numbers of mRNA and protein molecules in a single yeast cell in the model are derived from experimental measurements. The random events that characterize this macromolecular reaction system are simulated by Gillespie’s stochastic simulation algorithm [18], which provides the most reliable estimates of the effects of noise on chemical reaction systems. Hence, we believe that the present model provides an accurate account of the molecular basis of cell cycle variability in yeast cells. We demonstrate that the model is consistent with a broad spectrum of experimental observations on wild-type and mutant cells, and we use the model to make a number of interesting predictions that are amenable to experimental testing. Finally, the model provides a validated starting point for more complete models yet to be developed of the roles of feedback mechanisms in minimizing the deleterious effects of noise in cell cycle checkpoints, such as the DNA-damage and spindle-assembly checkpoints.

## Materials and Methods

### Budding yeast cell cycle

Budding yeast cells (*Saccharomyces cerevisiae*) divide unequally to produce a large mother cell and a small daughter cell. Shortly after birth the mother cell forms a bud that subsequently grows, and, around the same time, the mother cell starts replicating its DNA. These events are called Start of the budding yeast cell cycle. After DNA replication is complete, the nucleus migrates to the bud neck, where a mitotic spindle forms and the replicated chromosomes are partitioned evenly between the mother cell and the bud. Cytokinesis occurs at the bud neck separating the small daughter cell from the larger mother cell. The daughter cell, being small, must grow to a certain critical size before committing to the Start transition [51]. Therefore, on average, daughter cells have a longer unbudded phase (G1 phase) compared to mother cells. On the other hand, both mother and daughter cells spend about the same amount of time in S/G2/M phase. Hence, daughter cells have a longer cell cycle time, on average, than mother cells, and the average cell cycle time of a population of mother and daughter cells is close to the mass-doubling time of the population.

The central regulator of the cell cycle in budding yeast is a cyclin-dependent protein kinase (CDK), which is called Cdc28 in budding yeast (encoded by the *CDC28* gene). To be active as a kinase, Cdc28 must bind to a cyclin partner (there are nine cyclin genes in budding yeast: *CLN1-3* and *CLB1-6*), which also directs Cdc28 activity to specific protein targets. The timely phosphorylation of these target proteins drives the cell through the events of the cell cycle in the proper order. Unlike Cdc28 subunits, which are present at a constant total concentration throughout the cell cycle, cyclin subunits are temporally regulated. For example, Cln1-3 are abundant in G1 phase, Clb3-6 in S phase, and in Clb1-2 in G2/M phase [52–55]. The periodic appearance and disappearance of these cyclin proteins maintain the directionality of cell cycle progression in budding yeast.

In early G1 phase, the only available cyclin is Cln3, which is present at a low level. The synthesis of Cln3 seems to be linked to cell size, so that, as the cell grows, the accumulation of Cln3 (in association with Cdc28) initiates the earliest event of Start [56], which is the activation of the transcription factors SBF and MBF. Together these transcription factors regulate the expression of nearly 200 different genes [57], known as the G1/S regulon. But in early G1, the Whi5 protein silences these transcription factors [58, 59] [60] [19]. As the G1 cell grows in size, Cln3-dependent kinase (the Cln3:Cdc28 heterodimer) begins to phosphorylate and inactivate Whi5, which has 12 CDK consensus phosphorylation sites [58]. Phosphorylated Whi5 releases SBF, which promotes the expression of many G1/S proteins, including Cln1,2 and Clb3-6. Cln1,2 proteins (in association with Cdc28) accelerate their own production by phosphorylating Whi5 multiple times. Notice that Cln1,2, Whi5 and SBF are involved in a positive feedback loop (PFL): Cln1,2 –┤Whi5 –┤SBF → Cln1,2 [14, 61, 62].

As Cln1,2 proteins accumulate, they are primarily responsible for bud emergence and also activation of the B-type cyclins, Clb5,6. Expression of *CLB5*,*6* genes is upregulated by SBF and MBF, but Clb5,6-dependent kinase is inhibited by a stoichiometric binding partner, Sic1 [39, 63]. Cln1,2-dependent kinase phosphorylates Sic1, which has 9 CDK consensus phosphorylation sites. Six or more phosphorylations of Sic1 subject it to SCF-mediated ubiquitination and subsequent degradation by proteasomes [64, 65]. As Sic1 is degraded and Clb5,6:Cdc28 dimers are released from its inhibition, active Clb5,6-kinase also contributes to Sic1 phosphorylation. The PFL (Clb5,6 –┤Sic1 –┤Clb5,6) leads to rapid activation of Clb5,6-kinases, which are primarily responsible for initiation of DNA replication [66].

Mitotic cyclins Clb1,2, which drive the cell into M phase (mitosis), are kept low in G1/S phase by three processes. (1) Transcription of the *CLB1*,*2* genes is regulated by the forkhead (Fkh) family of transcription factors [67], which are unphosphorylated in G1/S and hence do not associate with their binding partner Mcm1 and coactivator Ndd1 [68, 69]. (2) Any Clb1,2 protein that may synthesized by leaky transcription of the repressed genes is rapidly ubiquitinated by the anaphase promoting complex (APC) in conjugation with Cdh1, and subsequently degraded by proteasomes [70, 71]. (3) In addition, G1 cells have an abundant supply of Sic1, which binds to and inhibits Clb1,2:Cdc28 heterodimers. All three inhibitory processes are disengaged by PFL’s. Clb1,2-dependent kinase phosphorylates (1) Fkh2, thereby activating the expression of *CLB1*,*2*, (2) Cdh1, thereby inactivating the ubiquitin-ligase activity of APC:Cdh1, and (3) Sic1, thereby promoting degradation of this Clb-inhibitor. In addition, Clb1,2-dependent kinase phosphorylates and inactivates Swi5, the transcription factor promoting expression of the *SIC1* gene. Cdh1 has 11 CDK consensus phosphorylation sites [72].

The combined effects of these PFL’s lead to a rapid rise of Clb1,2-activity in S/G2 phase of the cell cycle. Clb1,2-kinases shut down transcription of G1/S cyclins by phosphorylating and inactivating SBF [60, 68]. Clb1,2 activity also drives mitotic events of the cell cycle, such as chromosome condensation, spindle-pole body duplication and spindle assembly. At the same time, forkhead transcription factors drive synthesis of Cdc20, a binding partner of the APC, but the APC:Cdc20 complex is kept inactive by the mitotic checkpoint complex (MCC) while the replicated chromosomes are being aligned on the mitotic spindle.

When all chromosomes are properly aligned, the MCC is inactivated and active APC:Cdc20 drives the cell into anaphase by ubiquitinating two classes of proteins, securin and Clb1,2, which are then rapidly degraded by proteasomes. Securin degradation releases separase, a protease that destroys cohesin rings that have been holding sister chromatids together in metaphase [73]. Cohesin removal allows sister chromatids to be separated to opposite poles of the mitotic spindle [74, 75]. The degradation of Clb1,2 assures that the MCC is not reactivated during anaphase, but subsequent progression to telophase and cell division requires further degradation of Clb1,2 by APC:Cdh1. Re-activation of Cdh1 requires a phosphatase, Cdc14, that dephosphorylates most of the proteins previously phosphorylated by CDK [45].

For most of the cell cycle, Cdc14 is kept inactive by binding to Net1 in the nucleolus, in a complex known as regulator of nucleolar silencing and telophase (RENT) [44, 76]. Partial early release of Cdc14 occurs by the FEAR pathway (cdc-fourteen early anaphase release), whereby Clb2-dependent kinase and Cdc5 (polo-like kinase) phosphorylates Net1 multiple times releasing Cdc14 partially. The full release of Cdc14 needs activation of the mitotic exit network (MEN) pathway [77–79]. Completely released Cdc14 in telophase activates Cdh1 which in cooperation with APC degrades Clb1,2 completely. As these cyclins disappear, Swi5 becomes active again and promotes expression of *SIC1*. Sic1 protein sequesters any remaining B-type cyclins. MEN activation triggers cell division, producing mother and daughter cells in G1 phase of the cell cycle.

The facts we have reviewed here are collected in a series of network diagrams (**Figures A-F** in S1 Text), on which we base the mathematical model described in the next section.

### Mathematical model

The model we explore in this paper is based on the molecular regulatory networks diagrammed in **Figures A-F** in S1 Text. The new model is an extended version of an earlier model [6], which was sufficient for describing some features of stochastic cell-cycle progression in wild-type cells, but was inadequate for understanding the peculiarities of various mutant strains of budding yeast. Nonetheless, the underlying dynamical basis of both models is the same: a bistable switch—created by a double-negative feedback loop—embedded in two negative feedback loops (as described in the main text). We point out some specific features of the extended model.

The dramatic, irreversible transitions at Start and Exit of the budding yeast cell cycle are driven by sudden changes in the activities of specific proteins associated with these transitions. These sudden changes are related to the “ultrasensitive” responses of the proteins to phosphorylation and dephosphorylation, and these ultrasensitive responses seem to derive from the fact that all of these proteins are targeted by cyclin-dependent kinases for phosphorylation on multiple Ser/Thr-Pro sites. Provided these phosphorylations are carried out in a distributive (rather than a processive) manner, the phosphorylation profile of the target protein can exhibit ultrasensitive response (more so if the sites are phosphorylated in an ordered fashion rather than randomly). The theory of ultrasensitivity via multiple phosphorylation is described in several publications, including [80–83]. The theory is based on mass-action rate laws for the phosphorylation and dephosphorylation reactions, so it is especially suited for stochastic simulation by Gillespie’s SSA [18].

We relied on multisite phosphorylation to create ultrasensitive switches for eight proteins in the model. In each case, we use *n* to denote the number of phosphorylation sites. For proteins that are inactivated by CDK-mediated phosphorylation, with *m* = number of phosphorylated sites for which the protein is still active (0 ≤ *m* < *n*), we have: Whi5 (*n* = 10, *m* = 2), SBF (*n* = 4, *m* = 0), Sic1 (*n* = 9, *m* = 5), Cdh1 (*n* = 11, *m* = 0), Net1 (*n* = 8, *m* = 3) and Swi5 (*n* = 3, *m* = 0). Two proteins in the model are activated by CDK-mediated phosphorylation. Fkh2 has two phosphorylation sites, and we assume that both sites must be phosphorylated for Fkh2 to be an active transcription factor. The APC is a multi-protein complex [84] that is activated by multiple phosphorylations. In the model we treat the APC as a single unit with eleven phosphorylation sites; and we assume that, as APC gets phosphorylated, it binds more strongly to Cdc20, by a factor *β*^*n*−*k*^, where *β* = 0.525, *n* = 11 and *k* is the number of sites on APC that are currently phosphorylated. All Cdc20:APCP_*k*_ complexes are assumed to have equal potency in ubiquitinating substrates and marking them for degradation. Because the concentration of active complexes builds up slowly as APC gets successively phosphorylated, there is a delay in degrading Cdc20:APC substrates, thus ensuring that cells do not exit mitosis prematurely.

The phosphorylations of these substrates by cyclin-dependent kinases are opposed by a trio of phosphatases in the model. Because of the crucial role played by Cdc14 phosphatase in exit from mitosis, its regulation by Net1 is most carefully reproduced in the model (**Figure E** in S1 Text). An unspecified phosphatase, called “Ht1”, is employed in this module to dephosphorylate Net1. We assume that Ht1 is degraded by Cdc20:APC, in order to accelerate the phosphorylation of Net1 and the release of Cdc14 at the metaphase-to-anaphase transition. Ht1 bears some resemblance to Cdc55:PP2A phosphatase, but the mechanisms of regulating Cdc55:PP2A activity in yeast cells are considerably more complex than our assumption here that Ht1 is degraded by Cdc20:APC [85, 86]. We will provide a better representation of Cdc55:PP2A regulation in a future version of the model. Finally, a generic phosphatase, called “Hbf”, is assumed to function throughout the model, in opposition to the CDK phosphorylation steps. We assume that Hbf is expressed constitutively and its activity is unregulated.

The Cdc20:APC substrates in our model are the B-type cyclins (Clb2 and Clb5) and the phosphatase (Ht1), that dephosphorylates Net1 thereby releasing Cdc14. The current version of the model does not include the mitotic checkpoint complex, the securin-separase motif, or the FEAR and MEN pathways because we do not intend here to study mutations in these aspects of exit from mitosis. We postpone modeling these control systems for a later version of the model.

We assume that phosphorylation and dephosphorylation of Whi5, SBF, Cdh1, Fkh2, Swi5 and Net1 occur by an ordered mechanism, whereas Sic1 phosphorylation and dephosphorylation occur by a disordered mechanism, consistent with experimental observation [64].

We include transcription and translation of all sixteen genes in the model. Seven of them (*CLN1*, *CLN2*, *CLB5*, *CLB2*, *SIC1*, *SWI5* and *CDC20*) are regulated by transcription factors whose activities are modulated by the network. The other nine (*CLN3*, *WHI5*, *SBF*, *FKH2*, *APC*, *NET1*, *CDH1*, *CDC14* and *HBF*) are assumed to be expressed constitutively. Except for Cln3, the total amount of protein encoded by each of these genes remains constant during the cell cycle. *CLN3* mRNA and Cln3 protein show modest oscillations during the cell cycle [87], which we ignore in the present version of the model.

The effects of CDK-mediated phosphorylation of target proteins are reversed by the phosphatase Cdc14. As Clb levels fall and Cdc14 activity rises during telophase, Sic1 level rises abruptly (because Swi5, the transcription factor for *SIC1*, becomes active, and Sic1 itself becomes resistant to SCF-mediated degradation), and Cdh1 is activated. Active Cdh1:APC leads to rapid degradation of Clb2 and Cdc20. In addition to the regulated phosphatase, Cdc14, there is an unregulated phosphatase, Hbf, that opposes CDK activities at many places in the model.

The mechanistic details in Fig 1 are converted directly into reaction rate equations in **Table B** in S1 Text. The first equation in this table describes exponential growth of cell volume (*V*) with a specific growth rate, *μ*. The mass doubling time for each cell is given by ln(2)/*μ*.

### Deterministic simulations

We entered all the reactions of the model in the Parameter Estimation Toolkit (PET) (http://mpf.biol.vt.edu/pet/), a software tool that generates the corresponding ordinary differential equations for all variables of the model. We ran deterministic simulations in the same toolkit (LSODAR integrator), using the parameter values given in **Table C** in S1 Text and initial conditions in **Table A** in S1 Text.

In order to score the events of bud emergence and the onset of DNA synthesis, we created two “indicator” functions, *bud*_*ss*_ and *dna*_*ss*_ (see **Table B** in S1 Text). We score a budding event when the value of *bud*_*ss*_ increases to 25 nM, and we score initiation of DNA synthesis when *dna*_*ss*_ increases to 30 nM. We assume that all G1 and S cyclins contribute to the budding event, but only B-type cyclins drive the initiation of DNA synthesis. Each cyclin has an associated “efficiency” whose values are chosen to reproduce the experimentally observed timing of budding and DNA synthesis in mother and daughter cells of wild-type and cyclin-deletion strains.

In deterministic simulations, the cell divides when Clb2 concentration, [Clb2], drops below 12.5 nM. Division is assumed to be symmetric, and the populations of all molecular species are equally partitioned to the two newborn cells. We did not bother to implement asymmetric division in the deterministic simulation in Fig 2A.

### Stochastic simulations

In the stochastic version of our model we simulate the temporal sequence of 482 elementary chemical reactions using Gillespie’s SSA in a custom-written code. Because the reactions are elementary, Gillespie’s algorithm accurately quantifies variability originating from fluctuations of molecular species.

In stochastic simulations we take into account the fact that budding yeast cells divide asymmetrically into larger mother and smaller daughter cells. If *f* = fraction of cell size that goes to the daughter cell at division, then (1−*f*):*f* is the mother-to-daughter cell-size ratio at birth. In glucose medium, this ratio is approximately 0.6:0.4 (Di Talia, private communication). Size asymmetry increases further in poor growth conditions [88]; the ratio is approximately 0.65:0.35 in glycerol-ethanol medium (Di Talia, private communication). For galactose medium, we assume an intermediate ratio, 0.64:0.36. At division we partition the molecular species (proteins and mRNAs) into mother and daughter cells according to their size fraction, 1−*f* and *f*, respectively. For example, in glucose medium 40% of each molecular species goes into the daughter cell and the remainder goes into the mother. A different rule applies uniquely to Cln3 protein. Experimental observations [89] show that *CLN3* is differentially regulated in mother and daughter cells; in particular, newborn daughter cells contain ~3-fold less Cln3 compared to newborn mother cells, causing a daughter-specific delay of Start [15]. In our stochastic simulations, we partition 25% of Cln3 molecules at division to the daughter cell and 75% to the mother cell.

We start a simulation (at *t* = 0) with one cell at the initial conditions in **Table A** in S1 Text. The cell’s volume grows exponentially with time, and its CDK control system pushes it stochastically through the cell cycle. The cell divides eventually (say, at *t* = 101 min) when [Clb2] drops below 12.5 nM, as in the deterministic simulation. Applying our division rule, we create newborn mother and daughter cells and put them on a list, something like this: {(C1, BT1 = 101), (C2, BT2 = 101)}. We then take C1 from the top of the list and follow it from birth (at *t* = 101 min) to division (say, at *t* = 192 min). We then remove C1 from the list and place its progeny at the bottom of the list {(C2, BT2 = 101), (C3, BT3 = 192), (C4, BT4 = 192}. After each such operation, we take the cell from the top of the list (with the earliest birth time) to follow its cell division cycle, and place its progeny at the bottom, as before, always ordering cells with respect to birth time. In this way we create a ‘simulated culture’ of budding yeast cells. Because we have a full record of every cell-cycle event of every cell in the colony and we know all their pedigrees, we can compute any statistical attribute of the population of cells. For example, during the course of simulation we record the timing of bud emergence and initiation time of DNA synthesis by tracking *bud*_ss_ and *dna*_ss_, respectively. From these times (and the times when the cell was born and divided), we can calculate the durations of the cell cycle (*T*_div_), of the unbudded phase (*T*_unbud_), of the budded phase (*T*_bud_), of G1 phase (*T*_g1_), and of SG2M phase (*T*_sg2m_). Notice that *T*_div_ = *T*_unbud_ + *T*_bud_ and *T*_div_ = *T*_g1_ + *T*_sg2m_ but *T*_unbud_ ≠ *T*_g1_. We also know the size of every cell at each of these events.

Another event that we need to track (because it is easily measured in single-cell experiments with GFP-tagged Whi5) is the time when Whi5 leaves the nucleus. This event is thought to happen when Whi5 gets sufficiently phosphorylated by cyclin-dependent kinases. So we define the concentration of “nuclear” Whi5 as the total concentration of all Whi5-containing species with 0, 1 or 2 phosphate groups on Whi5 because these forms are capable of binding SBF. When this concentration drops below 200 nM, we say that Whi5 leaves the nucleus (i.e., GFP-staining of the nucleus is no longer evident).

Our stochastic simulation code also keeps track of cells that arrest in various phases of the cell cycle. If any of the “indicator” variables (*bud*_ss_ or *dna*_ss_ or [Clb2]) does not reach its threshold (for budding, DNA synthesis, or division, respectively) within a certain time window (typically 500 min), the stochastic code stops and the cell is recorded as arrested in the corresponding phase of the cell cycle.

### Measurement of joint distribution of mRNA

Measurements of mRNA abundance were performed on six strains in the Yeast-GFP Clone Collection developed by O’Shea and Weissman[90] which is now available from ThermoFisher Scientific. Specifically, we used the strains expressing Cln1-GFP, Clb2-GFP, Sic1-GFP, Cdc20-GFP, Whi5-GFP, and Net1-GFP. We measured the joint distribution of *CLN2* transcripts and the mRNA of these six genes by designing two sets of probes, one targeting the *CLN2* mRNA and one targeting the GFP domain fused to *CLN1, CLB2, SIC1, CDC20, WHI5*, and *NET1*.

#### FISH probes

Five 50mer oligonucleotides were designed to target the GFP domain and six 50mer oligonucleotides were designed to target *CLN2*. (**Table K** in S1 Text). All oligos were synthesized commercially (BioSearch Technologies). The oligos contained between three and five modified Ts that carried an amino group. A DyLight 550 NHS Ester labeling kit (Thermo Scientific # 62262) was used to conjugate the fluorescent dye DyLight 550 to the modified Ts in the GFP probes, while a DyLight 594 NHS Ester labeling kit (Thermo-Fisher # 46413) was used to attach DyLight 594 to the CLN2 probes.

#### Hybridization

Hybridization was performed as described in Zenklusen et al [49]. Briefly, cells were first fixed by incubation in 4% (v/v) paraformaldehyde (Electron Microscopy Sciences) for 45 min. After washing 3 times in Buffer B (1.2 M Sorbitol, 100 mM KHPO4, pH 7.5), cells were resuspended in spheroplast buffer (1.2 M Sorbitol, 100 mM KHPO4 pH 7.5), 20 mM Ribonucleoside-Vanadyl complex (New England Biolabs, S1402S), 20 mM β-mercaptoethanol, and 25 U lyticase (Sigma-Aldrich), and incubated at 30°C for 7 min to partially digest the cell walls. Cells were placed on poly-L-lysine (Sigma-Aldrich) coated coverslips and allowed to settle for 30 min at 4°C. The coverslips were washed once with Buffer B to remove unattached cells and stored overnight in 70% ethanol. The ethanol was removed, and the coverslips were incubated twice in 2× saline-sodium citrate (SSC) for 5 min and once in 40% formamide/2× SSC. Coverslips were then incubated with a mixture of the 5 probes (0.4 ng each, 2 ng total) overnight at 37°C. Unbound probe was removed by washing the coverslips twice with 40% formamide/2× SSC, once with 2× SSC/0.1% Triton, and once with 1× SSC. The coverslips were then washed in a Hoechst 33342 solution to stain nuclei and mounted on slides with Prolong Gold mounting medium (Life Technologies).

#### Image acquisition

All images were collected on an Axio Observer Z1 (Zeiss) microscope equipped with a CoolSNAP HQ2 CCD camera (Photometrics), a halogen lamp for bright field imaging, and a 120 W metal halide lamp for fluorescence excitation. The microscope is controlled by custom software developed in MATLAB that relies on the API of the open-source microscopy control software, μManager.62 The custom software performs image-processing tasks during acquisition and, among other features, allows for the automatic identification of suitable fields of view (FOVs); therefore, maximizing the number of cells that can be sampled. For each strain, 20 FOVs, containing ~1000 cells in total, were automatically selected from a user-defined area of the slide. A z-stack was collected at each FOV containing 31 focal planes separated by 0.2 μm, and 3 color channels: DyLight 550 (Chroma filter set SP102v1, exposure time: 2 s/plane), DyLight 594 (Chroma filter set SP103v2, exposure time 2 s/plane) and Hoechst 33342 (Chroma filter set 49000, exposure time: 150 ms/plane). In addition, a single phase-contrast image was acquired at the central plane for automated cell identification (see below).

#### Image processing

Phase-contrast images were segmented using custom software derived from Yeast Tree 1.6.3.15 The application relies on the MATLAB Image Processing toolbox. First, the function “imfill” was used to flood-fill local minima not connected to the image border, which fills in the center of the groups of cells. As each group of cells has slightly different levels to which the flood- fill will rise, we then searched the image histogram for intensities greater than the calculated background, taken from the border pixels, and with a frequency greater than the minimum cell area, generally set to 200 pixels. To keep only large groups of connected pixels, erosion (built-in function “imerode”) was performed, removing the outermost pixels of a region and eliminating small groups of pixels.

The next step is to separate these groups into individual cells. This was done with another call to “imerode” to cut the small necks that appear between touching cells. Once the cells are cut, the remaining connected regions were labeled with a call to the built-in function “bwlabel”, which identifies the individual cells and assigns each with a unique label. To finish, the cells were returned to their original sizes with a dilation (built-in function “imdilate”), which adds pixels around the edges of each cell.

Prior to the identification of spots, the 3-dimensional fluorescence z-stacks were reduced to 2D images by use of a maximum z-projection. Single spots, corresponding to mRNA molecules, were then identified using the algorithm described [91]. First, a local background subtraction, in which the mean of a 19 × 19 pixel neighborhood is subtracted from the central pixel, was used to highlight the bright punctate spots from the auto-fluorescence background of the cells. Then, pixels 5 standard deviations above the mean pixel value of the entire image were chosen as initial seeds for the spot detection routine. Gaussian weighting was then used to move each seed to the center of the 2D Gaussian intensity distribution. Spots with an integrated intensity of <350 are likely the result of non-specifically bound individual probes and were therefore removed. Finally, to avoid double counting a single mRNA, if the centers of 2 spots were within 2 pixels of each other, then the dimmer of the 2 spots was removed.

## Supporting Information

S1 Text Figure A: Cln1,2 module. Figure B: Clb5 module. Figure C: Clb2 module. Figure D: APC/Cdc20 module. Figure E: Cdc14 module. Figure F: Swi5 module. Figure G: Stochastic simulations of cell growth in poor growth media. Figure H: Joint distribution of μT_unbud_ and V_bir_ in mother cells. Figure I: Histograms of cell cycle properties in three growth media. Figure J: Temporal dynamics of *CLN2*, *RAD27*, *CLB2* gene activities. Figure K: Joint distributions of T_bir,*RAD27*_ and T_bir,*CLN2*_ in glucose medium. Figure L: Joint distributions of T_bir,*RAD27*_ and T_bir,*CLN2*_ in glycerol-ethanol medium. Figure M: Cumulative distributions of activation times of *CLN2* and *CLB2* genes. Figure N: Stochastic simulations of growth and division of *CLB2*-*dbΔ clb5Δ* cultures. Table A: List of all proteins and mRNAs with initial values used in the model. Table B: List of all the equations used in the deterministic version of the model. Table C: Parameter values used for simulations of wild-type cells. Table D: List of all the mutants simulated with the relevant modification of parameters. Table E: Average and coefficient of variation for cell cycle properties. Table F: Average duration of cell cycle phases in WT and mutants. Table G: Standard deviation of cell cycle phases in WT and mutants. Table H: Average and CV of cell cycle properties in glucose medium. Table I: Average and CV of cell cycle properties in galactose medium. Table J: Effects of external noise on CV of cell cycle properties. Table K: Oligonucleotides used to target GFP-tagged cell cycle genes and the *CLN2* gene.
(DOCX)

S1 Data Set Deterministic simulations for all the strains listed in the Table D in S1 Text. (DOCX)

S2 Data Set Two dimensional FISH data for mRNA. (XLS)

S1 Code File Deterministic model (PET file). (PET)

S2 Code File Stochastic model (FORTRAN file). (F)

S3 Code File Input file for stochastic model. (IN)
