# Correction: A Stochastic Model of the Yeast Cell Cycle Reveals Roles for Feedback Regulation in Limiting Cellular Variability

**DOI:** 10.1371/journal.pcbi.1005323

**Published:** 2017-01-12

**Authors:** The PLOS Computational Biology Staff

References 1, 2 and 3 are incomplete. The correct references should read as follows:

1. Forsburg SL, Nurse P. Cell Cycle Regulation in the Yeasts Saccharomyces cerevisiae and Schizosaccharomyces pombe. Annual Review of Cell Biology. 1991; 7(1):227–56. PMID: 1809348

2. Murray AW. Recycling the Cell Cycle: Cyclins Revisited. Cell. 2004; 116(2):221–34. PMID: 14744433

3. Jorgensen P, Tyers M. How Cells Coordinate Growth and Division. Current Biology. 2004; 14(23):R1014-27. doi:10.1016/j.cub.2004.11.027 PMID: 15589139

The publisher apologizes for this error.
