# Supplementary material for: A Stochastic Model of the Yeast Cell Cycle Reveals Roles for Feedback Regulation in Limiting Cellular Variability
Source: PLoS Comput Biol. 2016 Dec 9;12(12):e1005230. doi: 10.1371/journal.pcbi.1005230 (PMC5147779; doi:10.1371/journal.pcbi.1005230)
Supplement: S1 Text — Figure A: Cln1,2 module. Figure B: Clb5 module. Figure C: Clb2 module. Figure D: APC/Cdc20 module. Figure E: Cdc14 module. Figure F: Swi5 module. Figure G: Stochastic simulations of cell growth in poor growth media. Figure H: Joint distribution of μTunbud and Vbir in mother cells. Figure I: Histograms of cell cycle properties in three growth media. Figure J: Temporal dynamics of CLN2, RAD27, CLB2 gene activities. Figure K: Joint distributions of Tbir,RAD27 and Tbir,CLN2 in glucose medium. Figure L: Joint distributions of Tbir,RAD27 and Tbir,CLN2 in glycerol-ethanol medium. Figure M: Cumulative distributions of activation times of CLN2 and CLB2 genes. Figure N: Stochastic simulations of growth and division of CLB2-dbΔ clb5Δ cultures. Table A: List of all proteins and mRNAs with initial values used in the model. Table B: List of all the equations used in the deterministic version of the model. Table C: Parameter values used for simulations of wild-type cells. Table D: List of all the mutants simulated with the relevant modification of parameters. Table E: Average and coefficient of variation for cell cycle properties. Table F: Average duration of cell cycle phases in WT and mutants. Table G: Standard deviation of cell cycle phases in WT and mutants. Table H: Average and CV of cell cycle properties in glucose medium. Table I: Average and CV of cell cycle properties in galactose medium. Table J: Effects of external noise on CV of cell cycle properties. Table K: Oligonucleotides used to target GFP-tagged cell cycle genes and the CLN2 gene. (DOCX) [file pcbi.1005230.s001.docx]

**Supplementary Figures and Tables to:**

**A stochastic model of the yeast cell cycle reveals roles for feedback regulation in limiting cellular variability**

Debashis Barik,^1,*^ David A. Ball,^2,#^ Jean Peccoud^2,ⱡ^ and John J. Tyson^2,3,*^

^1^School of Chemistry, University of Hyderabad, Hyderabad, Telangana, 50046, India

^2^Virginia Bioinformatics Institute, Virginia Polytechnic Institute & State University, Blacksburg VA 24061 USA

^3^Department of Biological Sciences, Virginia Polytechnic Institute & State University, Blacksburg VA 24061 USA

^*^Corresponding authors. E-mail addresses: [tyson@vt.edu](mailto:tyson@vt.edu) and [dbariksc@uohyd.ac.in](mailto:dbariksc@uohyd.ac.in)

^#^Present address: Laboratory of Receptor Biology and Gene Expression, National Cancer Institute, Bethesda MD

^ⱡ^Present address: Department of Chemical & Biological Engineering, Colorado State University, Fort Collins CO 80523 USA

**Table of Contents**

| **Figures and Tables:** | **Page #** |
| --- | --- |
| Supplementary Figure A: Cln1,2 module | 4 |
| Supplementary Figure B: Clb5 module | 5 |
| Supplementary Figure C: Clb2 module | 6 |
| Supplementary Figure D: APC/Cdc20 module | 7 |
| Supplementary Figure E: Cdc14 module | 8 |
| Supplementary Figure F: Swi5 module | 9 |
| Supplementary Figure G: Stochastic simulations of cell growth in poor growth media | 10 |
| Supplementary Figure H: Joint distribution of μT_unbud_ and V_bir_ in mother cells | 10 |
| Supplementary Figure I: Histograms of cell cycle properties in three growth media | 11 |
| Supplementary Figure J: Temporal dynamics of *CLN2*, *RAD27*, *CLB2* gene activities | 12 |
| Supplementary Figure K: Joint distributions of T_bir,_*_RAD27_* and T_bir,_*_CLN2_* in glucose medium | 13 |
| Supplementary Figure L: Joint distributions of T_bir,_*_RAD27_* and T_bir,_*_CLN2_* in glycerol-ethanol medium | 14 |
| Supplementary Figure M: Cumulative distributions of activation times of *CLN2* and *CLB2* genes | 15 |
| Supplementary Figure N: Stochastic simulations of growth and division of *CLB2*-*dbΔ* *clb5Δ* cultures | 15 |
| Supplementary Table A: List of all proteins and mRNAs with initial values used in the model. | 16 |
| Supplementary Table B: List of all the equations used in the deterministic version of the model | 18 |
| Supplementary Table C: Parameter values used for simulations of wild-type cells | 24 |
| Supplementary Table D: List of all the mutants simulated with the relevant modification of parameters | 26 |
| Supplementary Table E: Average and coefficient of variation for cell cycle properties | 29 |
| Supplementary Table F: Average duration of cell cycle phases in WT and mutants | 30 |
| Supplementary Table G: Standard deviation of cell cycle phases in WT and mutants | 31 |
| Supplementary Table H: Average and CV of cell cycle properties in glucose medium | 32 |
| Supplementary Table I: Average and CV of cell cycle properties in galactose medium | 33 |
| Supplementary Table J: Effects of external noise on CV of cell cycle properties | 34 |
| Supplementary Table K: Oligonucleotides used to target GFP-tagged cell cycle genes and the *CLN2* gene. | 35 |
| References | 36 |


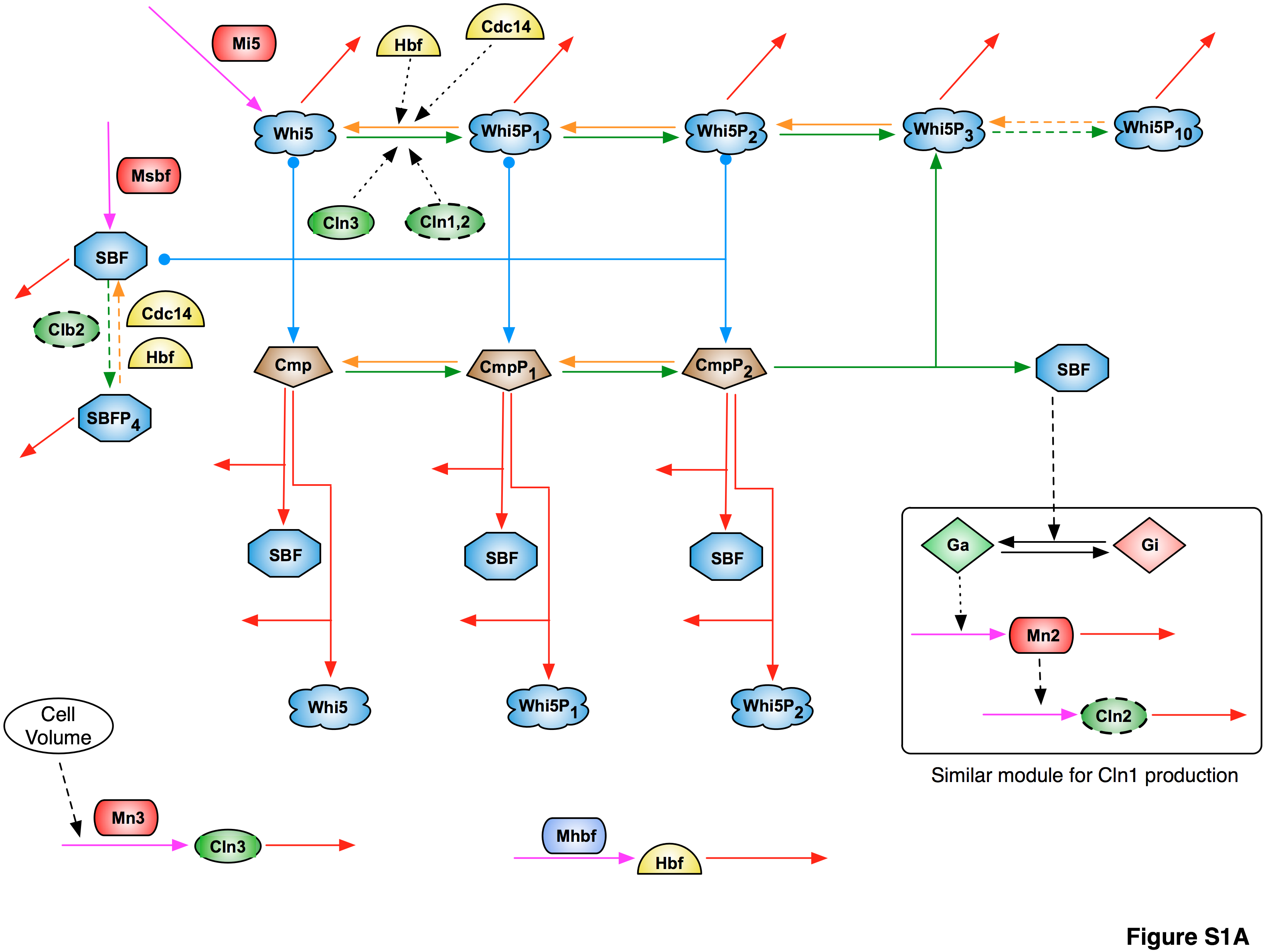


**Figure A:** Cln1,2 module. Expression of *CLN1* and *CLN2* genes (lower right) is controlled by the transcription factor, SBF, which is sequestered in an inactive complex (Cmp) by binding to Whi5. Phosphorylation of Whi5 by Cln-dependent kinases (top row) releases SBF from the inactive complex. Free SBF is inactivated by multi-site phosphorylation by Clb2-dependent kinase (far left). Phosphate groups are removed by an unregulated phosphatase, Hbf, and by a regulated phosphatase, Cdc14 (see panel E). Synthesis of Cln3 (lower left) is assumed to be hyper-responsive to cell growth, as a mechanism for “size control” in the model.


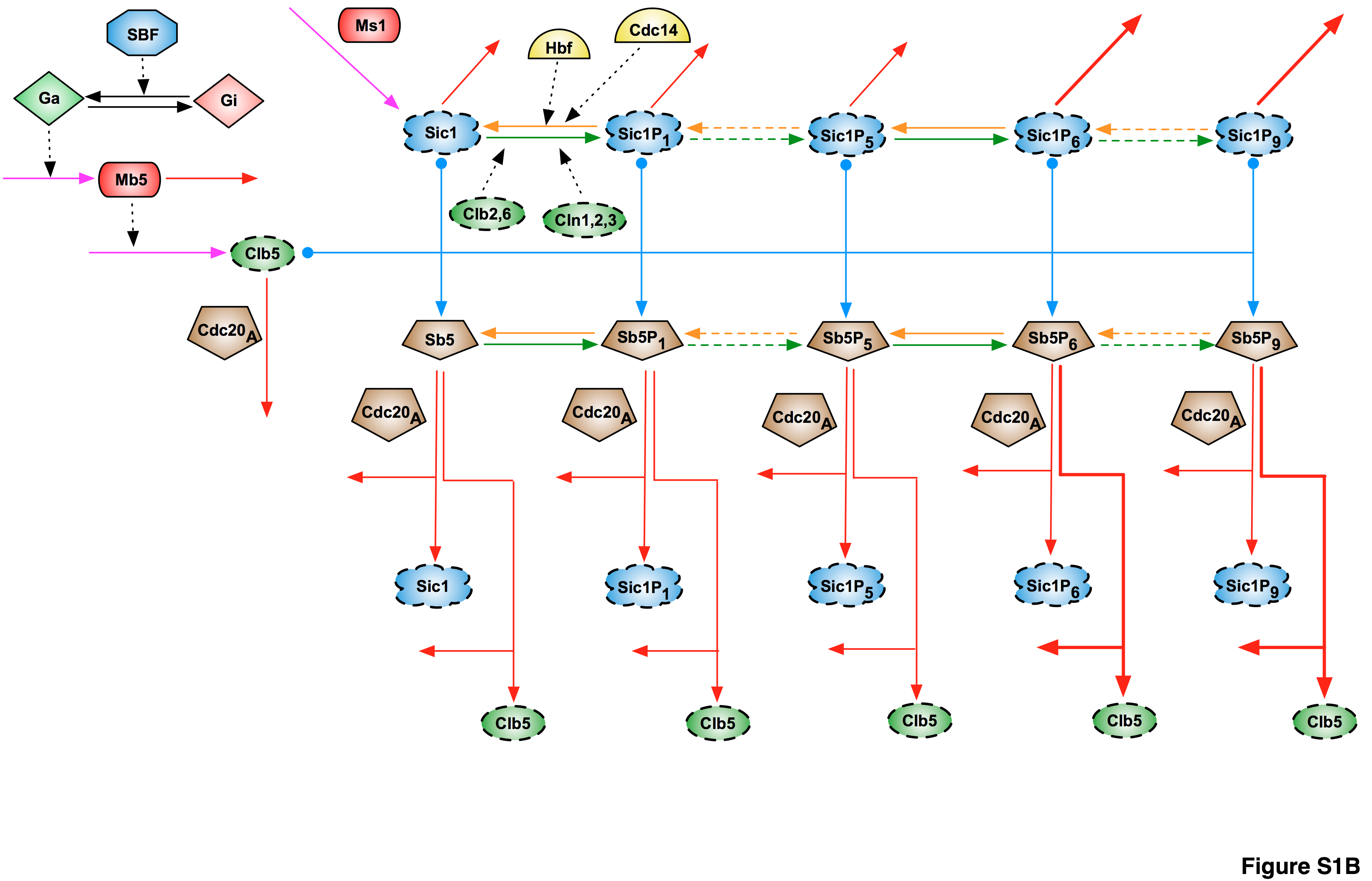


**Figure B:** Clb5 module. SBF activates expression of the *CLB5* gene (far left). Clb5-dependent kinase is kept inactive by binding to Sic1. Sic1 is phosphorylated on nine sites: the hypo-phosphorylated forms are very stable, whereas Sic1P_6_ … Sic1P_9_ are very unstable. Clb5 protein is degraded by a Cdc20:APC-dependent pathway.


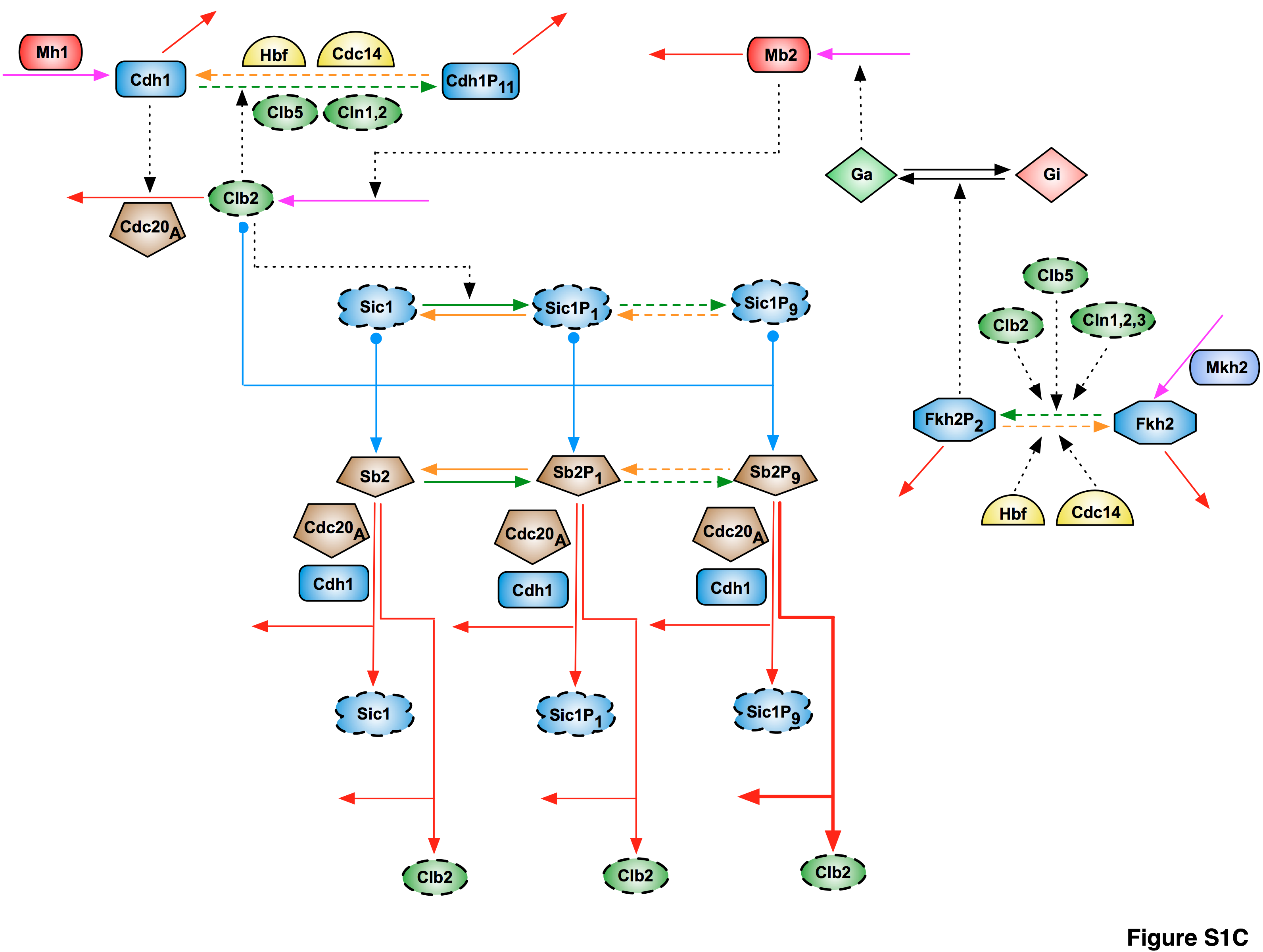


**Figure C:** Clb2 module. Expression of the *CLB2* gene is activated by the Fkh2 transcription factor, which must be doubly phosphorylated (far right) for its activation. Clb2 protein is degraded by Cdc20:APC- and Cdh1:APC-dependent pathways (upper left). Sic1 is a stoichiometric inhibitor of Clb2-dependent kinase.


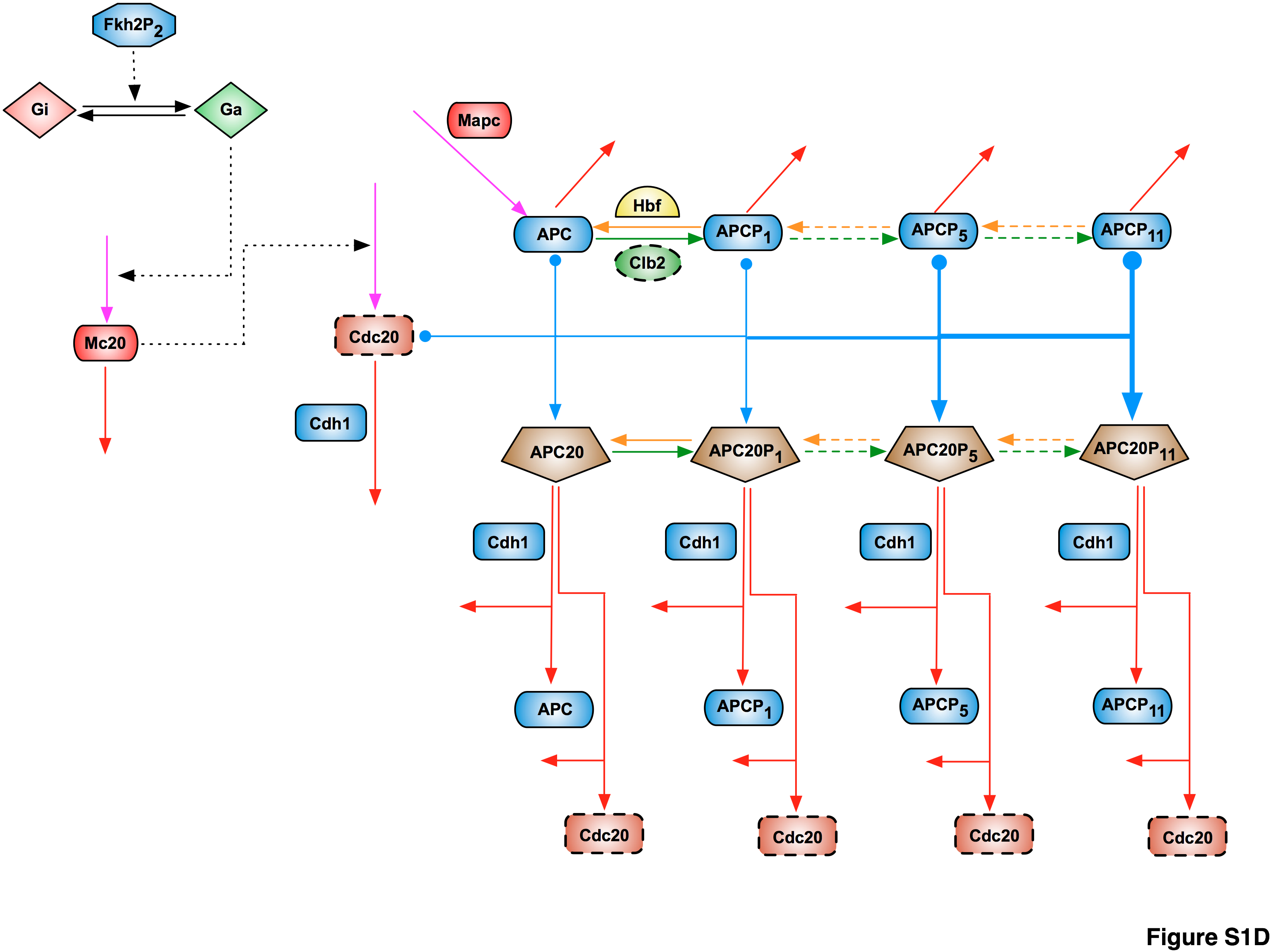


**Figure D:** APC/Cdc20 module. Fkh2 drives expression of the *CDC20* gene (upper left). Cdc20 protein, which is degraded by the Cdh1:APC pathway, binds to APC in a manner that is dependent on multi-site phosphorylation of the APC by Clb2-dependent kinase.


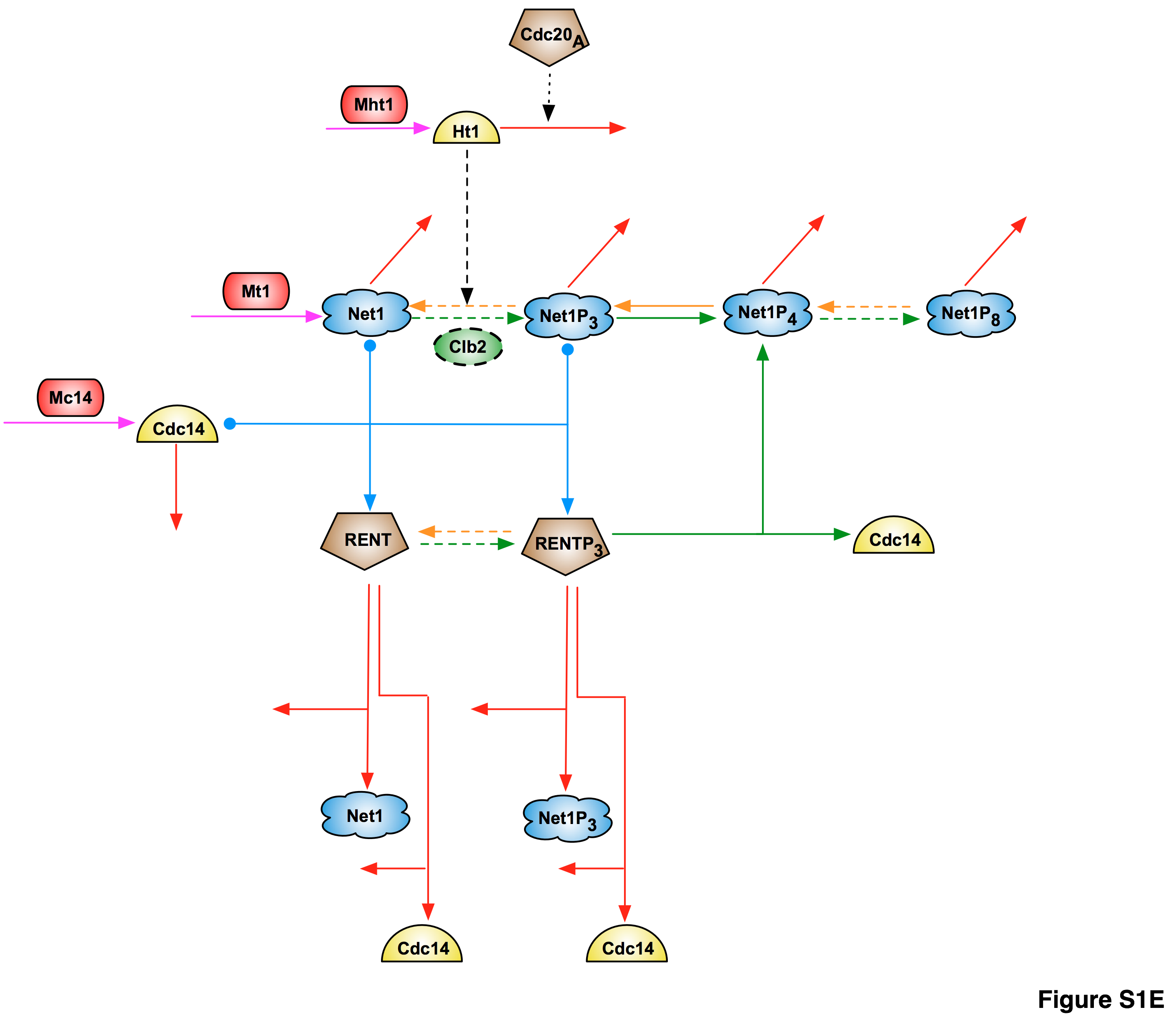


**Figure E:** Cdc14 module. Cdc14 phosphatase is kept inactive during most of the cell cycle by binding to Net1 to form the RENT complex (middle). Release of Cdc14 from the RENT complex is driven by multi-site phosphorylation of Net1 by Clb2-dependent kinase. Net1 is dephosphorylated by a phosphatase, Ht1, which is destroyed at the metaphase-to-anaphase transition by active Cdc20:APC (top).


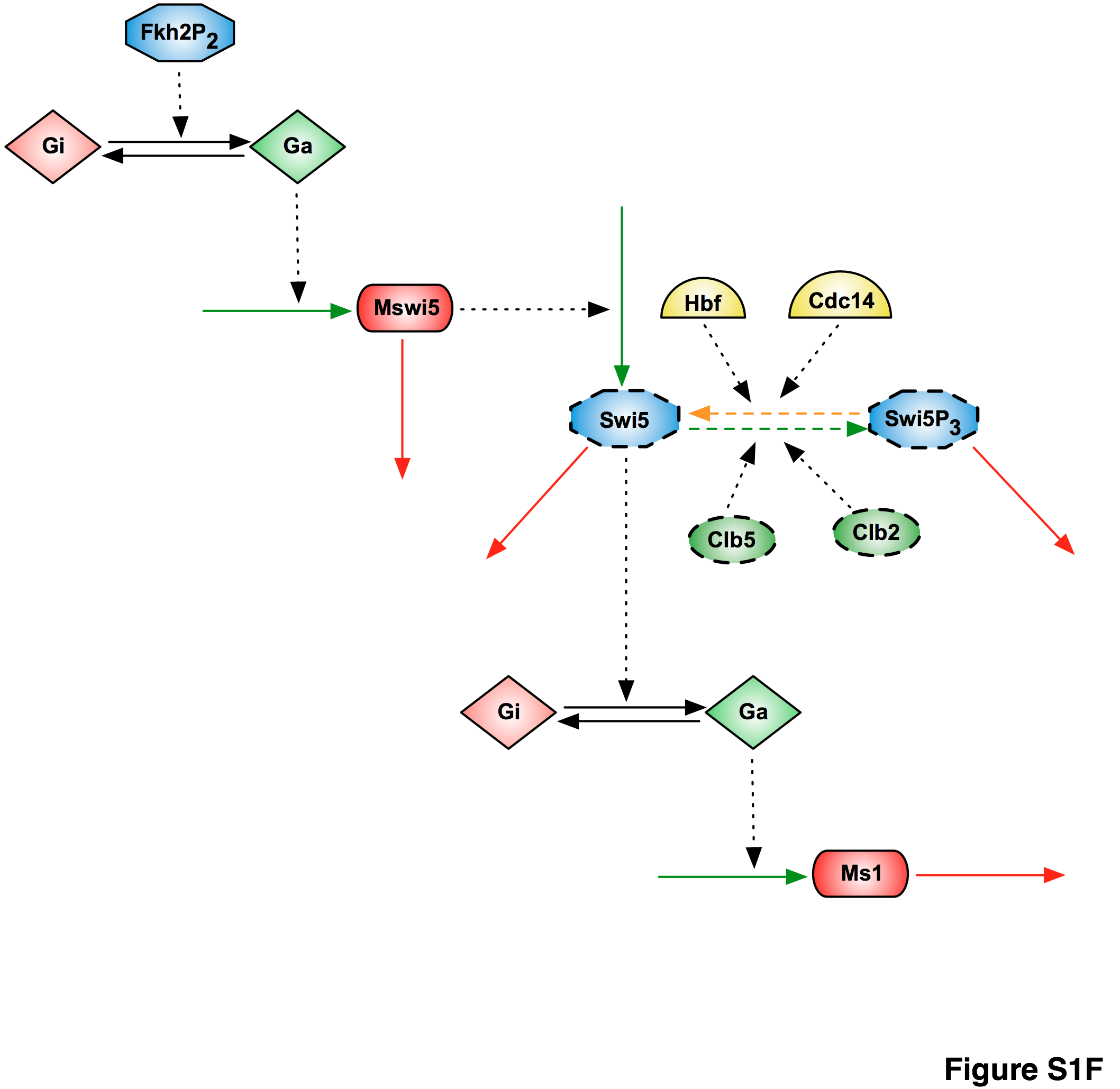


**Figure F:** Swi5 module. Swi5 is a transcription factor that activates expression of the *SIC1* gene (bottom). Swi5 is inactivated by multi-site phosphorylation by Clb5- and Clb2-dependent kinase activities, initially working against the generic phosphatase, Hbf. Swi5 is activated at anaphase, when Cdc14 is released from Net1 and the B-type cyclins are degraded by Cdc20:APC (middle). *SWI5* gene expression is turned on by active Fkh2 (top left).

**Figure G:** Stochastic simulations of the proliferation of wild-type (WT) cells growing in galactose medium (left panel; μ = 0.00467 min^−1^) and in glycerol-ethanol medium (right panel; μ = 0.00398 min^−1^). Inset: average doubling time of each simulated culture.

**Figure H:** Joint distribution of the duration of the unbudded phase and cell size at birth (normalized by the average size of cells at bud formation) for mother cells growing in glucose medium (μ = 0.007 min^−1^). A two-slope model fits the trends in this joint distribution.


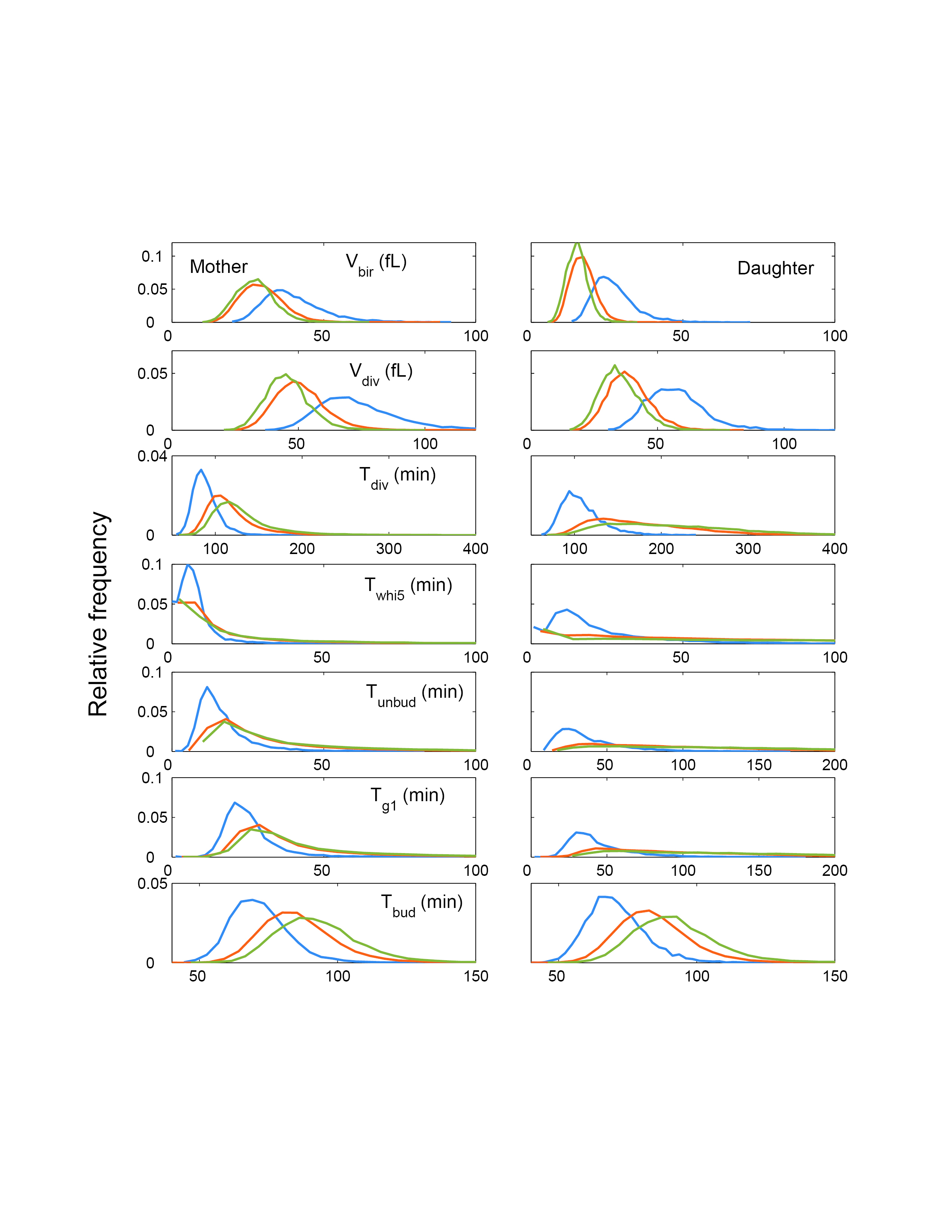


**Figure I:** Histograms of cell cycle properties computed from stochastic simulations with μ = 0.007 min^−1^ (glucose, blue), μ = 0.00467 min^−1^ (galactose, red) and μ = 0.00398 min^−1^ (glycerol-ethanol, green).

**Figure J:** Temporal dynamics of gene expression for representative cells selected from stochastic simulations of a cell culture growing in glucose medium (μ = 0.007 min^−1^). The genes are: *CLN2* (left column), *RAD27* (middle column) and *CLB2* (right column). Wild-type (WT) cells are rows 1 (daughter cells) and 3 (mother cells); *cln1Δ cln2Δ* cells are rows 2 (daughter cells) and 4 (mother cells). In *cln1Δ cln2Δ* cells, “*CLN2*” refers to expression of a reporter protein driven by the *CLN2* promoter.

**Figure K:** Joint distributions of T_bir,_*_RAD27_* to T_bir,_*_CLN2_* for *cln1Δ* cells (top panels) and *cln2Δ* cells (bottom panels) from stochastic simulations of cells growing in glucose medium (μ = 0.007 min^−1^). The correlations for mother and daughter cells are shown separately on the right side.

**Figure L:** Joint distributions of T_bir,_*_RAD27_* to T_bir,_*_CLN2_* for mother cells (blue rectangles) and daughter cells (red rectangles) from stochastic simulations of cell cultures growing in glycerol-ethanol medium (μ = 0.00398 min^−1^). Top left, WT cells; top right, *cln1Δ cln2Δ* cells; bottom left, *cln1Δ* cells; bottom right, *cln2Δ* cells.

**Figure M:** Cumulative distributions of the activation times of *CLN2* (solid lines) and *CLB2* (dashed lines) in WT cells (red curves) and *cln1Δ cln2Δ* cells (blue curves) from stochastic simulations of cell cultures growing in glucose medium (left panel, μ = 0.007 min^−1^) and in glycerol-ethanol medium (right panel, μ = 0.00398 min^−1^). Compare left panel to Figure 1f of Skotheim et al (Skotheim et al, 2008).

**Figure N:** Stochastic simulations of *CLB2-dbΔ clb5Δ* cells growing in galactose medium (μ = 0.00467 min^−1^). Top panels: increase in cell numbers for Individual runs (left), and average number-doubling times of the individual runs (right). Bottom panels: histograms of cell division times (left) and durations of budded phase (right) for mother cells (blue) and daughter cells (red).

**Supplementary Tables**

**Table A:** List of all proteins and mRNAs in the model, with initial values (numbers of molecules) used for simulations.

| **Variable** | **Description** | **Initial Value** |
| --- | --- | --- |
| **Proteins and complexes** | | |
| Cln3 | Heterodimer of Cln3 and Cdc28 proteins | 163 |
| Cln1 | Heterodimer of Cln1 and Cdc28 proteins | 77 |
| Cln2 | Heterodimer of Cln2 and Cdc28 proteins | 105 |
| Clb5 | Heterodimers of Cdc28 protein with both Clb5 and Clb6 | 46 |
| Clb2 | Heterodimers of Cdc28 protein with both Clb1 and Clb2 | 163 |
| Hbf | Constitutively active phosphatase that acts on all Cdc28 target proteins | 3136 |
| Ht1 | Phosphatase that dephosphorylates Net1 | 177 |
| Cdc14 | Cdc14 phosphatase | 496 |
| Whi5 | Whi5 protein; stoichiometric inhibitor of SBF | 4145 |
| Whi5P_1_-P_10_ | Phosphorylated forms of Whi5 protein | 1286, 400, 131, 39, 11, 3, 1, 0, 0, 0 |
| Cmp | Complex of Whi5 and SBF | 527 |
| CmpP_1_-P_2_ | Phosphorylated forms of complex Cmp | 157, 39 |
| SBF | Transcription factor for *CLN1,2* and *CLB5,6* | 5 |
| SBFP_1_-P_4_ | Phosphorylated forms of SBF protein | 103, 257, 493, 771 |
| Cdh1 | Cdh1 protein, a partner of APC | 977 |
| Cdh1P_1_-P_11_ | Phosphorylated forms of Cdh1 | 448, 293, 236, 207, 183, 158, 131, 102, 73, 46, 24 |
| Swi5 | Swi5 protein, a transcription factor for *SIC1* | 931 |
| Swi5P_1_-P_3_ | Phosphorylated forms of Swi5 | 24, 1, 0 |
| Sic1 | Sic1 protein, a stoichiometric inhibitor of B-type cyclins | 5 |
| Sic1P_1_-P_9_ | Phosphorylated forms of Sic1 protein | 6, 9, 11, 12, 10, 5, 2, 0, 0 |
| Sb2 | Complex of Sic1 and Clb2 | 2 |
| Sb2P_1_-P_9_ | Phosphorylated forms of Sb2 | 11, 30, 57, 73, 63, 36, 14, 3, 0 |
| Sb5 | Complex of Sic1 and Clb5 | 0 |
| Sb5P_1_-P_9_ | Phosphorylated forms of Sb5 | 0, 1, 2, 3, 2, 1, 0, 0, 0 |
| Fkh2 | Fork-head family transcription factor for *CLB2* | 1733 |
| Fkh2P_1_-P_2_ | Phosphorylated forms of Fkh2 | 140, 13 |
| Cdc20 | Cdc20 protein | 43 |
| APC | Anaphase Promoting Complex, an E3 ubiquitin ligase | 252 |
| APCP_1_-P_11_ | Phosphorylated forms of APC | 167, 152, 145, 138, 128, 116, 104, 93, 84, 73, 54 |
| APC20 | Complex of Cdc20 and APC | 0 |
| APC20P_1_-P_11_ | Phosphorylated forms of APC20 | 0, 0, 0, 0, 0, 0, 0, 0, 1, 1, 1 |
| Net1 | Net1 protein, a stoichiometric inhibitor of Cdc14 | 59 |
| Net1P_1_-P_8_ | Phosphorylated forms of Net1 | 15, 11, 39, 230, 316, 527, 1029, 1825 |
| RENT | Complex of Net1 and Cdc14 | 194 |
| RENTP_1_-P_3_ | Phosphorylated forms of RENT | 165, 161, 150 |
| **mRNAs** | | |
| Variable | Description |  |
| Mn1 | mRNA for *CLN1* | 1 |
| Mn2 | mRNA for *CLN2* | 1 |
| Mn3 | mRNA for *CLN3* | 4 |
| Mb5 | mRNA for *CLB5* | 1 |
| Mb2 | mRNA for *CLB2* | 3 |
| Ms1 | mRNA for *SIC1* | 6 |
| Mswi5 | mRNA for *SWI5* | 6 |
| Mc20 | mRNA for *CDC20* | 4 |
| Mh1 | mRNA for *CDH1* | 4 |
| Mi5 | mRNA for *WHI5* | 2 |
| Mt1 | mRNA for *NET1* | 4 |
| Mc14 | mRNA for *CDC14* | 4 |
| Mfkh2 | mRNA for *FKH2* | 4 |
| Mapc | mRNA for *APC* | 4 |
| Mbf | mRNA for *SBF* | 4 |
| Mhbf | mRNA for *HBF* | 4 |
| Mht1 | mRNA for *HT1* | 5 |
| **Genes** | | |
| G_a,n1_ | Active form of *CLN1* | 1 |
| G_a,n2_ | Active form of *CLN2* | 1 |
| G_a,b5_ | Active form of *CLB5* | 1 |
| G_a,b2_ | Active form of *CLB2* | 0 |
| G_a,s1_ | Active form of *SIC1* | 1 |
| G_a,swi5_ | Active form of *SWI5* | 0 |
| G_a,c20_ | Active form of *CDC20* | 0 |
| G_a,rad27_ | Active form of *RAD27* | 0 |
| V | Volume of cell | 26.6 (fL) |

**Table B:** List of all the equations used in the deterministic version of the model. *N*_X_ represents number of molecules of variable X.

| Cell growth | |
| --- | --- |
| 1 | $\frac{dV}{dt}=\mu\cdot V$ |
| Equations for cyclins | |
| 2 | $\frac{dN_{Cln3}}{dt}=k_{s,n3}\cdot V^{2}\cdot N_{Mn3}-\gamma_{n3}\cdot N_{Cln3}$ |
| 3. | $\frac{dN_{Cln1}}{dt}=k_{s,n1}\cdot V\cdot N_{Mn1}-\gamma_{n1}\cdot N_{Cln1}$ |
| 4. | $\frac{dN_{Cln2}}{dt}=k_{s,n2}\cdot V\cdot N_{Mn2}-\gamma_{n2}\cdot N_{Cln2}$ |
| 5. | $\frac{dN_{Clb5}}{dt}=k_{s,b5}\cdot V\cdot N_{Mb5}-\Gamma_{b5}\cdot N_{Clb5}-\frac{k_{a,b5}}{V}\cdot N_{Clb5}\cdot\sum_{i=0}^{9} N_{{Sic1P}_{i}}+k_{d,b5}\cdot\sum_{i=0}^{9} N_{{Sb5P}_{i}}$  $+\gamma_{s1}\cdot\sum_{i=0}^{5} N_{{Sb5P}_{i}}+\gamma_{s1}^{'}\cdot\sum_{i=6}^{9} N_{{Sb5P}_{i}}$ |
| 6. | $\frac{dN_{Clb2}}{dt}=k_{s,b2}\cdot\mu\cdot V\cdot N_{Mb2}-\Gamma_{b2}\cdot N_{Clb2}-\frac{k_{a,b2}}{V}\cdot N_{Clb2}\cdot\sum_{i=0}^{9} N_{{Sic1P}_{i}}$  $+k_{d,b2}\cdot\sum_{i=0}^{9} N_{{Sb2P}_{i}}+\gamma_{s1}\cdot\sum_{i=0}^{5} N_{{Sb2P}_{i}}+\gamma_{s1}^{'}\cdot\sum_{i=6}^{9} N_{{Sb2P}_{i}}$ |
| Equations for phosphatases | |
| 7. | $\frac{dN_{\mathrm{Hbf}}}{dt}=\left( \gamma_{\mathrm{hbf}}+\mu\right)\cdot V\cdot T_{\mathrm{Hbf}}\cdot N_{\mathrm{Mhbf}}-\gamma_{\mathrm{hbf}}\cdot N_{\mathrm{Hbf}}$ |
| 8. | $\frac{dN_{Ht1}}{dt}=k_{s,ht1}\cdot V\cdot N_{Mht1}-\Gamma_{ht1}\cdot N_{Ht1}$ |
| 9. | $\frac{dN_{Cdc14}}{dt}=\left( \gamma_{c14}+\mu\right)\cdot V\cdot T_{Cdc14}\cdot N_{Mc14}-\gamma_{c14}\cdot N_{Cdc14}-\frac{k_{a,c14}}{V}\cdot N_{Cdc14}\cdot\sum_{i=0}^{3} N_{{Net1P}_{i}}+\left( k_{d,c14}+\gamma_{t1} \right)\cdot\sum_{i=0}^{3} N_{\mathrm{RENTP}_{i}}+R_{p,t1}\cdot N_{\mathrm{RENTP}_{3}}$ |
| Equations for Whi5 | |
| 10. | $\frac{dN_{Whi5}}{dt}=\left( \gamma_{i5}+\mu\right)\cdot V\cdot T_{Whi5}\cdot N_{Mi5}+R_{dp,i5}\cdot N_{{Whi5P}_{1}}+\left( k_{d,\mathrm{bf}}+\gamma_{\mathrm{bf}} \right)\cdot N_{\mathrm{Cmp}}-\left( \frac{k_{a,\mathrm{bf}}}{V}\cdot N_{\mathrm{SBF}}+R_{p,i5}+\gamma_{i5} \right)\cdot N_{Whi5}$ |
| 11. | $\frac{dN_{{Whi5P}_{i}}}{dt}=R_{p,i5}\cdot N_{{Whi5P}_{i-1}}+R_{dp,i5}\cdot N_{{Whi5P}_{i+1}}+\left( k_{d,\mathrm{bf}}+\gamma_{\mathrm{bf}} \right)\cdot N_{\mathrm{CmpP}_{i}}$  $-\left( \frac{k_{a,\mathrm{bf}}}{V}\cdot N_{\mathrm{SBF}}+R_{p,i5}+R_{dp,i5}+\gamma_{i5} \right)\cdot N_{{Whi5P}_{i}} \mathrm{for} 1\leq i\leq2$ |
| 12. | $\frac{dN_{{Whi5P}_{3}}}{dt}=R_{p,i5}\cdot N_{{Whi5P}_{2}}+R_{dp,i5}\cdot N_{{Whi5P}_{4}}+k_{p,i5}\cdot N_{\mathrm{CmpP}_{2}}-\left( R_{p,i5}+R_{dp,i5}+\gamma_{i5} \right)\cdot N_{{Whi5P}_{3}}$ |
| 13. | $\frac{dN_{{Whi5P}_{i}}}{dt}=R_{p,i5}\cdot N_{{Whi5P}_{i-1}}+R_{dp,i5}\cdot N_{{Whi5P}_{i+1}}$  $-\left( R_{p,i5}+R_{dp,i5}+\gamma_{i5} \right)\cdot N_{{Whi5P}_{i}} \mathrm{for} 4\leq i\leq9$ |
| 14. | $\frac{dN_{{Whi5P}_{10}}}{dt}=R_{p,i5}\cdot N_{{Whi5P}_{9}}-\left( R_{dp,i5}+\gamma_{i5} \right)\cdot N_{{Whi5P}_{10}}$ |
| Equations for Whi5-SBF complexes | |
| 15. | $\frac{dN_{\mathrm{Cmp}}}{dt}=\frac{k_{a,\mathrm{bf}}}{V}\cdot N_{\mathrm{SBF}}\cdot N_{Whi5}+R_{dp,i5}\cdot N_{\mathrm{CmpP}_{1}}-\left( {R_{p,i5}+k}_{d,\mathrm{bf}}+\gamma_{\mathrm{bf}}+\gamma_{i5} \right)\cdot N_{\mathrm{Cmp}}$ |
| 16. | $\frac{dN_{\mathrm{CmpP}_{1}}}{dt}=\frac{k_{a,\mathrm{bf}}}{V}\cdot N_{\mathrm{SBF}}\cdot N_{{Whi5P}_{1}}+R_{p,i5}\cdot N_{\mathrm{Cmp}}+R_{dp,i5}\cdot N_{\mathrm{CmpP}_{2}}$  $-\left( {R_{p,i5}+R_{dp,i5}+k}_{d,\mathrm{bf}}+\gamma_{\mathrm{bf}}+\gamma_{i5} \right)\cdot N_{\mathrm{CmpP}_{1}}$ |
| 17. | $\frac{dN_{\mathrm{CmpP}_{2}}}{dt}=\frac{k_{a,\mathrm{bf}}}{V}\cdot N_{\mathrm{SBF}}\cdot N_{{Whi5P}_{2}}+R_{p,i5}\cdot N_{\mathrm{CmpP}_{1}}-\left( {R_{p,i5}+R_{dp,i5}+k}_{d,\mathrm{bf}}+\gamma_{\mathrm{bf}}+\gamma_{i5} \right)\cdot N_{\mathrm{CmpP}_{2}}$ |
| Equations for SBF, transcription factor for *CLN1*, *CLN2* and *CLB5* | |
| 18. | $\frac{dN_{\mathrm{SBF}}}{dt}=\left( \gamma_{\mathrm{bf}}+\mu\right)\cdot V\cdot T_{\mathrm{SBF}}\cdot N_{\mathrm{Msbf}}+R_{dp,\mathrm{bf}}\cdot N_{\mathrm{SBFP}_{1}}+\left( k_{d,\mathrm{bf}}+\gamma_{i5} \right)\cdot\sum_{i=0}^{2} N_{\mathrm{CmpP}_{i}}+R_{p,i5}\cdot N_{\mathrm{CmpP}_{2}}-\left( \frac{k_{a,\mathrm{bf}}}{V}\cdot\sum_{i=0}^{2} N_{{Whi5P}_{i}}+R_{p,\mathrm{bf}}+\gamma_{\mathrm{bf}} \right)\cdot N_{\mathrm{SBF}}$ |
| 19. | $\frac{dN_{\mathrm{SBFP}_{i}}}{dt}=R_{p,\mathrm{bf}}\cdot N_{\mathrm{SBFP}_{i-1}}+R_{dp,\mathrm{bf}}\cdot N_{\mathrm{SBFP}_{i+1}}$  $-\left( R_{p,\mathrm{bf}}+R_{dp,\mathrm{bf}}+\gamma_{\mathrm{bf}} \right)\cdot N_{\mathrm{SBFP}_{i}} \mathrm{for} 1\leq i\leq3$ |
| 20. | $\frac{dN_{\mathrm{SBFP}_{4}}}{dt}=R_{p,\mathrm{bf}}\cdot N_{\mathrm{SBFP}_{3}}-\left( R_{dp,\mathrm{bf}}+\gamma_{\mathrm{bf}} \right)\cdot N_{\mathrm{SBFP}_{4}}$ |
| Equations for Cdh1 | |
| 21. | $\frac{dN_{Cdh1}}{dt}={\left( \gamma_{h1}+\mu\right)\cdot V\cdot T_{Cdh1}\cdot N_{Mh1}+R}_{dp,h1}\cdot N_{{Cdh1P}_{1}}-\left( R_{p,h1}+\gamma_{h1} \right)\cdot N_{Cdh1}$ |
| 22. | $\frac{dN_{{Cdh1P}_{i}}}{dt}={R_{p,h1}\cdot N_{{Cdh1P}_{i-1}}+R}_{dp,h1}\cdot N_{{Cdh1P}_{i+1}}-\left( R_{p,h1}+R_{dp,h1}+\gamma_{h1} \right)\cdot N_{{Cdh1P}_{i}} \mathrm{for} 1\leq i\leq10$ |
| 23. | $\frac{dN_{{Cdh1P}_{11}}}{dt}=R_{p,h1}\cdot N_{{Cdh1P}_{10}}-\left( R_{dp,h1}+\gamma_{h1} \right)\cdot N_{{Cdh1P}_{11}}$ |
| Equations for Swi5, transcription factor for *SIC1* | |
| 24. | $\frac{dN_{Swi5}}{dt}={k_{s,swi5}\cdot V\cdot N_{Mswi5}+R}_{dp,swi5}\cdot N_{{Swi5P}_{1}}-\left( R_{p,swi5}+\gamma_{swi5} \right)\cdot N_{Swi5}$ |
| 25. | $\frac{dN_{{Swi5P}_{i}}}{dt}={R_{p,swi5}\cdot N_{{Swi5P}_{i-1}}+R}_{dp,swi5}\cdot N_{{Swi5P}_{i+1}}-\left( R_{p,swi5}+R_{dp,swi5}+\gamma_{swi5} \right)\cdot N_{{Swi5P}_{i}} \mathrm{for} 1\leq i\leq2$ |
| 26. | $\frac{dN_{{Swi5P}_{3}}}{dt}=R_{p,swi5}\cdot N_{{Swi5P}_{2}}-\left( R_{dp,swi5}+\gamma_{swi5} \right)\cdot N_{{Swi5P}_{3}}$ |
| Equations for Sic1 | |
| 27. | $\frac{dN_{Sic1}}{dt}={k_{s,s1}\cdot V\cdot N_{Ms1}+1\cdot R}_{dp,s1}\cdot N_{{Sic1P}_{1}}+\left( k_{d,b5}+\Gamma_{b5} \right)\cdot N_{Sb5}+\left( k_{d,b2}+\Gamma_{b2} \right)\cdot N_{Sb2}$  $-\left( {\frac{k_{a,b2}}{V}\cdot N_{Clb2}+\frac{k_{a,b5}}{V}\cdot N_{Clb5}+9\cdot R}_{p,s1}+\gamma_{s1} \right)\cdot N_{Sic1}$ |
| 28. | $\frac{dN_{{Sic1P}_{i}}}{dt}={{\left( 9-i-1 \right)\cdot R}_{p,s1}\cdot N_{{Sic1P}_{i-1}}+\left( i+1 \right)\cdot R}_{dp,s1}\cdot N_{{Sic1P}_{i+1}}$  $+\left( k_{d,b5}+\Gamma_{b5} \right)\cdot N_{{Sb5P}_{i}}+\left( k_{d,b2}+\Gamma_{b2} \right)\cdot N_{{Sb2P}_{i}}-\left( \frac{k_{a,b2}}{V}\cdot N_{Clb2}+\frac{k_{a,b5}}{V}\cdot N_{Clb5}+{\left( 9-i \right)\cdot R}_{p,s1}{+i\cdot R}_{dp,s1}+\gamma_{s1} \right)\cdot N_{{Sic1P}_{i}}$  $\mathrm{for} 1\leq i\leq5$ |
| 29. | $\frac{dN_{{Sic1P}_{i}}}{dt}={{\left( 9-i-1 \right)\cdot R}_{p,s1}\cdot N_{{Sic1P}_{i-1}}+\left( i+1 \right)\cdot R}_{dp,s1}\cdot N_{{Sic1P}_{i+1}}$  $+\left( k_{d,b5}+\Gamma_{b5} \right)\cdot N_{{Sb5P}_{i}}+\left( k_{d,b2}+\Gamma_{b2} \right)\cdot N_{{Sb2P}_{i}}-\left( \frac{k_{a,b2}}{V}\cdot N_{Clb2}+\frac{k_{a,b5}}{V}\cdot N_{Clb5}+{\left( 9-i \right)\cdot R}_{p,s1}{+i\cdot R}_{dp,s1}+\gamma_{s1}^{'} \right)\cdot N_{{Sic1P}_{i}}$  $\mathrm{for} 6\leq i\leq8$ |
| 30. | $\frac{dN_{{Sic1P}_{9}}}{dt}={1\cdot R}_{p,s1}\cdot N_{{Sic1P}_{8}}+\left( k_{d,b5}+\Gamma_{b5} \right)\cdot N_{{Sb5P}_{9}}+\left( k_{d,b2}+\Gamma_{b2} \right)\cdot N_{{Sb2P}_{9}}$  $-\left( \frac{k_{a,b2}}{V}\cdot N_{Clb2}+\frac{k_{a,b5}}{V}\cdot N_{Clb5}{+9\cdot R}_{dp,s1}+\gamma_{s1}^{'} \right)\cdot N_{{Sic1P}_{9}}$ |
| Equations for Sic1-Clb2 complexes | |
| 31. | $\frac{dN_{Sb2}}{dt}=\frac{k_{a,b2}}{V}\cdot N_{Clb2}\cdot N_{Sic1}+{1\cdot R}_{dp,s1}\cdot N_{{Sb2P}_{1}}-\left( {k_{d,b2}+\Gamma_{b2}+9\cdot R}_{p,s1}+\gamma_{s1} \right)\cdot N_{Sb2}$ |
| 32. | $\frac{dN_{{Sb2P}_{i}}}{dt}=\frac{k_{a,b2}}{V}\cdot N_{Clb2}\cdot N_{{Sic1P}_{i}}\cdot+{{\left( 9-i-1 \right)\cdot R}_{p,s1}\cdot N_{{Sb2P}_{i-1}}+\left( i+1 \right)\cdot R}_{dp,s1}\cdot N_{{Sb2P}_{i+1}}$  $-\left( k_{d,b2}+\Gamma_{b2}+{\left( 9-i \right)\cdot R}_{p,s1}{+i\cdot R}_{dp,s1}+\gamma_{s1} \right)\cdot N_{{Sb2P}_{i}} \mathrm{for} 1\leq i\leq5$ |
| 33. | $\frac{dN_{{Sb2P}_{i}}}{dt}=\frac{k_{a,b2}}{V}\cdot N_{Clb2}\cdot N_{{Sic1P}_{i}}+{{\left( 9-i-1 \right)\cdot R}_{p,s1}\cdot N_{{Sb2P}_{i-1}}+\left( i+1 \right)\cdot R}_{dp,s1}\cdot N_{{Sb2P}_{i+1}}$  $-\left( k_{d,b2}+\Gamma_{b2}+{\left( 9-i \right)\cdot R}_{p,s1}{+i\cdot R}_{dp,s1}+\gamma_{s1}^{'} \right)\cdot N_{{Sb2P}_{i}} \mathrm{for} 6\leq i\leq8$ |
| 34. | $\frac{dN_{{Sb2P}_{9}}}{dt}=\frac{k_{a,b2}}{V}\cdot N_{Clb2}\cdot N_{{Sic1P}_{9}}+{1\cdot R}_{p,s1}\cdot N_{{Sb2P}_{8}}-\left( k_{d,b2}+\Gamma_{b2}{+9\cdot R}_{dp,s1}+\gamma_{s1}^{'} \right)\cdot N_{{Sb2P}_{9}}$ |
| Equations for Sic1-Clb5 complexes | |
| 35. | $\frac{dN_{Sb5}}{dt}=\frac{k_{a,b5}}{V}\cdot N_{Clb5}\cdot N_{Sic1}+{1\cdot R}_{dp,s1}\cdot N_{{Sb5P}_{1}}-\left( {k_{d,b5}+\Gamma_{b5}+9\cdot R}_{p,s1}+\gamma_{s1} \right)\cdot N_{Sb5}$ |
| 36. | $\frac{dN_{{Sb5P}_{i}}}{dt}=\frac{k_{a,b5}}{V}\cdot N_{Clb5}\cdot N_{{Sic1P}_{i}}+{{\left( 9-i-1 \right)\cdot R}_{p,s1}\cdot N_{{Sb5P}_{i-1}}+\left( i+1 \right)\cdot R}_{dp,s1}\cdot N_{{Sb5P}_{i+1}}$  $-\left( k_{d,b5}+\Gamma_{b5}+{\left( 9-i \right)\cdot R}_{p,s1}{+i\cdot R}_{dp,s1}+\gamma_{s1} \right)\cdot N_{{Sb5P}_{i}} \mathrm{for} 1\leq i\leq5$ |
| 37 | $\frac{dN_{{Sb5P}_{i}}}{dt}=\frac{k_{a,b5}}{V}\cdot N_{Clb5}\cdot N_{{Sic1P}_{i}}+{{\left( 9-i-1 \right)\cdot R}_{p,s1}\cdot N_{{Sb5P}_{i-1}}+\left( i+1 \right)\cdot R}_{dp,s1}\cdot N_{{Sb5P}_{i+1}}$  $-\left( k_{d,b5}+\Gamma_{b5}+{\left( 9-i \right)\cdot R}_{p,s1}{+i\cdot R}_{dp,s1}+\gamma_{s1}^{'} \right)\cdot N_{{Sb5P}_{i}} \mathrm{for} 6\leq i\leq8$ |
| 38. | $\frac{dN_{{Sb5P}_{9}}}{dt}=\frac{k_{a,b5}}{V}\cdot N_{Clb5}\cdot N_{{Sic1P}_{9}}+{1\cdot R}_{p,s1}\cdot N_{{Sb5P}_{8}}-\left( k_{d,b5}+\Gamma_{b5}{+9\cdot R}_{dp,s1}+\gamma_{s1}^{'} \right)\cdot N_{{Sb5P}_{9}}$ |
| Equations for Fkh2, transcription factor for *CLB2*, *CDC20* and *SWI5* | |
| 40. | $\frac{dN_{Fkh2}}{dt}={\left( \gamma_{kh2}+\mu\right)\cdot V\cdot T_{Fkh2}\cdot N_{Mfkh2}+R}_{dp,kh2}\cdot N_{{Fkh2P}_{1}}-\left( R_{p,kh2}+\gamma_{kh2} \right)\cdot N_{Fkh2}$ |
| 41. | $\frac{dN_{{Fkh2P}_{1}}}{dt}={R_{p,kh2}\cdot N_{Fkh2}+R}_{dp,kh2}\cdot N_{{Fkh2P}_{2}}-\left( R_{p,kh2}+R_{dp,kh2}+\gamma_{kh2} \right)\cdot N_{{Fkh2P}_{1}}$ |
| 42. | $\frac{dN_{{Fkh2P}_{2}}}{dt}=R_{p,kh2}\cdot N_{{Fkh2P}_{1}}-\left( R_{dp,kh2}+\gamma_{kh2} \right)\cdot N_{{Fkh2P}_{2}}$ |
| Equation for Cdc20 | |
| 43. | $\frac{dN_{Cdc20}}{dt}=k_{s,c20}\cdot V\cdot N_{Mc20}+\left( k_{d,c20}+\gamma_{\mathrm{apc}} \right)\cdot\sum_{i=0}^{11} N_{{APC20P}_{i}}$  $-\left( \frac{k_{a,c20}}{V}\cdot\sum_{i=0}^{11} \beta^{11-i}\cdot N_{\mathrm{APCP}_{i}}+\Gamma_{c20} \right)\cdot N_{Cdc20}$ |
| Equations for APC and APC-Cdc20 complexes | |
| 44. | $\frac{dN_{\mathrm{APC}}}{dt}=\left( \gamma_{\mathrm{apc}}+\mu\right)\cdot V\cdot T_{\mathrm{APC}}\cdot N_{\mathrm{Mapc}}+R_{dp,\mathrm{apc}}\cdot N_{\mathrm{APCP}_{1}}+\left( k_{d,c20}+\Gamma_{c20} \right)\cdot N_{APC20}$  $-\left( \beta^{11}\cdot\frac{k_{a,c20}}{V}\cdot N_{Cdc20}+R_{p,\mathrm{apc}}+\gamma_{\mathrm{apc}} \right)\cdot N_{\mathrm{APC}}$ |
| 45. | $\frac{dN_{\mathrm{APCP}_{i}}}{dt}=R_{p,\mathrm{apc}}\cdot N_{\mathrm{APCP}_{i-1}}+R_{dp,\mathrm{apc}}\cdot N_{\mathrm{APCP}_{i+1}}+\left( k_{d,c20}+\Gamma_{c20} \right)\cdot N_{{APC20P}_{i}}$  $-\left( \beta^{11-i}\cdot\frac{k_{a,c20}}{V}\cdot N_{Cdc20}+R_{p,\mathrm{apc}}+R_{dp,\mathrm{apc}}+\gamma_{\mathrm{apc}} \right)\cdot N_{\mathrm{APCP}_{i}} \mathrm{for} 1\leq i\leq10$ |
| 46. | $\frac{dN_{\mathrm{APCP}_{11}}}{dt}=R_{p,\mathrm{apc}}\cdot N_{\mathrm{APCP}_{10}}+\left( k_{d,c20}+\Gamma_{c20} \right)\cdot N_{{APC20P}_{11}}$  $-\left( \beta^{0}\cdot\frac{k_{a,c20}}{V}\cdot N_{Cdc20}+R_{dp,\mathrm{apc}}+\gamma_{\mathrm{apc}} \right)\cdot N_{\mathrm{APCP}_{11}}$ |
| 47. | $\frac{dN_{APC20}}{dt}=\beta^{11}\cdot\frac{k_{a,c20}}{V}\cdot N_{Cdc20}\cdot N_{\mathrm{APC}}+R_{dp,\mathrm{apc}}\cdot N_{{APC20P}_{1}}-\left( k_{d,c20}+R_{p,\mathrm{apc}}+\Gamma_{c20}+\gamma_{\mathrm{apc}} \right)\cdot N_{APC20}$ |
| 48. | $\frac{dN_{{APC20P}_{i}}}{dt}=\beta^{11-i}\cdot\frac{k_{a,c20}}{V}\cdot N_{Cdc20}\cdot N_{\mathrm{APCP}_{i}}+R_{p,\mathrm{apc}}\cdot N_{{APC20P}_{i-1}}+R_{dp,\mathrm{apc}}\cdot N_{{APC20P}_{i+1}}$  $-\left( k_{d,c20}+R_{p,\mathrm{apc}}+R_{dp,\mathrm{apc}}+\Gamma_{c20}+\gamma_{\mathrm{apc}} \right)\cdot N_{{APC20P}_{i}} \mathrm{for} 1\leq i\leq10$ |
| 49. | $\frac{dN_{{APC20P}_{11}}}{dt}=\beta^{0}\cdot\frac{k_{a,c20}}{V}\cdot N_{Cdc20}\cdot N_{\mathrm{APCP}_{11}}+R_{p,\mathrm{apc}}\cdot N_{{APC20P}_{10}}-\left( k_{d,c20}+R_{dp,\mathrm{apc}}+\Gamma_{c20}+\gamma_{\mathrm{apc}} \right)\cdot N_{{APC20P}_{11}}$ |
| Equations for Net1 | |
| 50. | $\frac{dN_{Net1}}{dt}=\left( \gamma_{t1}+\mu\right)\cdot V\cdot T_{Net1}\cdot N_{Mt1}+R_{dp,t1}\cdot N_{{Net1P}_{1}}+\left( k_{d,c14}+\gamma_{c14} \right)\cdot N_{\mathrm{RENT}}$  $-\left( \frac{k_{a,c14}}{V}\cdot N_{Cdc14}+R_{p,t1}+\gamma_{t1} \right)\cdot N_{Net1}$ |
| 51. | $\frac{dN_{{Net1P}_{i}}}{dt}=R_{p,t1}\cdot N_{{Net1P}_{i-1}}+R_{dp,t1}\cdot N_{{Net1P}_{i+1}}+\left( k_{d,c14}+\gamma_{c14} \right)\cdot N_{\mathrm{RENTP}_{i}}$  $-\left( \frac{k_{a,c14}}{V}\cdot N_{Cdc14}+R_{p,t1}+R_{dp,t1}+\gamma_{t1} \right)\cdot N_{{Net1P}_{i}} \mathrm{for} 1\leq i\leq3$ |
| 52. | $\frac{dN_{{Net1P}_{4}}}{dt}=R_{p,t1}\cdot N_{{Net1P}_{3}}+R_{dp,t1}\cdot N_{{Net1P}_{5}}+k_{p,t1}\cdot N_{\mathrm{RENTP}_{3}}-\left( R_{p,t1}+R_{dp,t1}+\gamma_{t1} \right)\cdot N_{{Net1P}_{4}}$ |
| 53. | $\frac{dN_{{Net1P}_{i}}}{dt}=R_{p,t1}\cdot N_{{Net1P}_{i-1}}+R_{dp,t1}\cdot N_{{Net1P}_{i+1}}-\left( R_{p,t1}+R_{dp,t1}+\gamma_{t1} \right)\cdot N_{{Net1P}_{i}} \mathrm{for} 5\leq i\leq7$ |
| 54. | $\frac{dN_{{Net1P}_{8}}}{dt}=R_{p,t1}\cdot N_{{Net1P}_{7}}-\left( R_{dp,t1}+\gamma_{t1} \right)\cdot N_{{Net1P}_{8}}$ |
| Equations for RENT complexes | |
| 55. | $\frac{dN_{\mathrm{RENT}}}{dt}=\frac{k_{a,c14}}{V}\cdot N_{Cdc14}\cdot N_{Net1}+R_{dp,t1}\cdot N_{\mathrm{RENTP}_{1}}$  $-\left( k_{d,c14}+R_{p,t1}+\gamma_{t1}+\gamma_{c14} \right)\cdot N_{\mathrm{RENT}}$ |
| 56. | $\frac{dN_{\mathrm{RENTP}_{i}}}{dt}={\frac{k_{a,c14}}{V}\cdot N_{Cdc14}\cdot N_{{Net1P}_{i}}+R}_{p,t1}\cdot N_{\mathrm{RENTP}_{i-1}}+R_{dp,t1}\cdot N_{\mathrm{RENTP}_{i+1}}$  $-\left( {k_{d,c14}+R}_{p,t1}+R_{dp,t1}+\gamma_{t1}+\gamma_{c14} \right)\cdot N_{\mathrm{RENTP}_{i}} \mathrm{for} 1\leq i\leq2$ |
| 57. | $\frac{dN_{\mathrm{RENTP}_{3}}}{dt}={\frac{k_{a,c14}}{V}\cdot N_{Cdc14}\cdot N_{{Net1P}_{3}}+R}_{p,t1}\cdot N_{\mathrm{RENTP}_{2}}-\left( {k_{d,c14}+R}_{p,t1}+R_{dp,t1}+\gamma_{t1}+\gamma_{c14} \right)\cdot N_{\mathrm{RENTP}_{3}}$ |
| Equations for regulated mRNAs | |
| 58. | $\frac{dN_{Mn1}}{dt}=k_{s,mn1}+k_{s,mn1}^{'}\cdot N_{G_{a,n1}}-\gamma_{mn1}\cdot N_{Mn1}$ |
| 59. | $\frac{dN_{Mn2}}{dt}=k_{s,mn2}+k_{s,mn2}^{'}\cdot N_{G_{a,n2}}-\gamma_{mn2}\cdot N_{Mn2}$ |
| 60. | $\frac{dN_{Mb5}}{dt}=k_{s,mb5}+k_{s,mb5}^{'}\cdot N_{G_{a,b5}}-\gamma_{mb5}\cdot N_{Mb5}$ |
| 61. | $\frac{dN_{Mb2}}{dt}=k_{s,mb2}+k_{s,mb2}^{'}\cdot N_{G_{a,b2}}-\gamma_{mb2}\cdot N_{Mb2}$ |
| 62. | $\frac{dN_{Ms1}}{dt}=k_{s,ms1}+k_{s,ms1}^{'}\cdot N_{G_{a,s1}}-\gamma_{ms1}\cdot N_{Ms1}$ |
| 63. | $\frac{dN_{Mc20}}{dt}=k_{s,mc20}+k_{s,mc20}^{'}\cdot N_{G_{a,c20}}-\gamma_{mc20}\cdot N_{Mc20}$ |
| 64. | $\frac{dN_{swi5}}{dt}=k_{s,mswi5}\cdot N_{G_{a,swi5}}-\gamma_{mswi5}\cdot N_{swi5}$ |
| Equations for unregulated mRNAs | |
| 65. | $\frac{dN_{Mn3}}{dt}=k_{s,mn3}-\gamma_{mn3}\cdot N_{Mn3}$ |
| 66. | $\frac{dN_{Mi5}}{dt}=k_{s,mi5}-\gamma_{mi5}\cdot N_{Mi5}$ |
| 67. | $\frac{dN_{\mathrm{Msbf}}}{dt}=k_{s,\mathrm{msbf}}-\gamma_{\mathrm{msbf}}\cdot N_{\mathrm{Msbf}}$ |
| 68. | $\frac{dN_{Mh1}}{dt}=k_{s,mh1}-\gamma_{mh1}\cdot N_{Mh1}$ |
| 69. | $\frac{dN_{Mfkh2}}{dt}=k_{s,mfkh2}-\gamma_{mfkh2}\cdot N_{Mfkh2}$ |
| 70. | $\frac{dN_{\mathrm{Mapc}}}{dt}=k_{s,\mathrm{mapc}}-\gamma_{\mathrm{mapc}}\cdot N_{\mathrm{Mapc}}$ |
| 71. | $\frac{dN_{Mt1}}{dt}=k_{s,mt1}-\gamma_{mt1}\cdot N_{Mt1}$ |
| 72. | $\frac{dN_{Mc14}}{dt}=k_{s,mc14}-\gamma_{mc14}\cdot N_{Mc14}$ |
| 73. | $\frac{dN_{\mathrm{Mhbf}}}{dt}=k_{s,\mathrm{mhbf}}-\gamma_{\mathrm{mhbf}}\cdot N_{\mathrm{Mhbf}}$ |
| 74. | $\frac{dN_{Mht1}}{dt}=k_{s,mht1}-\gamma_{mht1}\cdot N_{Mht1}$ |
| Equations for regulated genes | |
| 75. | $\frac{dN_{G_{a,n1}}}{dt}=\left( \frac{k_{a,gn1}}{V} \right)\cdot N_{\mathrm{SBF}}\cdot\left( 1-N_{G_{a,n1}} \right)-k_{d,gn1}\cdot N_{G_{a,n1}}$ |
| 76. | $\frac{dN_{G_{a,n2}}}{dt}=\left( \frac{k_{a,gn2}}{V} \right)\cdot N_{\mathrm{SBF}}\cdot\left( 1-N_{G_{a,n2}} \right)-k_{d,gn2}\cdot N_{G_{a,n2}}$ |
| 77. | $\frac{dN_{G_{a,b5}}}{dt}=\left( \frac{k_{a,gb5}}{V} \right)\cdot N_{\mathrm{SBF}}\cdot\left( 1-N_{G_{a,b5}} \right)-k_{d,gb5}\cdot N_{G_{a,b5}}$ |
| 78. | $\frac{dN_{G_{a,b2}}}{dt}=\left( \frac{k_{a,gb2}}{V} \right)\cdot N_{{Fkh2P}_{2}}\cdot\left( 1-N_{G_{a,b2}} \right)-k_{d,gb2}\cdot N_{G_{a,b2}}$ |
| 79. | $\frac{dN_{G_{a,s1}}}{dt}=\left( \frac{k_{a,gs1}}{V} \right)\cdot N_{Swi5}\cdot\left( 1-N_{G_{a,s1}} \right)-k_{d,gs1}\cdot N_{G_{a,s1}}$ |
| 80. | $\frac{dN_{G_{a,c20}}}{dt}=\left( \frac{k_{a,gc20}}{V} \right)\cdot N_{{Fkh2P}_{2}}\cdot\left( 1-N_{G_{a,c20}} \right)-k_{d,gc20}\cdot N_{G_{a,c20}}$ |
| 81. | $\frac{dN_{G_{a,swi5}}}{dt}=\left( \frac{k_{a,gswi5}}{V} \right)\cdot N_{{Fkh2P}_{2}}\cdot\left( 1-N_{G_{a,swi5}} \right)-k_{d,gswi5}\cdot N_{G_{a,swi5}}$ |
| 82. | $\frac{dN_{G_{a,rad27}}}{dt}=\left( \frac{k_{a,grd27}}{V} \right)\cdot\left( \epsilon_{rd,n3}\cdot N_{Cln3}+\epsilon_{rd,n1}\cdot N_{Cln1}{+\epsilon}_{rd,n2}\cdot N_{Cln2}+\epsilon_{rd,b5}\cdot N_{Clb5} \right)\cdot\left( 1-N_{G_{a,rad27}} \right)-k_{d,grd27}\cdot N_{G_{a,rad27}}$ |

| $\Gamma_{b5}=\gamma_{b5}+\left( \frac{\gamma_{b5}^{'}}{V} \right)\cdot N_{Cdc20A}$ |
| --- |
| $\Gamma_{b2}=\gamma_{b2}+\left( \frac{\gamma_{b2}^{'}}{V} \right)\cdot N_{Cdh1}+\left( \frac{\gamma_{b2}^{''}}{V} \right)\cdot N_{Cdc20A}$ |
| $\Gamma_{c20}=\gamma_{c20}+\left( \frac{\gamma_{c20}^{'}}{V} \right)\cdot N_{Cdh1}$ |
| ${\Gamma_{ht1}=\gamma}_{ht1}+\left( \frac{\gamma_{ht1}^{'}}{V} \right)\cdot N_{Cdc20A}$ |
| $R_{p,i5}=\left( k_{p,i5}\cdot N_{Cln3}+k_{p,i5}^{'}\cdot N_{Cln1}+k_{p,i5}^{''}\cdot N_{Cln2} \right)/V$ |
| $R_{dp,i5}=\left( k_{dp,i5}\cdot N_{\mathrm{Hbf}}+k_{dp,i5}^{'}\cdot N_{Cdc14} \right)/V$ |
| $R_{p,\mathrm{bf}}=\left( k_{p,\mathrm{bf}}\cdot N_{Clb2} \right)/V$ |
| $R_{dp,\mathrm{bf}}=\left( k_{dp,\mathrm{bf}}\cdot N_{\mathrm{Hbf}}+k_{dp,\mathrm{bf}}^{'}\cdot N_{Cdc14} \right)/V$ |
| $R_{p,h1}=\left( k_{p,h1}\cdot N_{Cln1}+k_{p,h1}^{'}\cdot N_{Cln2}+k_{p,h1}^{''}\cdot N_{Clb5}+k_{p,h1}^{'''}\cdot N_{Clb2} \right)/V$ |
| $R_{dp,h1}=\left( k_{dp,h1}\cdot N_{\mathrm{Hbf}}+k_{dp,h1}^{'}\cdot N_{Cdc14} \right)/V$ |
| $R_{p,swi5}=\left( k_{p,swi5}\cdot N_{Clb5}+k_{p,swi5}^{'}\cdot N_{Clb2} \right)/V$ |
| $R_{dp,swi5}=\left( k_{dp,swi5}\cdot N_{\mathrm{Hbf}}+k_{dp,swi5}^{'}\cdot N_{Cdc14} \right)/V$ |
| $R_{p,s1}=\left( k_{p,s1}\cdot N_{Cln3}+k_{p,s1}^{'}\cdot N_{Cln1}+k_{p,s1}^{''}\cdot N_{Cln2}+k_{p,s1}^{'''}\cdot N_{Clb5}+k_{p,s1}^{''''}\cdot N_{Clb2} \right)/V$ |
| $R_{dp,s1}=\left( k_{dp,s1}\cdot N_{\mathrm{Hbf}}+k_{dp,s1}^{'}\cdot N_{Cdc14} \right)/V$ |
| $R_{p,kh2}=\left( k_{p,kh2}\cdot N_{Cln1}+k_{p,kh2}^{'}\cdot N_{Cln2}+k_{p,kh2}^{''}\cdot N_{Clb5}+k_{p,kh2}^{'''}\cdot N_{Clb2}+k_{p,kh2}^{''''}\cdot N_{Cln3} \right)/V$ |
| $R_{dp,kh2}=\left( k_{dp,kh2}\cdot N_{\mathrm{Hbf}}+k_{dp,kh2}^{'}\cdot N_{Cdc14} \right)/V$ |
| $R_{p,\mathrm{apc}}=\left( k_{p,\mathrm{apc}}\cdot N_{Clb5}+k_{p,\mathrm{apc}}^{'}\cdot N_{Clb2} \right)/V$ |
| $R_{dp,\mathrm{apc}}=\left( k_{dp,\mathrm{apc}}\cdot N_{\mathrm{Hbf}}+k_{dp,\mathrm{apc}}^{'}\cdot N_{Cdc14} \right)/V$ |
| $R_{p,t1}=\left( k_{p,t1}\cdot N_{Clb2} \right)/V$ |
| $R_{dp,t1}=\left( k_{dp,t1}\cdot N_{Ht1} \right)/V$ |
| $N_{Cdc20A}=\sum_{i=0}^{11} N_{{APC20P}_{i}}$ |
| ${bud}_{ss}=\varepsilon_{bd,n3}\cdot N_{Cln3}+\varepsilon_{bd,n1}\cdot N_{Cln1}{+\varepsilon}_{bd,n2}\cdot N_{Cln2}+\varepsilon_{bd,b5}\cdot N_{Clb5}$ |
| ${dna}_{ss}=\varepsilon_{na,b5}\cdot N_{Clb5}+\varepsilon_{na,b2}\cdot N_{Clb2}$ |

**Table C:** Parameter values used for simulations of wild-type cells.

| Parameter | min^-1^ | Parameter | min^-1^ | Parameter | min^-1^ |
| --- | --- | --- | --- | --- | --- |
| $\mu$ | 0.007 | $\gamma_{mn3}$ | 1.0 | $k_{d,b5}$ | 0.05 |
| $\gamma_{n3}$ | 0.14 | $\gamma_{mn1}$ | 0.25 | $k_{d,b2}$ | 0.1 |
| $\gamma_{n1}$ | 0.14 | $\gamma_{mn2}$ | 0.25 | $k_{d,c20}$ | 0.01 |
| $\gamma_{n2}$ | 0.14 | $\gamma_{mb5}$ | 0.25 | $k_{d,gn1}$ | 15.0 |
| $\gamma_{b5}$ | 0.0125 | $\gamma_{mb2}$ | 0.1 | $k_{d,gn2}$ | 15.0 |
| $\gamma_{b2}$ | 0.003 | $\gamma_{ms1}$ | 0.14 | $k_{d,gb5}$ | 10.0 |
| $\gamma_{i5}$ | 0.01 | $\gamma_{mc20}$ | 0.07 | $k_{d,gb2}$ | 5.0 |
| $\gamma_{\mathrm{bf}}$ | 0.01 | $\gamma_{mswi5}$ | 0.07 | $k_{d,gs1}$ | 5.0 |
| $\gamma_{ht1}$ | 0.001 | $\gamma_{mi5}$ | 0.14 | $k_{d,gc20}$ | 5.0 |
| $\gamma_{c14}$ | 0.02 | $\gamma_{\mathrm{msbf}}$ | 0.14 | $k_{d,gswi5}$ | 5.0 |
| $\gamma_{t1}$ | 0.015 | $\gamma_{mh1}$ | 0.07 | $k_{d,grd27}$ | 15.0 |
| $\gamma_{s1}$ | 0.001 | $\gamma_{mfkh2}$ | 0.07 | $k_{s,mn1}^{'}$ | 6.85 |
| $\gamma_{s1}^{'}$ | 0.85 | $\gamma_{\mathrm{mapc}}$ | 0.07 | $k_{s,mn2}^{'}$ | 12.0 |
| $\gamma_{h1}$ | 0.01 | $\gamma_{mt1}$ | 0.14 | $k_{s,mb5}^{'}$ | 5.75 |
| $\gamma_{kh2}$ | 0.001 | $\gamma_{mc14}$ | 0.07 | $k_{s,mb2}^{'}$ | 1.5 |
| $\gamma_{\mathrm{apc}}$ | 0.001 | $\gamma_{\mathrm{mhbf}}$ | 0.14 | $k_{s,ms1}^{'}$ | 3.0 |
| $\gamma_{c20}$ | 0.25 | $\gamma_{mht1}$ | 0.07 | $k_{s,mc20}^{'}$ | 0.5 |
| $\gamma_{\mathrm{hbf}}$ | 0.01 | $k_{d,bf}$ | 0.1 | $k_{s,mswi5}$ | 2.0 |
| $\gamma_{swi5}$ | 0.075 | $k_{d,c14}$ | 0.01 |  |  |
| Parameter | fL^-1^ min^-1^ | Parameter | fL^-1^ min^-1^ | Parameter | fL^-1^ min^-1^ |
| $k_{s,n1}$ | 0.3 | $k_{s,ht1}$ | 0.08 | $k_{s,c20}$ | 0.75 |
| $k_{s,n2}$ | 0.4 | $k_{s,s1}$ | 0.5 | $k_{s,swi5}$ | 0.195 |
| $k_{s,b5}$ | 0.05 |  |  |  |  |
| Parameter | fL^-1^ molec^-1^ min^-1^ | Parameter | fL^-1^ molec^-1^ min^-1^ | Parameter | fL^-1^ molec^-1^ min^-1^ |
| $\gamma_{b5}^{'}$ | 0.045 | $k_{p,i5}^{'}$ | 0.065 | $k_{p,s1}^{''}$ | 0.003 |
| $\gamma_{b2}^{'}$ | 0.0165 | $k_{p,i5}^{''}$ | 0.03 | $k_{p,s1}^{'''}$ | 0.38 |
| $\gamma_{b2}^{''}$ | 0.011 | $k_{dp,i5}$ | 0.012 | $k_{p,s1}^{''''}$ | 017 |
| $\gamma_{ht1}^{'}$ | 0.06 | $k_{dp,i5}^{'}$ | 0.25 | $k_{dp,s1}$ | 0.0025 |
| $\gamma_{c20}^{'}$ | 0.1 | $k_{p,bf}$ | 0.55 | $k_{dp,s1}^{'}$ | 0.1 |
| $k_{a,b2}$ | 0.6 | $k_{dp,bf}$ | 0.005 | $k_{p,kh2}$ | 0.001 |
| $k_{a,b5}$ | 0.105 | $k_{dp,\mathrm{bf}}^{'}$ | 0.125 | $k_{p,kh2}^{'}$ | 2.5×10^-4^ |
| $k_{a,bf}$ | 0.4 | $k_{p,h1}$ | 0.09 | $k_{p,kh2}^{''}$ | 0.001 |
| $k_{a,c14}$ | 0.067 | $k_{p,h1}^{'}$ | 0.03 | $k_{p,kh2}^{'''}$ | 0.055 |
| $k_{a,c20}$ | 0.0167 | $k_{p,h1}^{''}$ | 0.09 | $k_{p,kh2}^{''''}$ | 0.006 |
| $k_{a,gn1}$ | 0.25 | $k_{p,h1}^{'''}$ | 0.2 | $k_{dp,kh2}$ | 5.0×10^-4^ |
| $k_{a,gn2}$ | 0.25 | $k_{dp,h1}$ | 0.025 | $k_{dp,kh2}^{'}$ | 0.3 |
| $k_{a,gb5}$ | 5.0 | $k_{dp,h1}^{'}$ | 0.14 | $k_{p,apc}$ | 0.0 |
| $k_{a,gb2}$ | 0.2 | $k_{p,swi5}$ | 0.01 | $k_{p,\mathrm{apc}}^{'}$ | 0.015 |
| $k_{a,gs1}$ | 0.167 | $k_{p,swi5}^{'}$ | 0.09 | $k_{dp,apc}$ | 0.0035 |
| $k_{a,gc20}$ | 0.167 | $k_{dp,swi5}$ | 0.0 | $k_{dp,\mathrm{apc}}^{'}$ | 0.0 |
| $k_{a,gswi5}$ | 0.167 | $k_{dp,swi5}^{'}$ | 1.25 | $k_{p,t1}$ | 0.0375 |
| $k_{a,grd27}$ | 0.25 | $k_{p,s1}$ | 0.007 | $k_{dp,t1}$ | 0.05 |
| $k_{p,i5}$ | 0.265 | $k_{p,s1}^{'}$ | 0.009 |  |  |
| Parameter | fL^-1^ | Parameter | fL^-1^ | Parameter | fL^-1^ |
| $k_{s,b2}$ | 24.0 | $T_{Whi5}$ | 60.0 | $T_{Fkh2}$ | 10.0 |
| $T_{\mathrm{Hbf}}$ | 12.0 | $T_{\mathrm{SBF}}$ | 12.0 | $T_{\mathrm{APC}}$ | 8.0 |
| $T_{Cdc14}$ | 6.0 | $T_{Cdh1}$ | 15.0 | $T_{Net1}$ | 24.0 |
| Parameter | molec min^-1^ | Parameter | molec min^-1^ | Parameter | molec min^-1^ |
| $k_{s,mn3}$ | 7.5 | $k_{s,mc20}$ | 0.35 | $k_{s,mt1}$ | 1.05 |
| $k_{s,mn1}$ | 0.35 | $k_{s,mi5}$ | 0.6 | $k_{s,mc14}$ | 0.525 |
| $k_{s,mn2}$ | 0.35 | $k_{s,msbf}$ | 1.05 | $k_{s,mhbf}$ | 1.4 |
| $k_{s,mb5}$ | 0.35 | $k_{s,mh1}$ | 0.525 | $k_{s,mht1}$ | 0.7 |
| $k_{s,mb2}$ | 0.025 | $k_{s,mfkh2}$ | 0.525 |  |  |
| $k_{s,ms1}$ | 0.35 | $k_{s,mapc}$ | 0.525 |  |  |
| Parameter | Dimensionless | Parameter | Dimensionless | Parameter | Dimensionless |
| $\epsilon_{rd,n3}$ | 0.0 | $\epsilon_{bd,n3}$ | 1.0×10^-5^ | $\epsilon_{na,b5}$ | 0.05 |
| $\epsilon_{rd,n1}$ | 0.75 | $\epsilon_{bd,n1}$ | 0.045 | $\epsilon_{na,b2}$ | 0.025 |
| $\epsilon_{rd,n2}$ | 0.275 | $\epsilon_{bd,n2}$ | 0.0175 | $\beta$ | 0.525 |
| $\epsilon_{rd,b5}$ | 0.5 | $\epsilon_{bd,b5}$ | 0.015 | $f$ | 0.4 |
| Parameter | fL^-2^ min^-1^ |  |  |  |  |
| $k_{s,n3}$ | 0.0024 |  |  |  |  |

**Table D:** List of all the mutants simulated with the relevant modification of parameters.

| Serial  No. | Genotype | Parameter change | Comments |
| --- | --- | --- | --- |
| 1. | WT in Glucose | $\mu=0.007$ | Viable |
| 2. | WT in Galactose | $\mu=0.00467$ | Viable |
| 3. | WT in Gly-Eth | $\mu=0.00398$ | Viable |
| 4. | *cln1Δ* | $k_{s,mn1}=0.0$; $k_{s,mn1}^{'}=0.0$ | Viable |
| 5. | *cln2Δ* | $k_{s,mn2}=0.0$; $k_{s,mn2}^{'}=0.0$ | Viable |
| 6. | *clb5Δ* | $k_{s,mb5}=0.0875$; $k_{s,mb5}^{'}=1.4375$  (25% of WT values) | Viable |
| 7. | *cdh1Δ* | $k_{s,mh1}=0.0$ | Viable |
| 8. | *sic1Δ* | $k_{s,ms1}=0.0$; $k_{s,ms1}^{'}=0.0$ | Viable |
| 9. | *swi5Δ* | $k_{s,mswi5}=0.0$ | Viable |
| 10. | *cdc20Δ* | $k_{s,mc20}=0.0$; $k_{s,mc20}^{'}=0.0$ | Inviable, metaphase arrest |
| 11. | *cln1Δ cln2Δ* | $k_{s,mn1}=0.0$; $k_{s,mn1}^{'}=0.0$  $k_{s,mn2}=0.0$; $k_{s,mn2}^{'}=0.0$ | Viable |
| 12. | *clb5Δ clb6Δ* | $k_{s,mb5}$=0.0; $k_{s,mb5}^{'}$=0.0 | Viable |
| 13. | *clb1Δ clb2Δ* | $k_{s,mb2}$=0.0; $k_{s,mb2}^{'}$=0.0 | Inviable, G2 arrest |
| 14. | *cln1Δ cln2Δ cdh1Δ* | $k_{s,mn1}=0.0$; $k_{s,mn1}^{'}=0.0$  $k_{s,mn2}=0.0$; $k_{s,mn2}^{'}=0.0$  $k_{s,mh1}=0.0$ | Viable |
| 15. | *cln1Δ cln2Δ sic1Δ* | $k_{s,mn1}=0.0$; $k_{s,mn1}^{'}=0.0$  $k_{s,mn2}=0.0$; $k_{s,mn2}^{'}=0.0$  $k_{s,ms1}=0.0$; $k_{s,ms1}^{'}=0.0$ | Viable |
| 16. | *cln1Δ cln2Δ cln3Δ* | $k_{s,mn3}=0.0$ | Inviable, G1 arrest |
| 17. | *cdc20Δ clb5Δ* | $k_{s,mc20}=0.0$; $k_{s,mc20}^{'}=0.0$  $k_{s,mb5}=0.0875$; $k_{s,mb5}^{'}=1.4375$ | Inviable, metaphase arrest |
| 18. | *clb2-dbΔ* | $\gamma_{b2}^{'}=0.0016$(10% of WT)  $\gamma_{b2}^{''}=0.0$ | Inviable, telophase arrest |
| 19. | *clb2-dbΔ* in galactose | $\gamma_{b2}^{'}=0.0016$;  $\gamma_{b2}^{''}=0.0$; $\mu=0.00467$ | Inviable, telophase arrest |
| 20. | *clb2-dbΔ clb5Δ* | $\gamma_{b2}^{'}=0.0016$; $\gamma_{b2}^{''}=0.0$  $k_{s,mb5}=0.0875$; $k_{s,mb5}^{'}=1.4375$ | Inviable, telophase arrest |
| 21. | *clb2-dbΔ clb5Δ* in galactose | $\gamma_{b2}^{'}=0.0016$; $\gamma_{b2}^{''}=0.0$  $k_{s,mb5}=0.0875$; $k_{s,mb5}^{'}=1.4375$; $\mu=0.00467$ | Viable |
| 22. | *cln1Δ cln2Δ GAL-CLN2* | $k_{s,mn1}=1.5$; $k_{s,mn1}^{'}=0.0$; $\mu=0.00467$ | Viable |
| 23. | *GAL-CLB5* | $k_{s,mb5}=1.5$; $\mu=0.00467$ | Viable |
| 24. | *GAL-CLB5 sic1Δ* | $k_{s,mb5}=1.5$; $\mu=0.00467$  $k_{s,ms1}=0.0$; $k_{s,ms1}^{'}=0.0$ | Inviable |
| 25. | *GAL-CLB5 cdh1Δ* | $k_{s,mb5}=1.5$; $\mu=0.00467$  $k_{s,mh1}=0.0$ | Inviable |
| 26. | *clb5-dbΔ* | $\gamma_{b5}^{'}=0.0$ | Viable |
| 27. | *clb5-dbΔ sic1Δ* | $\gamma_{b5}^{'}=0.0$; $k_{s,ms1}=0.0$; $k_{s,ms1}^{'}=0.0$ | Inviable |
| 28. | *clb5-dbΔ cdh1Δ* | $\gamma_{b5}^{'}=0.0$; $k_{s,mh1}=0.0$ | Inviable |
| 29. | *GAL-CLB5-dbΔ* | $\gamma_{b5}^{'}=0.0$; $k_{s,mb5}=1.5$; $\mu=0.00467$ | Inviable |
| 30. | *GAL-CLB2* | $k_{s,mb2}=1.5$; $\mu=0.00467$ | Viable |
| 31. | *GAL-SIC1* | $k_{s,ms1}=1.5$; $\mu=0.00467$ | Viable |
| 32. | *clb2-dbΔ GAL-SIC1* | $\gamma_{b2}^{'}=0.0016$; $\gamma_{b2}^{''}=0.0$  $k_{s,ms1}=1.5$; $\mu=0.00467$ | Viable |
| 33. | *GAL-CLB2-dbΔ* | $\gamma_{b2}^{'}=0.0016$; $\gamma_{b2}^{''}=0.0$  $k_{s,mb2}=1.5$; $\mu=0.00467$ | Inviable, Telophase arrest |
| 34. | *clb2-dbΔ*  *Multicopy-SIC1* | $\gamma_{b2}^{'}=0.0016$; $\gamma_{b2}^{''}=0.0$  $k_{s,ms1}=1.50$; $k_{s,ms1}^{'}=9.0$  ($\equiv$ 3 copies) | Viable |
| 35. | *GAL-SIC1-dbΔ* | $k_{s,ms1}=1.5$; $\mu=0.00467$  $\gamma_{s1}^{'}=0.0$ | Inviable, G1 arrest |
| 36. | *sic1Δ cdh1Δ* | $k_{s,ms1}=0.0$; $k_{s,ms1}^{'}=0.0$  $k_{s,mh1}=0.0$ | Inviable |
| 37. | *swi5Δ cdh1Δ* | $k_{s,mswi5}=0.0$; $k_{s,mh1}=0.0$ | Inviable |
| 38. | *swi5Δ cdh1Δ GAL-SIC1* | $k_{s,mswi5}=0.0$; $k_{s,mh1}=0.0$; $k_{s,ms1}=1.5$; $\mu=0.00467$ | Viable |
| 39. | *Multicopy GAL-CLB2* | $k_{s,mb2}=12.0$; $\mu=0.00467$ | Inviable, telophase arrest |
| 40. | *cln1Δ cln2Δ clb5Δ clb6Δ* | $k_{s,mn1}=0.0$; $k_{s,mn1}^{'}=0.0$  $k_{s,mn2}=0.0$; $k_{s,mn2}^{'}=0.0$  $k_{s,mb5}$=0.0; $k_{s,mb5}^{'}$=0.0 | Inviable, G1 arrest |
| 41. | *cdc20-ts* | $k_{s,mc20}$=0.0; $k_{s,mc20}^{'}$=0.0 | Inviable, metaphase arrest |
| 42. | *net1-ts* | $k_{a,c14}=0.0134$ (20% of WT) | Viable |
| 43. | *APC-A* | $k_{p,\mathrm{apc}}^{'}=0.009$ | Viable |
| 44. | *cdc14-ts* | $k_{dp,i5}^{'}=0.0125$; $k_{dp,\mathrm{bf}}^{'}$=0.00625;  $k_{dp,h1}^{'}=0.007$; $k_{dp,swi5}^{'}$=0.0625;  $k_{dp,s1}^{'}=0.005$; $k_{dp,kh2}^{'}=0.05$  (5% of WT for all) | Inviable, telophase arrest |
| 45. | *GAL-CDC14* | $k_{s,mc14}=5.25$ | Inviable, G1 arrest |
| 46. | *GAL-NET1* | $k_{s,mt1}=5.25$ | Inviable |
| 47. | *GAL-CDC14 GAL-NET1* | $k_{s,mc14}=5.25$; $k_{s,mt1}=5.25$ | Viable |
| 48. | *cdc14-ts permissive temperature* | $k_{dp,i5}^{'}=0.025$; $k_{dp,\mathrm{bf}}^{'}$=0.0125;  $k_{dp,h1}^{'}=0.014$; $k_{dp,swi5}^{'}$=0.125;  $k_{dp,s1}^{'}=0.01$; $k_{dp,kh2}^{'}=0.1$  (10% of WT for all) | Viable |
| 49. | *cdc14-ts permissive temperature cdh1Δ* | $k_{dp,i5}^{'}=0.025$; $k_{dp,\mathrm{bf}}^{'}$=0.0125;  $k_{dp,h1}^{'}=0.014$; $k_{dp,swi5}^{'}$=0.125;  $k_{dp,s1}^{'}=0.01$; $k_{dp,kh2}^{'}=0.1$  $k_{s,mh1}=0.0$ | Inviable, telophase arrest |
| 50. | *cdc14-ts permissive temperature sic1Δ* | $k_{dp,i5}^{'}=0.025$; $k_{dp,\mathrm{bf}}^{'}$=0.0125;  $k_{dp,h1}^{'}=0.014$; $k_{dp,swi5}^{'}$=0.125;  $k_{dp,s1}^{'}=0.01$; $k_{dp,kh2}^{'}=0.1$  $k_{s,ms1}=0.0$; $k_{s,ms1}^{'}=0.0$ | Inviable, telophase arrest |
| 51. | *cdc14-ts GAL-SIC1* | $k_{dp,i5}^{'}=0.0125$; $k_{dp,\mathrm{bf}}^{'}$=0.00625;  $k_{dp,h1}^{'}=0.007$; $k_{dp,swi5}^{'}$=0.0625;  $k_{dp,s1}^{'}=0.005$; $k_{dp,kh2}^{'}=0.05$; $k_{s,ms1}=1.5$; $\mu=0.00467$ | Viable |
| 52. | *TAB6-1* | $k_{a,c14}=0.01$ (15% of WT) | Viable |

**Table E:** Average and coefficient of variation for cell cycle properties calculated under different growth conditions. Volumes (*V*) in fL; times (*T*) in min. The experimental values (in parentheses) are from Di Talia *et al* (Di Talia et al, 2007).

| Average | | | | | | |
| --- | --- | --- | --- | --- | --- | --- |
| Quantity | Glucose  (μ = 0.007 min^−1^) | | Galactose  (μ = 0.00467 min^−1^) | | Glycerol-Ethanol  (μ = 0.00398 min^−1^) | |
|  | Mother | Daughter | Mother | Daughter | Mother | Daughter |
| $V_{\mathrm{bir}}$ | 40.0 (40) | 27.1 (28) | 29.6 | 17.1 | 27.4 | 15.2 |
| $V_{\mathrm{div}}$ | 73.9 | 57.2 | 50.8 | 38.6 | 45.7 | 35.0 |
| $T_{\mathrm{div}}$ | 88.5 (87) | 108 (112) | 117 | 175.5 | 131 (133) | 211 (219) |
| $T_{Whi5}$ | 7.8 (~2) | 22.7 (20) | 17.0 | 69.0 | 21.9 (8) | 96.2 (94) |
| $T_{\mathrm{unbud}}$ | 16.5 (16) | 37.0 (37) | 31.2 | 91.0 | 37.2 (33) | 118 (126) |
| $T_{g1}$ | 25.3 | 43.8 | 39.6 | 96.0 | 45.3 | 123 |
| $T_{\mathrm{bud}}$ | 71.9 (72) | 71.0 (76) | 85.9 | 84.5 | 93.5 (101) | 92.8 (106) |
| Coefficient of Variation | | | | | | |
| $V_{\mathrm{bir}}$ | 0.25 (0.18) | 0.25 (0.20) | 0.24 | 0.24 | 0.23 | 0.23 |
| $V_{\mathrm{div}}$ | 0.22 | 0.20 | 0.20 | 0.21 | 0.19 | 0.22 |
| $T_{\mathrm{div}}$ | 0.16 (0.14) | 0.21 (0.22) | 0.24 | 0.32 | 0.27 | 0.34 |
| $T_{Whi5}$ | 0.82 | 0.79 | 1.22 | 0.76 | 1.25 | 0.70 |
| $T_{\mathrm{unbud}}$ | 0.54 (0.50) | 0.56 (0.50) | 0.77 | 0.59 | 0.81 | 0.58 |
| $T_{g1}$ | 0.35 | 0.45 | 0.58 | 0.54 | 0.63 | 0.55 |
| $T_{\mathrm{bud}}$ | 0.15 (0.17) | 0.15 (0.20) | 0.17 | 0.16 | 0.18 | 0.18 |

**Table F:** The average durations of cell cycle phases calculated from stochastic simulations for two different growth media: glucose (μ = 0.007 min^−1^) and glycerol-ethanol (μ = 0.00398 min^−1^). *T*_X,Y_ is the average time (min) between events X and Y. The experimental values are taken from Skotheim et al (Skotheim et al, 2008).

| Property | Genotype | Medium | | | | | | | |
| --- | --- | --- | --- | --- | --- | --- | --- | --- | --- |
|  |  | Glucose | | | | Gycerol-Ethanol | | | |
|  |  | Daughter | | Mother | | Daughter | | Mother | |
|  |  | Model | Expt. | Model | Expt. | Model | Expt. | Model | Expt. |
| *T*_bir,_*_CLN2_* | WT | 34.6 | 41.0 | 11.8 | 5.0 | 136.0 | 117.0 | 38.2 | 39.0 |
|  | *cln1Δ* | 60.0 | 39.0 | 18.2 |  | 232.3 | 123.0 | 78.8 |  |
|  | *cln2Δ* | 53.0 | 41.0 | 16.2 |  | 210.8 | 114.0 | 67.1 |  |
|  | *cln1Δ cln2Δ* | 87.1 | 83.0 | 29.3 | 43.0 | 290.6 | 239.0 | 130.4 | 245.0 |
| *T*_bir,bud_ | WT | 42.1 | 68.0 | 17.9 |  | 145.7 |  | 46.6 |  |
|  | *cln1Δ* | 73.8 | 81.0 | 30.3 |  | 249.3 |  | 96.3 |  |
|  | *cln2Δ* | 65.1 | 83.0 | 26.9 |  | 225.7 |  | 82.1 |  |
|  | *cln1Δ cln2Δ* | 130.3 | 170.0 | 81.4 |  | 356.8 |  | 205.2 |  |
| *T*_bir,_*_RAD27_* | WT | 41.9 |  | 19.4 |  | 140.2 |  | 45.4 |  |
|  | *cln1Δ* | 75.6 |  | 33.9 |  | 249.7 |  | 97.2 |  |
|  | *cln2Δ* | 65.4 |  | 29.3 |  | 222.1 |  | 81.2 |  |
|  | *cln1Δ cln2Δ* | 127.2 |  | 80.0 |  | 355.1 |  | 202.5 |  |
| *T*_bir,_*_CLB2_* | WT | 56.3 | 59.0 | 33.7 | 27.0 | 155.0 |  | 59.0 |  |
|  | *cln1Δ* | 92.4 |  | 52.4 |  | 264.3 |  | 112.6 |  |
|  | *cln2Δ* | 79.7 |  | 44.6 |  | 234.9 |  | 94.7 |  |
|  | *cln1Δ cln2Δ* | 143.5 | 120.0 | 95.1 | 95.0 | 368.0 |  | 212.2 |  |
| *T*_C_*_LN2,RAD27_* | WT | 7.3 | 4.0 | 7.5 | 0 | 4.2 |  | 7.2 |  |
|  | *cln1Δ* | 15.6 |  | 15.7 |  | 17.3 |  | 18.4 |  |
|  | *cln2Δ* | 12.5 |  | 13.1 |  | 11.2 |  | 14.2 |  |
|  | *cln1Δ cln2Δ* | 40.1 | 25.0 | 50.8 | 21.0 | 64.4 |  | 72.1 |  |
| *T_CLN2,_*_Whi5_ | WT | -9.2 | -3.0 | -3.7 | 0 | -28.5 |  | -12.0 |  |
|  | *cln1Δ* | -16.1 |  | -6.8 |  | -42.1 |  | -25.2 |  |
|  | *cln2Δ* | -15.8 |  | 6.0 |  | -43.3 |  | -24.2 |  |
|  | *cln1Δ cln2Δ* | -24 | 7.0 | -13.3 | 8.0 | -55.1 |  | -46.1 |  |

**Table G:** The standard deviations of the durations of cell cycle phases calculated from stochastic simulations for growth on glucose medium (μ = 0.007 min^−1^) and glycerol-ethanol medium (μ = 0.00398 min^−1^). The experimental values are taken from Skotheim et al (Skotheim et al, 2008).

| Property | Genotype | Growth Medium | | | | | | | |
| --- | --- | --- | --- | --- | --- | --- | --- | --- | --- |
|  |  | Glucose | | | | Gycerol-Ethanol | | | |
|  |  | Daughter | | Mother | | Daughter | | Mother | |
|  |  | Model | Expt. | Model | Expt. | Model | Expt. | Model | Expt. |
| *T*_bir,_*_CLN2_* | WT | 24.2 | 21.0 | 10.2 | 10.0 | 78.8 | 39.0 | 36.7 | 36.0 |
|  | *cln1Δ* | 37.1 | 18.0 | 20.3 |  | 76.8 | 49.0 | 65.6 |  |
|  | *cln2Δ* | 34.6 | 19.0 | 17.6 |  | 80.0 | 37.0 | 59.5 |  |
|  | *cln1Δ cln2Δ* | 41.4 | 47.0 | 31.7 | 28.0 | 71.4 | 71.0 | 76.2 | 76.0 |
| *T*_bir,bud_ | WT | 24.0 | 24.0 | 10.7 |  | 77.1 |  | 36.8 |  |
|  | *cln1Δ* | 36.3 | 24.0 | 21.4 |  | 75.2 |  | 65.5 |  |
|  | *cln2Δ* | 33.8 | 25.0 | 18.8 |  | 78.1 |  | 59.5 |  |
|  | *cln1Δ cln2Δ* | 37.0 | 79.0 | 31.6 |  | 70.8 |  | 70.7 |  |
| *T*_bir,_*_RAD27_* | WT | 23.9 |  | 10.4 |  | 77.2 |  | 36.2 |  |
|  | *cln1Δ* | 36.6 |  | 21.2 |  | 76.6 |  | 66.0 |  |
|  | *cln2Δ* | 33.9 |  | 18.6 |  | 79.8 |  | 59.3 |  |
|  | *cln1Δ cln2Δ* | 38.8 |  | 32.0 |  | 72.3 |  | 73.4 |  |
| *T*_bir,_*_CLB2_* | WT | 24.1 | 19.0 | 11.67 | 13.0 | 78.0 |  | 37.1 |  |
|  | *cln1Δ* | 36.8 |  | 22.0 |  | 76.7 |  | 66.2 |  |
|  | *cln2Δ* | 33.9 |  | 19.0 |  | 80.5 |  | 60.0 |  |
|  | *cln1Δ cln2Δ* | 41.0 | 32.0 | 35.7 | 29.0 | 73.8 |  | 78.0 |  |
| *T*_C_*_LN2,RAD27_* | WT | 3.0 | 11.0 | 2.0 | 10.0 | 15.1 |  | 5.7 |  |
|  | *cln1Δ* | 6.1 |  | 4.7 |  | 13.0 |  | 11.0 |  |
|  | *cln2Δ* | 6.8 |  | 4.5 |  | 18.5 |  | 10.4 |  |
|  | *cln1Δ cln2Δ* | 19.2 | 23.0 | 20.3 | 20.0 | 31.0 |  | 38.3 |  |
| *T_CLN2,_*_Whi5_ | WT | 11.0 | 11.0 | 5.1 | 5.0 | 44.4 |  | 16.9 |  |
|  | *cln1Δ* | 18.8 |  | 11.1 |  | 61.9 |  | 34.2 |  |
|  | *cln2Δ* | 18.6 |  | 9.6 |  | 76.2 |  | 32.7 |  |
|  | *cln1Δ cln2Δ* | 26.0 | 26.0 | 19.9 | 20.0 | 75.2 |  | 48.7 |  |

**Table H:** Average and CV of various cell cycle properties from stochastic simulations of wild-type (WT) and mutant cells growing on glucose medium (μ = 0.007 min^−1^). The volume and the time are in fL and min units, respectively.

| **Daughter cell** | | | | | | | | | | |
| --- | --- | --- | --- | --- | --- | --- | --- | --- | --- | --- |
| Average | | | | | | | | | | |
|  | WT | *cln1Δ* | *cln2Δ* | *clb5Δ* | *cdh1Δ* | *sic1Δ* | *swi5Δ* | *cln1Δ cln2Δ* | *clb5Δ clb6Δ* | *cln1Δ cln2Δ sic1Δ* |
| $V_{\mathrm{bir}}$ | 27.1 | 39.1 | 34.2 | 37.0 | 28.2 | 25.8 | 26.2 | 76.6 | 45.6 | 49.5 |
| $V_{\mathrm{div}}$ | 57.2 | 82.3 | 72.4 | 77.6 | 68.9 | 53.5 | 54.8 | 160.6 | 96.8 | 103.9 |
| $T_{\mathrm{div}}$ | 108.1 | 107.9 | 108.6 | 107.1 | 119.6 | 105.1 | 107.6 | 107.6 | 108.4 | 106.7 |
| $T_{Whi5}$ | 21.0 | 14.1 | 17.0 | 11.8 | 37.7 | 23.9 | 23.8 | 2.9 | 7.5 | 10.0 |
| $T_{\mathrm{unbud}}$ | 37.1 | 34.1 | 37.7 | 24.9 | 55.8 | 39.4 | 38.9 | 40.6 | 17.9 | 43.7 |
| $T_{g1}$ | 43.8 | 39.6 | 42.1 | 54.6 | 55.0 | 38.0 | 39.0 | 32.3 | 70.1 | 18.6 |
| $T_{\mathrm{bud}}$ | 70.9 | 73.8 | 70.9 | 82.3 | 63.7 | 65.6 | 68.1 | 67.0 | 90.5 | 63.1 |
| Coefficient of variation | | | | | | | | | | |
| $V_{\mathrm{bir}}$ | 0.25 | 0.28 | 0.25 | 0.38 | 0.73 | 0.23 | 0.25 | 0.37 | 0.33 | 0.22 |
| $V_{\mathrm{div}}$ | 0.2 | 0.22 | 0.19 | 0.36 | 0.96 | 0.19 | 0.2 | 0.36 | 0.29 | 0.19 |
| $T_{\mathrm{div}}$ | 0.21 | 0.2 | 0.21 | 0.28 | 0.44 | 0.21 | 0.21 | 0.28 | 0.19 | 0.16 |
| $T_{Whi5}$ | 0.87 | 1.04 | 0.98 | 1.12 | 0.75 | 0.82 | 0.84 | 1.27 | 1.76 | 0.96 |
| $T_{\mathrm{unbud}}$ | 0.56 | 0.57 | 0.56 | 0.68 | 0.62 | 0.55 | 0.57 | 0.68 | 0.48 | 0.33 |
| $T_{g1}$ | 0.45 | 0.5 | 0.49 | 0.38 | 0.53 | 0.41 | 0.43 | 0.41 | 0.70 | 0.39 |
| $T_{\mathrm{bud}}$ | 0.15 | 0.16 | 0.15 | 0.29 | 0.66 | 0.17 | 0.17 | 0.29 | 0.18 | 0.21 |
| **Mother cell** | | | | | | | | | | |
| Average | | | | | | | | | | |
| $V_{\mathrm{bir}}$ | 40.0 | 57.8 | 50.4 | 54.1 | 38.9 | 37.3 | 38.3 | 112.1 | 65.6 | 68.5 |
| $V_{\mathrm{div}}$ | 73.9 | 108.3 | 93.9 | 103.2 | 86.3 | 68.2 | 70.6 | 223.5 | 128.0 | 133.8 |
| $T_{\mathrm{div}}$ | 88.5 | 90.4 | 89.4 | 92.3 | 101.4 | 86.8 | 88.1 | 99.2 | 95.5 | 95.8 |
| $T_{Whi5}$ | 7.1 | 4.3 | 5.2 | 4.2 | 17.9 | 8.7 | 8.5 | 0.7 | 2.9 | 4.0 |
| $T_{\mathrm{unbud}}$ | 16.6 | 15.1 | 16.4 | 11.7 | 32.2 | 18.4 | 17.8 | 28.3 | 9.2 | 27.8 |
| $T_{g1}$ | 25.3 | 22.7 | 23.7 | 37.7 | 32.8 | 21.0 | 21.5 | 21.7 | 55.2 | 11.1 |
| $T_{\mathrm{bud}}$ | 71.9 | 75.3 | 73.1 | 80.6 | 69.2 | 68.4 | 70.3 | 70.8 | 86.4 | 67.9 |
| Coefficient of variation | | | | | | | | | | |
| $V_{\mathrm{bir}}$ | 0.25 | 0.28 | 0.25 | 0.37 | 0.55 | 0.23 | 0.25 | 0.35 | 0.33 | 0.21 |
| $V_{\mathrm{div}}$ | 0.22 | 0.26 | 0.23 | 0.38 | 0.87 | 0.22 | 0.23 | 0.36 | 0.32 | 0.21 |
| $T_{\mathrm{div}}$ | 0.16 | 0.15 | 0.16 | 0.26 | 0.5 | 0.16 | 0.16 | 0.26 | 0.15 | 0.13 |
| $T_{Whi5}$ | 0.94 | 0.97 | 1 | 1.15 | 0.88 | 0.9 | 0.9 | 1.32 | 3.54 | 0.82 |
| $T_{\mathrm{unbud}}$ | 0.54 | 0.46 | 0.53 | 0.6 | 0.76 | 0.57 | 0.57 | 0.74 | 0.44 | 0.24 |
| $T_{g1}$ | 0.35 | 0.35 | 0.37 | 0.31 | 0.49 | 0.36 | 0.36 | 0.41 | 0.66 | 0.29 |
| $T_{\mathrm{bud}}$ | 0.15 | 0.15 | 0.15 | 0.29 | 0.67 | 0.16 | 0.16 | 0.28 | 0.18 | 0.18 |

**Table I:** Average and CV of various cell cycle properties from stochastic simulations of wild-type (WT) and mutant cells growing on galactose medium (μ = 0.00467 min^−1^). The volume and the time are in fL and min units, respectively.

| **Daughter cell** | | | | | | | | | | |  |
| --- | --- | --- | --- | --- | --- | --- | --- | --- | --- | --- | --- |
| Average | | | | | | | | | | |  |
|  | WT | *GAL-CLB2* | *GAL-CLB5* | *GAL-SIC1* | *cln1Δ cln2Δ GAL-CLN2* | *clb2-dbΔ GAL-SIC1* | *clb2-dbΔ* Multi*-SIC1* | *clb2-dbΔ clb5Δ* | *swi5Δ cdh1Δ GAL-SIC1* | *GAL-NET1 GAL-CDC14* | *cdh1Δ* |
| $V_{\mathrm{bir}}$ | 17.1 | 12.8 | 17.6 | 19.2 | 27.6 | 23.1 | 17.5 | 33.8 | 20.4 | 16.8 | 21.6 |
| $V_{\mathrm{div}}$ | 38.6 | 31.4 | 37.3 | 42.2 | 56.5 | 50.4 | 39.6 | 73.5 | 50.2 | 39.1 | 52.5 |
| $T_{\mathrm{div}}$ | 175.5 | 191.6 | 161.7 | 171.4 | 157.5 | 171.2 | 176.3 | 166.9 | 190.1 | 181.7 | 190.7 |
| $T_{Whi5}$ | 61.9 | 121.9 | 59.4 | 41.2 | 15.1 | 46.4 | 65.2 | 27.9 | 77.0 | 66.6 | 92.0 |
| $T_{\mathrm{unbud}}$ | 91.0 | 147.6 | 82.5 | 76.1 | 81.3 | 68.6 | 90.6 | 47.7 | 103.3 | 96.3 | 116.0 |
| $T_{g1}$ | 96.1 | 151.5 | 76.2 | 87.2 | 65.9 | 78.5 | 102.7 | 77.5 | 108.5 | 101.3 | 113.0 |
| $T_{\mathrm{bud}}$ | 84.5 | 44.1 | 79.1 | 95.2 | 76.2 | 102.6 | 85.7 | 119.2 | 86.9 | 85.4 | 74.6 |
| Coefficient of variation | | | | | | | | | | |  |
| $V_{\mathrm{bir}}$ | 0.24 | 0.19 | 0.23 | 0.29 | 0.29 | 0.54 | 0.26 | 0.66 | 0.72 | 0.24 | 0.88 |
| $V_{\mathrm{div}}$ | 0.21 | 0.23 | 0.21 | 0.22 | 0.2 | 0.5 | 0.22 | 0.73 | 0.89 | 0.21 | 1.07 |
| $T_{\mathrm{div}}$ | 0.32 | 0.33 | 0.33 | 0.32 | 0.28 | 0.38 | 0.31 | 0.46 | 0.44 | 0.31 | 0.43 |
| $T_{Whi5}$ | 0.87 | 0.5 | 0.88 | 1.22 | 1.58 | 0.97 | 0.76 | 1.3 | 0.76 | 0.82 | 0.72 |
| $T_{\mathrm{unbud}}$ | 0.59 | 0.41 | 0.61 | 0.67 | 0.51 | 0.73 | 0.57 | 0.95 | 0.61 | 0.56 | 0.59 |
| $T_{g1}$ | 0.54 | 0.4 | 0.62 | 0.58 | 0.67 | 0.64 | 0.5 | 0.62 | 0.6 | 0.52 | 0.59 |
| $T_{\mathrm{bud}}$ | 0.16 | 0.12 | 0.19 | 0.18 | 0.23 | 0.38 | 0.18 | 0.52 | 0.65 | 0.17 | 0.88 |
| **Mother cell** | | | | | | | | | | |  |
| Average | | | | | | | | | | |  |
| $V_{\mathrm{bir}}$ | 29.6 | 22.3 | 29.4 | 33.1 | 45.0 | 39.6 | 30.5 | 56.2 | 34.9 | 29.3 | 35.8 |
| $V_{\mathrm{div}}$ | 50.8 | 35.6 | 49.6 | 57.6 | 80.4 | 72.7 | 52.5 | 107.9 | 64.7 | 50.9 | 70.1 |
| $T_{\mathrm{div}}$ | 117.2 | 101.4 | 113.1 | 120.7 | 125.5 | 128.4 | 118.1 | 135.2 | 124.8 | 120.3 | 158.9 |
| $T_{Whi5}$ | 15.1 | 37.8 | 15.4 | 9.0 | 3.6 | 13.4 | 18.6 | 8.1 | 25.3 | 17.3 | 30.2 |
| $T_{\mathrm{unbud}}$ | 31.2 | 56.5 | 30.5 | 25.2 | 40.9 | 25.3 | 32.3 | 18.0 | 42.7 | 33.5 | 47.4 |
| $T_{g1}$ | 39.6 | 62.0 | 32.2 | 37.5 | 35.7 | 35.9 | 47.1 | 41.7 | 44.8 | 41.8 | 47.1 |
| $T_{\mathrm{bud}}$ | 85.9 | 44.8 | 82.7 | 95.4 | 84.6 | 103.1 | 85.8 | 117.2 | 82.1 | 86.8 | 79.6 |
| Coefficient of variation | | | | | | | | | | |  |
| $V_{\mathrm{bir}}$ | 0.24 | 0.19 | 0.23 | 0.29 | 0.28 | 0.49 | 0.26 | 0.63 | 0.64 | 0.25 | 0.86 |
| $V_{\mathrm{div}}$ | 0.2 | 0.17 | 0.2 | 0.25 | 0.26 | 0.58 | 0.22 | 0.7 | 0.79 | 0.21 | 0.99 |
| $T_{\mathrm{div}}$ | 0.24 | 0.33 | 0.22 | 0.23 | 0.17 | 0.37 | 0.24 | 0.48 | 0.49 | 0.26 | 0.53 |
| $T_{Whi5}$ | 1.35 | 0.8 | 1.27 | 1.84 | 1.39 | 1.11 | 1.01 | 1.29 | 0.93 | 1.36 | 0.96 |
| $T_{\mathrm{unbud}}$ | 0.77 | 0.58 | 0.72 | 0.79 | 0.48 | 0.83 | 0.7 | 1.08 | 0.82 | 0.8 | 0.79 |
| $T_{g1}$ | 0.58 | 0.53 | 0.61 | 0.53 | 0.56 | 0.56 | 0.47 | 0.53 | 0.63 | 0.61 | 0.63 |
| $T_{\mathrm{bud}}$ | 0.17 | 0.12 | 0.18 | 0.19 | 0.19 | 0.42 | 0.19 | 0.54 | 0.62 | 0.17 | 0.78 |

**Table J:** Effects of binomial partitioning of molecules and external noise at division on the variability of cell cycle properties. At division, protein and mRNA molecules are partitioned proportionately to mother and daughter cells according to their respective cell sizes (second column) and also according to a binomial random process with probability given by the relative sizes of the mother and daughter cells (third column). Extrinsic noise is introduced in the division fraction *f* assuming that it follows Gaussian distribution with mean *f* and different values of CV. We used 5% and 10% variation in the value of *f* (fourth and fifth columns).

| Variability (Coefficient of Variation) | | | | |
| --- | --- | --- | --- | --- |
| Daughter cells | | | | |
| Property | Source of noise | | | |
|  | Partitioning according to size | Binomial partitioning | binomial partitioning with 5% division noise | binomial partitioning with 10% division noise |
| $V_{\mathrm{bir}}$ | 0.25 | 0.24 | 0.25 | 0.27 |
| $V_{\mathrm{div}}$ | 0.2 | 0.19 | 0.20 | 0.21 |
| $T_{\mathrm{div}}$ | 0.21 | 0.22 | 0.21 | 0.22 |
| $T_{Whi5}$ | 0.87 | 0.79 | 0.79 | 0.79 |
| $T_{\mathrm{unbud}}$ | 0.56 | 0.56 | 0.56 | 0.58 |
| $T_{g1}$ | 0.45 | 0.46 | 0.45 | 0.47 |
| $T_{\mathrm{bud}}$ | 0.15 | 0.15 | 0.15 | 0.15 |
| Mother cells | | | | |
| $V_{\mathrm{bir}}$ | 0.25 | 0.24 | 0.25 | 0.27 |
| $V_{\mathrm{div}}$ | 0.22 | 0.22 | 0.22 | 0.24 |
| $T_{\mathrm{div}}$ | 0.16 | 0.17 | 0.16 | 0.17 |
| $T_{Whi5}$ | 0.94 | 0.89 | 0.82 | 0.89 |
| $T_{\mathrm{unbud}}$ | 0.54 | 0.60 | 0.54 | 0.58 |
| $T_{g1}$ | 0.35 | 0.38 | 0.35 | 0.38 |
| $T_{\mathrm{bud}}$ | 0.15 | 0.16 | 0.15 | 0.15 |

**Table K**: Oligonucleotides used to target GFP-tagged cell cycle genes and the *CLN2* gene.

| **Probe ID** | **Sequence*** |
| --- | --- |
| GFP-70 | **T***CCGTATGT**T***GCATCACCT**T***CACCCTCTCCAC**T***GACAGAAAATTTG**T***GCCC |
| GFP-304 | GGG**T***ATCACCTTCAAAC**T***TGACTTCAGCACG**T***GTCTTGTAG**T***TCCCGTCA**T***C |
| GFP-555 | **T***GGACAGGTAA**T***GGTTGTCTGG**T***AAAAGGACAGGGCCA**T***CGCCAATTGGAG**T***A |
| GFP-628 | GCAGC**T***GTTACAAAC**T***CAAGAAGGACCA**T***GTGGTCTCTC**T***TTTCGTTGGGA**T***C |
| GFP-682 | **T***AGAAGTGGCGCGCCC**T***ATTTGTATAG**T***TCATCCATGCCA**T***GTGTAATC**T***C |
| CLN2-231 | AA**T***GTTGGACCTTG**T***TTCCACGGGG**T***TCATTTCAGGC**T***GCTGGTCT |
| CLN2-377 | AACCAGAGACAAG**T***AGCGACAACCAATT**T***GGCTTGGTCCCG**T***AACACG |
| CLN2-440 | CCGCCAG**T***AGGGATGAC**T***ACATTATTGA**T***GATGTGAT**T***ACAACCGCCCC |
| CLN2-495 | AAC**T***AGTTCAGAGAG**T***CGAGGTATACG**T***GCCCTTGGGT**T***GGGACCA**T***A |
| CLN2-742 | AGGGCCTCTCGTC**T***ACAGTGGCA**T***CACTATCC**T***GGGATAAT**T***GAGA |
| CLN2-1601 | AAAGA**T***GAGGCACTGC**T***AGATTTACCGC**T***ATTTATGGTCCCAG**T***TGGCGAGGGG |

*Nucleotides in bold are modified with amino groups for conjugation to a fluorophore (DyLight 550 for GFP oligos and DyLight 594 for *CLN2* oligos). While all the GFP probes have five fluorophores, the sequence of *CLN2* limited the number of fluorophores that could be added to some probes. In order to achieve a comparable signal between the two genes, six probes were used to label *CLN2*.

**References**

Di Talia S, Skotheim JM, Bean JM, Siggia ED, Cross FR (2007) The effect of molecular noise and size control on the variability in the budding yeast cell cycle. *Nature* **448:** 947-951

Skotheim JM, Talia DT, Siggia ED, Cross FR (2008) Positive feedback of G1 cyclins ensures coherent cell cycle entry. *Nature* **454:** 291-296
