# Supplementary material for: A Stochastic Model of the Yeast Cell Cycle Reveals Roles for Feedback Regulation in Limiting Cellular Variability
Source: PLoS Comput Biol. 2016 Dec 9;12(12):e1005230. doi: 10.1371/journal.pcbi.1005230 (PMC5147779; doi:10.1371/journal.pcbi.1005230)
Supplement: S1 Data Set — (DOCX) [file pcbi.1005230.s002.docx]

**Data Set File to:**

**Feedback regulation minimizes cell-cycle variability in budding yeast:**

**a stochastic model**

Debashis Barik, David A. Ball, Jean Peccoud and John J. Tyson

**Deterministic simulations for all the strains listed in Supplementary Table 4.**
